# Barriers to and Facilitators of Implementation of Internet-Delivered Therapist-Guided Therapy in Child and Adolescent Mental Health Services: Systematic Review and Bayesian Meta-Analysis

**DOI:** 10.2196/83543

**Published:** 2025-12-22

**Authors:** Annika Sannes, Erling W Rognli, Ketil Hanssen-Bauer, Nor Christian Torp, Silje Klundelien Storfossen, Markus Nordheim Høstaker, Marianne Aalberg

**Affiliations:** 1 Division of Mental Health Care and Addiction, Akershus University Hospital, Postboks 1000, Lørenskog, Akershus, 1478, Norway, 47 67960000; 2 Institute of Clinical Medicine, Faculty of Medicine, University of Oslo, Oslo, Norway; 3 Vestre Viken Hospital Trust, Bærum, Vestre Viken, Norway

**Keywords:** systematic review, child and mental health care services, implementation, facilitators and barriers, internet-based cognitive behavioral therapy, iCBT, e-therapy

## Abstract

**Background:**

Internet-delivered therapist-guided therapy (e-therapy) represents a promising approach for enhancing accessibility, treatment fidelity, and scalability within child and adolescent mental health services (CAMHS).

**Objective:**

This systematic review aimed to (1) identify and synthesize determinants of implementation, specifically barriers to and facilitators of e-therapy in CAMHS structured according to the Consolidated Framework of Implementation Research (CFIR); and (2) provide pooled benchmark estimates of key implementation outcomes for fidelity, cost-effectiveness, and acceptability.

**Methods:**

A PRISMA (Preferred Reporting Items for Systematic Reviews and Meta-Analyses)–compliant systematic review was performed across PsycINFO, MEDLINE, Web of Science, CINAHL, Embase, Cochrane, and ProQuest Dissertations & Thesis on June 6, 2025—to identify peer-reviewed studies assessing implementation outcomes or determinants of e-therapy in the context of outpatient CAMHS (ages 8‐18 years). Barriers and facilitators were synthesized qualitatively with thematic analysis applying CFIR. A parallel quantitative synthesis of Proctor et al’s taxonomy of implementation outcomes was performed using Bayesian multilevel random-effects meta-analyses to estimate pooled effect sizes and 95% credible intervals (CIs). By combining quantitative benchmarks of implementation success with qualitative insights into contextual determinants, the review provides an integrated understanding of what drives effective e-therapy implementation in CAMHS. Study quality was assessed using the CASP (Critical Appraisal Skills Programme) checklist, Cochrane Risk of Bias tool, and Risk Of Bias In Non-randomized Studies–of Interventions tool. Small study effects were evaluated using funnel plots, sensitivity analyses, and the Egger test.

**Results:**

From 50,026 screened reports, 50 studies published between 2007 and 2025 were included: 18 randomized controlled trials, 17 cohort, and 15 qualitative or mixed methods studies. Most studies originated from Western Europe (n=34), Northern America (n=11), and Oceania (n=5), targeting anxiety (n=24) and depression (n=9), through cognitive behavioral therapy–based programs (n=47), with parallel parent content (n=31). Therapist guidance was primarily asynchronous (n=43). Among the 39 studies reporting determinants, common barriers and facilitators were identified across intervention, organization, therapist, and patient domains, structured via CFIR. Pooled implementation outcomes showed modest dropout rates (~20%, CI 14%‐27%), high module completion (~68%, CI 60%‐75%), low therapist time (24 min per wk per patient, 95% CI 19‐28), and high patient satisfaction (24/32 on Client Satisfaction Questionnaire-8, 95% CI 22‐27; and 76% satisfaction rate, 95% CI 62%‐87%), suggesting e-therapy is resource efficient and acceptable if implemented successfully.

**Conclusions:**

This review provided the first integrated synthesis of pooled benchmarks for implementation outcomes of e-therapy in CAMHS and modifiable determinants to inform future service planning and scale-up. These findings highlighted service-level enablers, such as leadership anchoring, targeted use, technical stability, structured patient flow, and therapist training, that organizations could prioritize to strengthen sustainable e-therapy implementation in CAMHS.

## Introduction

### Background

The availability of evidence-based treatments for mental health issues among children and adolescents has grown substantially over recent years. However, the reach and delivery to young people in need are still limited by insufficient resources in mental health services, geographic distances between patients and health care providers, and socioeconomic differences [1]. Digitalization efforts in mental health care show great potential to make evidence-based treatment more accessible and scalable, while also supporting high intervention fidelity [2-5]. Indeed, research on internet-delivered therapist-guided therapy (e-therapy) shows encouraging empirical support for being a clinical and cost-effective method of delivering evidence-based treatment across various mental disorders, age groups, levels of symptom severity, and contextual settings [4-9]. Despite the encouraging empirical support, as well as enthusiasm regarding eHealth from funding agencies and policymakers, the implementation of e-therapy remains slow, particularly within child and adolescent mental health services (CAMHS) [10]. Knowledge about what hinders and facilitates implementation of e-therapy in routine clinical practice was scarce [1]. Consequently, there was a critical need for systematic research to better understand determinants of successful implementation of e-therapy within standard care settings [11].

Implementation research can be defined as the scientific evaluation of strategies or methods used to support the integration of evidence-based practices or programs into health care settings to enhance the quality and effectiveness of services [12]. Implementation research within mental health care can help solve the challenges of acceptability, adoption, costs, feasibility, fidelity, and sustainability in translating mental health interventions from research into routine practice [13,14]. However, implementation extent, quality, barriers, and facilitators are not routinely measured and reported in experimental and observational psychotherapy research [1,15-17]. When reported, implementation outcomes and determinants lack a unified operationalization [1,18,19]. Thus, filling the gap between research and real-world practice depends on effectively navigating varying operationalizations and definitions of implementation measurements and multiple levels of potential barriers and facilitators for successful implementation.

To address the conceptual heterogeneity in implementation research, this review applies established theoretical frameworks to provide standardized terminology and enhance interpretability across studies. Proctor et al’s taxonomy of implementation outcomes is used to operationalize implementation success in terms of fidelity, cost-related resource implications, and acceptability [13]. Fidelity is widely defined as the degree to which an intervention is implemented as intended and encompasses both therapist fidelity to a therapy manual or protocol and patient fidelity measured in terms of penetration, adherence, engagement, compliance, dropout, retention, attrition, and similar measures of degrees or proportions of the implementation extent and quality. Cost-effectiveness is defined as all measures related to organizational costs reported. Stakeholder acceptance is defined as all reports of patient, parent, and therapist experience of agreeableness or satisfaction with a given intervention. To synthesize determinants of implementation, this review uses the updated Consolidated Framework for Implementation Research (CFIR) theoretical model to synthesize research on implementation barriers and facilitators [20]. The CFIR is a well-documented theoretical framework that describes implementation processes with 37 constructs categorized into five major domains: intervention characteristics, outer setting, inner setting, characteristics of the individuals involved, and the implementation process.

Previous reviews have been conducted on development, implementation outcomes, drivers, modes of delivery, and engagement strategies of digital self-help mental health interventions for youths [10,15,21,22]. However, no comprehensive review exists that synthesizes implementation barriers and facilitators within a CFIR categorization, while also pooling implementation outcomes and focusing on therapist-assisted e-therapy within the context of CAMHS. Systematic reviews focusing on adult populations have identified barriers and facilitators operating at various levels, emphasizing particularly the attitudes, expectations, and preferences of both patients and professionals [23,24]. Interventions in child and adolescent mental health differ markedly from those designed for adults in several respects, especially regarding developmental considerations, the involvement of caregivers, and the influence of contextual factors such as peer pressure, family dynamics, and academic stress. There is an increasing emphasis on parental guidance and using play and creative methodologies to nurture the therapeutic alliance [25-27]. Consequently, there is a need for a systematic review specifically addressing determinants of implementation efforts in a CAMHS setting, given the unique infrastructures [28].

### Objectives

The objective of this systematic review was to identify and synthesize existing empirical research on the barriers and facilitators affecting the implementation of e-therapy within CAMHS. The focus was on the factors influencing the following implementation outcomes: intervention fidelity, stakeholder acceptance, and cost-effectiveness. This represented the first systematic review to apply the CFIR framework to therapist-guided e-therapy in CAMHS while concurrently generating pooled benchmark estimates for key implementation indicators in terms of fidelity, cost-effectiveness, and acceptability.

## Methods

This systematic review was reported following the PRISMA-S (Preferred Reporting Items for Systematic Review and Meta-Analyses literature search extension) statement 2020 guidelines (Checklist 1) [29,30] and was preregistered on PROSPERO 2024 CRD42024502578. No major amendments were made to the protocol.

### Eligibility Criteria

Quantitative, qualitative, and mixed-method, peer-reviewed, primary empirical studies were included if they reported implementation outcomes or barriers and facilitators of the implementation of e-therapy in the context of CAMHS. No limits were used on publication year or language in the selection of studies to provide a comprehensive presentation of the literature. The full inclusion and exclusion criteria are listed in Textbox 1.

Textbox 1. **Eligibility criteria.**

**Inclusion criteria**

- Participants

-  Therapists, staff, and patients of outpatient child and adolescent mental health services (CAMHS), including primary, secondary, and tertiary care services offering health care.
-  >50% of the patients treated at CAMHS aged between 8 to 18 years.

- Intervention

-  Studies reporting experienced barriers to and facilitators of and implementation outcomes in terms of fidelity, cost-effectiveness, and stakeholder acceptance of internet-delivered therapist-guided psychotherapy (e-therapy) in CAMHS.
-  e-Therapy is defined in this study as mental health treatment programs with digital delivery, including blended interventions (online therapy delivered alongside face-to-face, phone, or video sessions) as long as the treatment delivery is presented as mostly (>50%) digital, where the patient and parent receive feedback and guidance from a therapist on the intervention.

- Study type

-  Mixed methods studies
-  Qualitative studies (eg, qualitative interviews, focus groups, ethnographic observations, and qualitative case studies).
-  All primary empirical quantitative studies (eg, survey studies, randomized controlled trials, nonrandomized controlled trials with or without a control group, cohort or case control studies, and cross-sectional studies).

- Study design: studies of all designs that are peer-reviewed (including conference paper abstracts and theses)
- Publication: no limitation
- Language: no limitation

**Exclusion criteria**

- Participants

-  School-based and child welfare service–based implementation studies are excluded as the intervention context is specific to an education setting as opposed to a mental health service setting.

- Intervention

-  Studies regarding evaluation, development, and assessment of e-therapy programs that have not been implemented yet.
-  Synchronous, face-to-face therapy conducted exclusively via teleconferencing platforms.

- Study type

-  Systematic reviews, books, and meta-analyses are excluded, but their included primary studies will be assessed for eligibility.
-  Nonempirical work (eg, editorials, periodicals, opinion texts, and theoretical discussions).

- Study design: protocols

### Search Strategy

A search strategy was developed by the first author and reviewed by a research librarian. Searches were performed in parallel in the following electronic databases and study registries in January 2024 and updated in June 2025: PsycINFO (Ovid), MEDLINE (Ovid), Web of Science (Clarivate), CINAHL (EBSCOhost), Embase (Ovid), Cochrane CENTRAL (Wiley), and ProQuest Dissertations & Thesis (Clarivate). Multimedia Appendix 1 provides a complete search strategy. No published search filters were used. Duplicates were removed by a research librarian using EndNote software (version 2025.1; Clarivate).

### Study Selection

As e-therapy and implementation research are emerging and heterogeneous research fields, a wide search strategy was applied to capture a high number of possible relevant studies. The workload of the screening phase was assisted by the open-source artificial intelligence screening tool ASReview, which dynamically presents relevant articles by continuously adjusting the order of articles based on the reviewers’ previous decisions to include or exclude articles. We applied a conservative approach with multiple stopping rules for the artificial intelligence–assisted article screening process, following the SAFE procedure [31]. For further details, see Multimedia Appendix 2. Four reviewers screened titles and abstracts to determine whether they met the eligibility criteria (Textbox 1). Each article was screened independently by 2 reviewers. After the first screening, 2 reviewers independently reassessed all included articles in full text for further exclusion of noneligible studies. All disagreements were resolved through discussion until a consensus was reached. We also screened reference lists of the included studies by browsing to ensure that we retrieved all studies relevant to this review. Where available, protocols of included studies were searched for additional study publications. Searches were rerun on June 6, 2025, applying the same procedure for study selection.

### Data Extraction

Data were independently extracted and verified. Disagreements were resolved by discussion. One study author was contacted to provide missing data. Variables included study characteristics, intervention features, implementation outcomes, and reported barriers and facilitators. Full extraction fields and definitions are provided in Multimedia Appendix 3.

### Quality Assessment

Two reviewers independently assessed study quality (CASP [Critical Appraisal Skills Programme] for qualitative studies and Cochrane Risk of Bias tool for randomized controlled trials [RCTs], and Risk Of Bias In Non-randomized Studies–of Interventions for nonrandomized studies); details and domain-level results are provided in Multimedia Appendix 2. They discussed discrepancies until a consensus was reached.

### Data Analysis and Synthesis

This review used a parallel qualitative synthesis of CFIR determinants and a quantitative synthesis of implementation outcomes using different methodologies. No analyses are presented in this review addressing the relationship between the 2 strands of results because of inconsistent outcome metrics. Thus, the review presents complementary but separate strands of evidence.

### Qualitative Synthesis of Barriers and Facilitators

To better aggregate primary studies on barriers to and facilitators of e-therapy implementation in a systematic and comprehensive manner, this review applied the updated CFIR theoretical model [20]. The CFIR was chosen to guide this review because of its comprehensiveness, allowing for the categorization of diverse implementation factors across a variety of studies using standardized terminology, fostering improved understanding and application in clinical practice [32]. The CFIR’s comprehensive nature and its alignment with evidence-based implementation strategies enhanced its applicability for understanding barriers and facilitators in CAMHS specifically [33]. These attributes, as well as its utility in previous reviews in the context of health services, made it the most appropriate evaluation framework for this review.

The CFIR is a generalized framework, and its founders recommend users to adapt construct operationalization and selection for appropriate use [20]. We made 2 adaptations to the use of the CFIR constructs. First, the outer and inner setting domains were combined into the construct ‘characteristics of the organization,’ as outer setting factors were rarely reported in the included studies. Second, the ’characteristics of the individuals’ was divided into separate constructs: ‘characteristics of the therapists’ and ‘characteristics of the patients.’ Finally, the domain ‘implementation process’ was not used, as this was rarely reported systematically in the included studies.

The reporting of barriers and facilitators in the results section of each included study was inductively coded by the first author, involving the designation and application of summarizing labels that identified the meaning of text components extracted from each study. These initial data-driven codes were then categorized into themes that represented recurring ideas across studies relating to experienced barriers to or facilitators of implementation. Themes were checked by rereviewing the citations from each article to ensure accurate representation of the data. Furthermore, to tabulate and categorize the barriers to and facilitators for implementation from the eligible articles, we used the five overarching domains of the CFIR: intervention characteristics, outer setting, inner setting, characteristics of the individuals, and the implementation process. The first author allocated the overlapping themes reported across studies to the best-fitting CFIR domain, which was reviewed by KHB on an iterative basis. Example quotes for all themes from each article are presented in Multimedia Appendix 4 for transparency.

### Quantitative Synthesis of Implementation Outcomes

We pooled the results of quantitative data on implementation outcomes using random effects meta-analysis when 2 or more studies reported the same outcome. We included data from qualitative studies if they reported the number of participants for each outcome and the total number of participants. We used Bayesian random effects models with weakly informative prior distributions, which allow more stable estimates when only a few studies report an outcome. Such weakly informative prior distributions are specified to have about equal weight across the possible range of a model parameter, while ruling out parameter values that are impossible given reasonable assumptions. These priors are combined with the likelihood of the data to yield posterior distributions for model parameters. Posterior distributions thus give the probability of different parameter values, given the data and the model, in contrast to classical statistics, where inference is based on the probability of the data given a null hypothesis and assumed sampling distribution. The results of our analyses are presented as 95% credible intervals (CIs), which indicate the range within which the true value can be found with the corresponding probability, given the data and model [34] (see Multimedia Appendix 5 for prior specification). For several outcomes, reporting was not consistent across the included studies. The percentage of missing values across the 5 aggregated outcome variables varied between 0% and 82%. In total, 117 (53%) of 220 records were incomplete. To adjust for bias from missing evidence, multiple imputation of missing data was used [34-36]. For comparison, we also performed the analysis on the subset of complete cases. We assessed the quality of our models through convergence plots and posterior predictive checks. Detailed results from these diagnostics can be found in Multimedia Appendix 6. Further details on the Bayesian analyses are provided in Multimedia Appendix 2.

## Results

### Search Process

**Figure 1.**
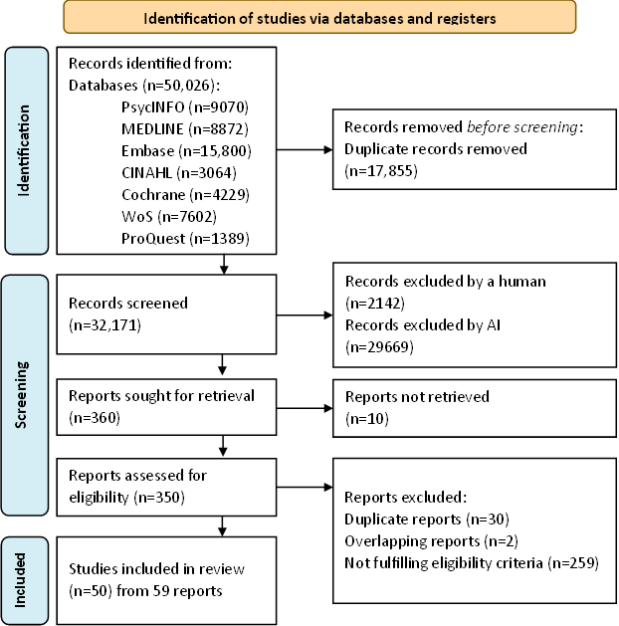
PRISMA (Preferred Reporting Items for Systematic Review and Meta-Analyses) flowchart of the literature search process. WoS: Web of Science.

The searches of the databases resulted in 32,171 reports after the removal of duplicates. Figure 1 provides a PRISMA flow diagram for the included studies. After full-text screening, 50 studies were included in the analyses. Where results from the same study sample were presented in multiple reports, we included the most comprehensive or most recent report. Where different relevant results from the same study with the same sample were presented in different reports, the results were included and combined for meta-analytic purposes.

### Review Statistics

Fifty studies (2007‐2025) were included, mostly European or North American cognitive behavioral therapy–based interventions targeting anxiety and depression. Approximately one-third were RCTs, most with parental components and asynchronous therapist guidance (see Multimedia Appendix 2 for full characteristics). The studies reporting implementation outcomes used different definitions and measurements. The most reported definitions and measurements used in the reviewed studies are shown in Table 1.

**Table 1.** Measurements and definitions of implementation outcomes (fidelity, cost-effectiveness, and acceptability) in the reviewed studies (n=50).

Fidelity measures	Values, n (%)
Therapy dropout rate, defined as:	46 (92)
Proportion of patients who did not complete 100% of the intervention, measured by patient self-report or the program activity log	20 (40)
Proportion of patients lost to follow-up	11 (22)
Proportion of patients who did not complete the predetermined amount of the intervention deemed necessary, ranging from 20% to 80%	7 (14)
Proportion of patients who actively withdrew from the intervention	6 (12)
Proportion of patients who were inactive during the treatment period	2 (5)
Mean proportion and SD of modules completed by the patients	31 (62)
Various, incomparable therapist adherence scales	5 (10)
Cost-effectiveness measures	
Mean and SD of total therapist time used for each e-therapy patient per week	15 (30)
Stakeholder acceptability measures	
Proportion of satisfied patients in the sample	10 (20)
Mean and SD of Client Satisfaction Questionnaire-8 scale	9 (18)
Various, incomparable patient satisfaction scales	15 (30)
Various, incomparable patient-, parent-, or therapist-rated program credibility, usability, and sustainability scales	18 (36)

Twenty-one studies reported facilitators, and 35 studies reported barriers to implementation of e-therapy in CAMHS (in total 39 studies): 11 RCT studies, 13 observational studies, and 15 studies mainly using qualitative data through surveys and interviews. The barriers and facilitators extracted from the studies were based on a range of stakeholders’ opinions, trained health care professionals (eg, psychologists, psychiatrists, nurses, and general practitioners), and patients and their caregivers. None of the included studies consulted policymakers or leadership. Almost all studies measured barriers and facilitators retrospectively. The studies provided insufficient information to aggregate proportional data on each barrier and facilitator and were mainly presented as a qualitative description of patients’ experiences stated in open survey questions from satisfaction measurements or qualitative interviews. Six studies reported the number of dropouts with data on reasons for patient withdrawal; however, not using the same barriers or facilitators needed for aggregation of proportions.

### Level of Evidence Summary

The extracted data from all included studies were assessed for quality independently by 2 researchers. Overall, study quality was moderate, with some concerns about fidelity measurement and missing user satisfaction data. Regression tests for funnel plot asymmetry showed significant findings for dropout and satisfaction rate. For dropout, most of the studies on the right of the funnel plot defined dropout as <100% program completion, whereas the studies on the left had fewer conservative definitions of dropout, which most likely explains the asymmetry. No reports were excluded based on the quality assessment to provide a comprehensive presentation of the literature. Detailed quality assessment results and funnel plots and regression tests for small study effects are presented in MultimediaAppendices 7  8.

### Quantitative Synthesis of Implementation Outcomes of E-Therapy in CAMHS

Bayesian models converged adequately (Multimedia Appendix 7). Pooled dropout of ≈20%, module completion of ≈68%, and satisfaction of ≈76% indicate good feasibility and acceptability. Forest plots and funnel plots are shown in Multimedia Multimedia Appendix 8, and a summary of the data extraction from each included study is shown in Multimedia Appendix 3. Table 2 presents the missing data rates of each variable, the mean and 95% CI of the posterior distribution for the pooled estimate, and the between-study heterogeneity. We obtained similar results when the analysis was restricted to complete cases only. Multiple imputation was generally more efficient, as can be seen from the shorter credible intervals in Table 2.

**Table 2.** Pooled parameter estimates of implementation outcomes (mean and credible interval [CI] and study heterogeneity [Tau]) with complete cases and multiple imputations from Bayesian random effects meta-analyses models with weakly informative priors of reviewed studies in Europe, America, and Asia from 2007 to 2025 on implementation of internet-delivered, therapist-guided therapy in child and adolescent mental health services (N=46)^a^.

Model and parameter	Posterior mean (95% CI)	
Treatment dropout (n=46 studies, 0% missing)^b^		
Overall estimate (log odds)^c^	−1.39 (−1.82 to −0.99)	
Between-study heterogeneity estimate (log odds)^c^	1.37 (1.06 to 1.76)	
Proportion of modules completed (n=31 studies, 32% missing)		
Overall estimate (proportion)	0.70 (0.64 to 0.77)	
Between-study heterogeneity estimate	0.16 (0.13 to 0.22)	
Adjusted proportion of modules completed (with multiple imputation, n=46 studies)		
Overall estimate (proportion)	0.68 (0.60 to 0.75)	
Between-study heterogeneity estimate	0.20 (0.15 to 0.25)	
Therapist time per patient per week (n=15 studies, 68 % missing)		
Overall estimate (min)	25.8 (20.4 to 31.8)	
Between-study heterogeneity estimate	11.4 (7.8 to 17.4)	
Adjusted therapist time per patient per week (with multiple imputation, n=46 studies)^d^		
Overall estimate (min)	24.0 (19.2 to 28.2)	
Between-study heterogeneity estimate	10.8 (8.4 to 13.8)	
Patient satisfaction (n=9 studies, 81% missing)		
Overall estimate (mean CSQ-8 score [scale 8‐31])	24.5 (22 to 26.5)	
Between-study heterogeneity estimate	3.4 (2.1 to 6.1)	
Adjusted patient satisfaction (with multiple imputation, n=9 studies, 81% missing)		
Overall estimate (mean Client Satisfaction Questionnaire-8 score [scale 8‐31])	24.5 (21.9 to 26.8)	
Between-study heterogeneity estimate	3.4 (2.0 to 6.0)	
Patient satisfaction rate (n=9, 81% missing)		
Overall estimate	0.75 (0.62 to 0.87)	
Between-study heterogeneity estimate	0.16 (0.08 to 0.29)	
Adjusted patient satisfaction rate (with multiple imputation, n=8, 81% missing)		
Overall estimate	0.76 (0.62 to 0.87)	
Between-study heterogeneity estimate	0.16 (0.08 to 0.30)	

a R version 4.1.1, CmdStan, brms package, 4 chains, 4000 iterations (2000 warmup and 2000 postwarmup draws). Multiple imputation models using Predictive Mean Matching from the Mice package with 20 samples.

b Four studies were excluded from the analysis due to study design (focus group study), not missing at random.

c Transformed to probability scale: 1.37 on the log scale implies a between-study variability between 0.06-0.49 (2SD), −1.39 (CI −1.82 to −0.99) translates to a mean probability of dropout of 0.2 (CI 0.14 to 0.27).

d Eight thousand iterations (4000 warmup and 4000 postwarmup draws).

### Secondary Analyses

Secondary sensitivity analyses were performed for pooled implementation outcome estimates of fidelity measures due to high between-study heterogeneity results. The analyses were stratified by time, design, and measurement operationalization, given rapid developments in digital delivery in the broad time span of reviewed studies, high variability in study design, and definition of treatment dropout rate. The results indicate some but highly uncertain effects of design (−0.10, CI −0.17 to 0.04) and lower heterogeneity for RCT studies (0.34, CI 0.27-0.45) compared to non-RCT studies (0.46, CI 0.37-0.59). Time had no effect on dropout estimates (0.02, CI −0.12 to 0.39) or the between-study heterogeneity (0.41, CI 0.31-0.58). Dropout definition had a moderate effect comparing a strict dropout definition (not completing 100% of the program, n=20) with other definitions (0.13, CI 0.00-0.32; see Multimedia Appendix 9 for secondary analyses), where the study heterogeneity was considerably higher for studies with a strict definition (0.50, CI 0.40-0.63) compared to others (0.36, CI 0.27-0.47).

Variability in implementation outcomes was high even when controlling for time, design, and measurement operationalization. The data did not allow quantitative analyses to assess the relationships with barriers and facilitators due to inconsistent metrics. The studies’ reporting of multilevel determinants for implementation success is therefore presented qualitatively.

### Qualitative Synthesis of CFIR Implementation Barriers and Facilitators

**Figure 2.**
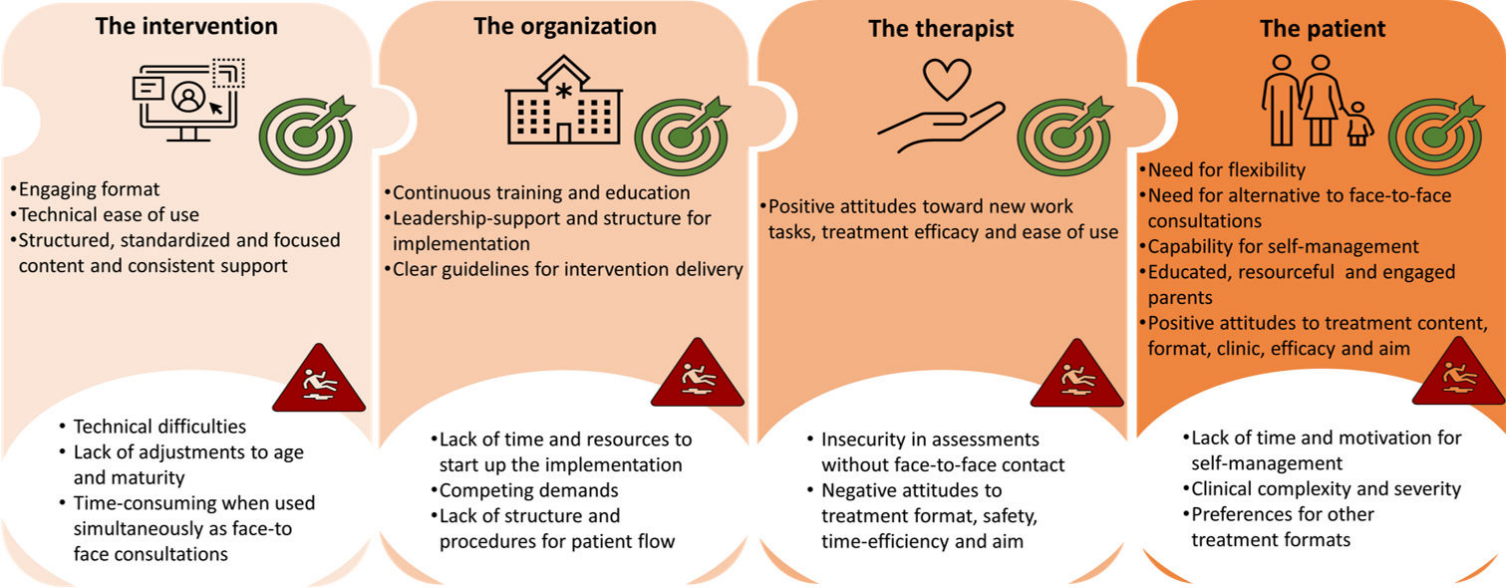
A visual summary of the qualitative synthesis of barriers and facilitators, which may impact implementation of internet-delivered, therapist-guided therapy in child and adolescent mental health services in the reviewed studies (2007‐2025), structured according to the Consolidated Framework of Implementation Research (n=39).

**Figure 3.**
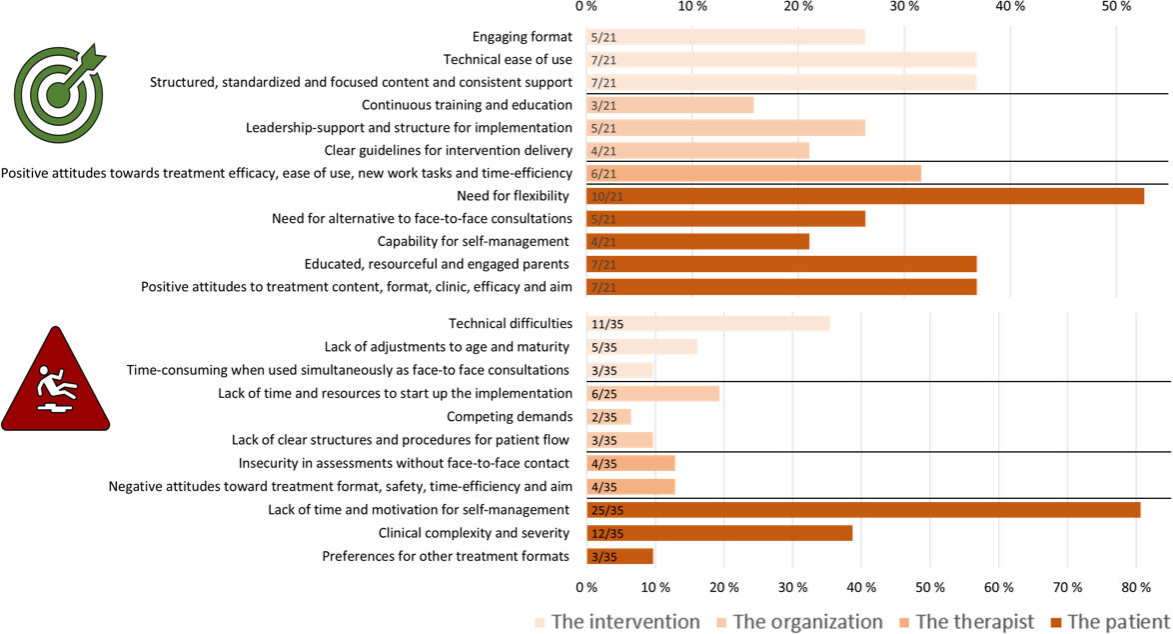
Proportion of the reviewed studies reporting each facilitator (n=21) and barrier (n=35) from the qualitative synthesis of implementation of internet-delivered, therapist-guided therapy in child and adolescent mental health services, structured according to the Consolidated Framework of Implementation Research (n=39, 2007‐2025).

We identified a wide variety of factors that covered 4 of the key CFIR domains. Figure 2 provides a visual summary of the identified barriers and facilitators corresponding to each of these domains. The proportion of studies reporting each factor among the studies that reported a barrier or a facilitator is presented in Figure 3.

### Domain 1: Intervention-Specific Factors

Patients, parents, and health care providers in CAMHS across the included studies reported several overlapping experiences of different characteristics of the e-therapy intervention program that influenced their use and satisfaction positively or negatively. A frequently reported barrier was technical difficulties, such as login issues, system crashes, and limited access to digital equipment [37-45]. Experiencing the program as unadjusted to the patients’ age and level of maturity, such as containing too much text or not enough interactive elements, was also a recurring theme in the studies [37,42,46-48]. Using the e-therapy program simultaneously as ordinary face-to-face consultations was described as a barrier in terms of being too time-consuming by some studies [47,49]. Among the reported positive characteristics of e-therapy that may facilitate implementation, several studies highlighted having a technical program that is easy to use [37,39,43,47,50,51]. Engaging elements of the digital format were also presented as important for satisfaction and use, such as interactive videos and exercises [38,40,49,51]. In addition, many studies highlighted the importance of standardization of the program to aid patients and professionals in focusing on the target problems, using the recommended interventions, and that the program provided a consistent therapeutic relationship [47-53].

### Domain 2: Organization-Specific Factors

Several barriers related to the characteristics of how the implementation of e-therapy was organized were highlighted by health care professionals across studies. Several studies reported experiences of a lack of time and resources required to implement a new intervention as an important barrier [46,49,52-54]. Many reported competing demands within the organization as a hindering factor for successful implementation, such as simultaneous priority implementation projects [49,52]. In addition, several health care professionals reported experiencing a lack of established or planned structures and procedures for patient flow when using the new intervention [49,52,54]. In terms of organizational facilitators, a recurrent theme was experiencing the implementation as supported by leadership [48,49,52,55,56]. A continuous focus on implementation with planned means of therapeutic development through education and educational material within the organization was also highlighted as a facilitator [48,49,52]. Furthermore, either co-developing or being provided with clear guidelines for intervention delivery and patient flow was pointed out as important for effective implementation [49,52,55,56].

### Domain 3: Therapist-Specific Factors

Health care providers across the included studies reported several factors related to the characteristics of the e-therapists that impacted satisfaction and use of the implemented interventions, both positively and negatively. First, several therapists reported feeling insecure about their competency in making sound health assessments digitally compared to face-to-face in terms of patients’ status and progress during the treatment [49,56-58]. Second, some health care providers reported having negative attitudes to digitalization, streamlining of treatment, and the safety of the patients without face-to-face consultations [47,52,55,59]. On the other hand, others reported positive attitudes toward implementation as a facilitator, such as wanting to learn something new and develop new therapeutic skills, seeking higher treatment efficacy when overworked, and experiencing digitalization as something that could ease their work-life balance [47,48,53,55,57].

### Domain 4: Patient-Specific Factors

The included studies that reported facilitators or barriers to implementation highlighted characteristics of the patients and their caregivers, and this also seemed to be the area that was mostly explored and focused on. The most reported barrier for implementation in terms of user satisfaction by health care providers and patients was the lack of time and self-management skills among some patients to complete digitally delivered therapy [37-42,44,45,47,49-51,56-58,60-67]. For example, many patients reported competing priorities related to school and friends that hindered them from following the intervention at home. Some health care providers reported that the patients’ clinical complexity and severity could be a barrier to offer e-therapy [38,42,46,50,56-58,64,67-69]. For example, some studies reported that patient groups with high comorbidity or elevated risk of suicide did not benefit from the e-therapy programs. In addition, some studies reported that patients who preferred other formats of therapy delivery, such as face-to-face consultations, were not suited for successful intervention implementation [49,67]. A recurrent facilitating patient characteristic was the need for flexibility to complete therapeutic tasks whenever and wherever it suited them [37,38,40,47,49,50,52,53,58]. Having resourceful, highly educated, and engaged parents to help the patients complete the treatment from home was also a recurrent facilitating theme [39,40,42,46,47,51,70]. This was mainly reported by health care providers, but some studies also showed that parents reported prioritizing assisting their child to complete the program as crucial. Some studies also found statistically significant relationships between parental education and income and treatment completion [51,70]. Patients’ capability for self-management was similarly reported as a facilitator, where health care providers and parents expressed that high-functioning or mature patients more easily used the programs and found them satisfying [38,47,49,50]. Family attitudes both before and during the therapy were reported as facilitators for treatment completion [38,42,50,51,53,58,71]. Such attitudes included positive views of the content and format of delivery, and positive attitudes toward the clinical assessments of the recommended treatment, expecting positive results and agreeing with the aim of the program format. Finally, patients, parents, and therapists across studies reported patients’ needs for an alternative to face-to-face treatment as an important facilitator for implementation, such as reducing stigma, travel time, or having to take time off from work [37,47,49,52].

## Discussion

### Principal Findings

This systematic review aimed to identify and synthesize barriers to and facilitators of e-therapy implementation in CAMHS using the CFIR framework and to provide pooled benchmark estimates of implementation outcomes. Thus, this review makes a dual contribution: first, by qualitatively mapping recurrent implementation determinants onto the CFIR framework to provide a structured understanding of modifiable barriers and facilitators and, second, by providing quantitative pooled benchmark that can inform future service planning, scale-up, and policy decisions. Consequently, the qualitative and quantitative syntheses together provide a complementary understanding of how contextual determinants influence implementation success.

### Implementation Outcomes

Across the included studies, findings indicated low therapist time per patient, high patient satisfaction, and moderate patient fidelity. The results showed consistently low costs in terms of therapist time per patient, high acceptability in terms of patient satisfaction, and moderate patient fidelity in terms of proportions of modules completed and therapy dropout. These results mirrored previous reviews of digital mental health interventions for youths and adults, which similarly demonstrated strong patient acceptability and cost-efficiency [6,8,21]. This review strengthens the empirical support for the argument that e-therapy provides patients with user-friendly, engaging content and support, and patient satisfaction in addition to cutting costs in the clinics. Other reviews pointed to possible challenges with patient adherence and intervention reach using a digital format corresponding to this review’s findings [10,15]. Nevertheless, the results of this review suggest that e-therapy has similar or lower dropout rates compared to face-to-face treatment delivery. Previous meta-analyses on face-to-face treatment reported dropout rates of 35.3% among adults [72], 28.8% in CAMHS studies [73], 14.6% in RCTs for child and youth depression [74], and 11.7% in RCTs for children with posttraumatic stress disorder [75]. Our findings do, however, demonstrate considerable between-study variability in fidelity measurements, such as dropout, underscoring the need to evaluate possible contextual determinants. Overall, considering plausible explanations for between-study differences in terms of time, study design, and definition, heterogeneity is persistently high and may arguably be informed by the synthesized implementation facilitators and barriers.

### Barriers and Facilitators Within the CFIR Framework

The synthesis of reported determinants for stakeholder acceptability, costs, and fidelity from multilevel informants provides implementation efforts and research valuable insights into recurring facilitators and barriers to implementation success. Mapping the reported determinants onto the CFIR domains revealed that barriers and facilitators were predominately situated at the organizational, therapist, and patient levels. The most frequently reported facilitators across the studies were related to therapists’ report of leadership support and structure for implementation, positive attitudes among the therapists about learning something new and trusting the digital format, and patients’ and therapists’ perception of the interventions’ ease of use and engaging format. Additionally, patients often expressed favorable views toward the increased flexibility offered by e-therapy approaches. The most frequently reported barriers were organizations’ lack of time and resources in an early phase of implementation, negative attitudes among therapists, and a lack of time and motivation among patients to engage with the intervention at home, as reported by therapists and parents. Additional barriers repeatedly cited were therapists’ observation of high symptom complexity and comorbidity, and both patient and therapist reports of technical difficulties contributing to reduced satisfaction with the intervention.

The findings of this study align with previous systematic reviews focused on adult populations regarding organizational and therapist-related barriers and facilitators. This suggests that existing implementation research on adult mental health services may provide valuable insights for the field of pediatric mental health [23]. For instance, systematic reviews of adult populations highlight clinician and patient attitudes and motivation as critical determinants of implementation success [76]. This aligns with the present reviews’ results, where negative attitudes among therapists were reported as a barrier and positive attitudes as a facilitator for implementation extent. Colleagues’ opinions and prior experiences have been systematically found to be more significant than published evidence in shaping views on new treatment models among clinicians working with adult patients, which may suggestively be applicable in efforts to solve implementation challenges in CAMHS [77]. Future implementation planning and evaluation should leverage the identified facilitators and barriers to tailor context-specific strategies for improved implementation success. The CFIR framework has been beneficially linked with a refined compilation of implementation strategies, the ERIC taxonomy, to tackle barriers through expert-panel polling and consensus processes on adult patient populations [78]. For example, findings indicate that implementation experts frequently recommend countering negative therapist attitudes about interventions through educational meetings and the identification of champions. They also advocate local consensus discussions to counter barriers related to competing implementation demands. Nonetheless, empirical evidence linking CFIR-identified barriers to specific ERIC strategies remains limited [79]. Despite this, using a systematic framework for recommended strategies can enhance complex decision-making by providing a structured approach.

Importantly, this review also presents unique findings specific to the CAMHS context, revealing qualitative differences in patient-related factors and the interventions’ characteristics. Technological barriers for implementation in adult mental health services are also common but differ in content as they highlight other themes, such as digital illiteracy, device limitations, navigation challenges, connectivity issues, preferences for longer sessions, and data privacy concerns [80]. Unique to the CAMHS context, parental support and resources were specifically reported as facilitating patient characteristics, unlike results from reviews on adult mental health services. In addition, negative therapist attitudes toward the possibility of forming a therapeutic alliance digitally, and e-therapy’s standardized format as incompatible with adult psychological interventions were not observed in the studies included in this review [23]. Previous research suggests that barriers to patient fidelity unique to the context of CAMHS require context-specific strategies [81].

### Implications for Practice and Policy

This review offers a unique contribution by identifying the most frequently reported CAMHS-specific facilitators and barriers to the implementation of e-therapy in a CFIR framework. By synthesizing existing implementation findings within a theoretical framework, this review provides stakeholders with a comprehensive understanding of the factors that can enhance implementation outcomes for e-therapy in CAMHS. Furthermore, the review offers pooled benchmarks for implementation outcomes that can guide service planning, such as allocating therapist time realistically, designing implementations to maximize adherence and acceptability, and anticipating common barriers. In doing so, it contributes to the advancement of a more accessible, efficient, and effective mental health care for young people. Applying frameworks, such as the CFIR, not only helps identify determinants but also provides opportunities for developing, planning, and evaluating adaptive strategies tailored to address these challenges effectively.

Future implementation efforts within CAMHS should incorporate real-time process evaluation to facilitate iterative refinement and ensure the sustainable scale-up of therapist-guided e-therapy within routine care. The barriers and facilitators identified in this review represent the most frequently reported determinants of successful implementation, as highlighted across the studies included. Among these, the necessity for leadership support and a structured framework for implementation is paramount; such measures are crucial for fostering continuous training and providing clear guidelines for service delivery. Establishing robust management practices not only ensures effective patient flow but can also cultivate therapist confidence over time. Moreover, therapist attitudes are identified as both facilitators and barriers, suggesting that the selection of appropriate therapists and interventions for changing attitudes toward the digital format should be considered closely in implementation efforts. Additionally, the determinants related to the perceptions of both patients and therapists regarding the programs’ engagement and ease of use underline the necessity for rigorous user testing and comprehensive technical support plans before implementation. While the standardized format of e-therapy offers flexibility, it may also be viewed as a barrier when combined with face-to-face therapy, indicating the importance of targeted use to keep therapist time low, and considering parental support and other factors to enhance adherence. By drawing on the insights synthesized in this review, implementation strategies can be strengthened, thus promoting long-term success in the provision of e-therapy within clinical practice.

### Recommendations for Future Research

Overall, the included studies demonstrate a need for more nuanced and consistent reporting standards for implementation processes and outcomes, as noted in previous reviews [21,82]. Furthermore, the results indicated that studies with a homogeneous dropout definition in terms of not completing 100% of the program had a higher between-study heterogeneity than defining dropout as something different. This suggests a need for not only a consistent approach to dropout definition but also a more intentional approach. Patient fidelity emerged as the most frequently reported implementation outcome, whereas therapist fidelity was seldom assessed. This contrasts with findings from traditional face-to-face psychotherapy research, where therapist adherence and competence are routinely evaluated [83]. Scholars suggest that researchers may perceive therapist fidelity playing a lesser role in treatment results due to the standardized format while highlighting the challenges associated with ensuring patient fidelity [84,85]. Findings from this review also indicate consistently low therapist time per patient per week relative to face-to-face care, suggesting a redistribution of therapeutic labor from direct clinician contact to structured, technology-mediated intervention components. In addition, stakeholders report parental support as central to motivation and engagement for the treatment. However, studies comparing e-therapy with and without therapist guidance show markedly better outcomes in therapist-guided versus self-help programs in terms of implementation and treatment, pointing to the importance of therapist support [86]. More research is needed on the role therapist fidelity plays in e-therapy, especially in the context of CAMHS.

Clinical research on e-therapy has grown substantially during the last decade with promising results. However, the findings related to implementation outcomes and determinants remain scarce. We found that few studies assessed the prevalence or significance of the reported barriers and facilitators for implementation synthesized in this review. Future research will benefit from using a consistent framework such as CFIR that could facilitate the generation of comparable data and enhance our understanding of implementation factors across various settings. Few studies included in this review reported implementation determinants prospectively, underscoring a significant gap in the existing literature. While retrospective applications of CFIR can furnish valuable insights into the effectiveness of implementation strategies, there is an increasing recognition of the need for frameworks to be employed prospectively to guide planning, execution, and evaluation in real time. Furthermore, no barriers or facilitators were identified at the process level, highlighting an important knowledge gap in the CFIR framework. Researchers are encouraged to quantitatively measure and report proportional data on implementation processes and outcomes using CFIR factors.

Continued research on implementation specifically in child and adolescent mental health services is needed, as findings from the adult mental health literature cannot be assumed to apply to all outcomes and barriers and facilitators. This also applies to CFIR barriers and facilitators that overlap in the context of adult and youth mental health, as the content of each factor may differ qualitatively at each level and may require specialized strategies. In addition, more research is needed on the role therapist fidelity plays in e-therapy, especially in the context of CAMHS. Furthermore, analyses are warranted to assess the different definitions of dropout of e-therapy in CAMHS. Interestingly, none of the included studies reported barriers or facilitators from the perspective of leadership or policymakers, leaving potentially important perspectives unexplored.

### Limitations

Interpretation of these findings is tempered by several limitations. The breadth of the review question introduced heterogeneity across study designs, populations, and intervention modalities. Most included studies originated from high-income Western countries, limiting generalizability to low- and middle-income contexts. Interventions were predominantly CBT-based and anxiety-focused, which may not represent the broader spectrum of digital therapies. Excluding studies on e-therapy in schools and child welfare services also gives reduced generalizability of our findings to these contexts. Measures of patient satisfaction often relied on nonvalidated instruments adapted from adult populations and were typically collected postintervention, potentially inflating acceptability estimates by excluding noncompleters [21]. Measures of fidelity also presented some risks of bias in terms of reporting, limiting comparability and interpretability across studies. There were not enough data to predict missing values for satisfaction level and satisfaction rate through multiple imputation. Additionally, the small number of eligible studies limited analyses of small study effects, moderator effects, prevalence of barriers and facilitators, and statistical relationships between the synthesized implementation determinants and outcomes. Given our broad research scope and objectives, the variability in study design, context, implementation measurement, and reporting was anticipated and arguably appropriately accounted for through the incorporation of random effects measurements in our meta-analysis [87].

### Conclusions

This systematic review provides an integrated understanding of what drives effective e-therapy implementation by presenting synthesized evidence on the implementation of therapist-supported e-therapy in CAMHS, with parallel analyses pooling key implementation benchmark estimates, and identifying common barriers and facilitators at four levels: the intervention, the organization, the therapist, and the patient. These findings highlight service-level enablers, such as leadership anchoring, targeted use, technical stability, structured patient flow, and therapist training, that organizations can prioritize to strengthen sustainable e-therapy implementation in CAMHS. While e-therapy shows promise in terms of cost-effectiveness and patient acceptability, variability in fidelity and limited prospective research highlight significant gaps. The results underscore the need for standardized reporting, prospective application of frameworks such as CFIR, inclusion of multiple stakeholder perspectives, and more rigorously designed studies to assess the importance of these factors to guide effective, context-specific implementation strategies and enhance mental health service delivery for young people. The pooled benchmarks and synthesized determinants presented here offer a foundation for optimizing implementation strategies and advancing equitable access to high-quality e-therapy within youth mental health services.

## Supplementary material

10.2196/83543 Multimedia Appendix 1 Search strategies for implementation of internet-delivered therapist-guided therapy in child and adolescent mental health services.

10.2196/83543 Multimedia Appendix 2 Supplementary detailed description of methods and review statistics.

10.2196/83543 Multimedia Appendix 3 Data Extraction.

10.2196/83543 Multimedia Appendix 4 Themes and quotes from qualitative synthesis.

10.2196/83543 Multimedia Appendix 5 Prior specifications.

10.2196/83543 Multimedia Appendix 6 Model diagnostics of convergence.

10.2196/83543 Multimedia Appendix 7 Summary of risk of bias assessments.

10.2196/83543 Multimedia Appendix 8 Forest and funnel plots.

10.2196/83543 Multimedia Appendix 9 Sensitivity analyses.

10.2196/83543 Checklist 1 PRISMA checklist.

## Abbreviations

CAMHS: child and adolescent mental health services
CASP: Critical Appraisal Skills Programme
CBT: cognitive behavioral therapy
CFIR: Consolidated Framework of Implementation Research
CI: credible interval
e-therapy: internet-delivered therapist-guided therapy
PRISMA-S: Preferred Reporting Items for Systematic Review and Meta-Analyses literature search extension
RCT: randomized controlled trial
