# Supplementary material for: Barriers to and Facilitators of Implementation of Internet-Delivered Therapist-Guided Therapy in Child and Adolescent Mental Health Services: Systematic Review and Bayesian Meta-Analysis
Source: J Med Internet Res. 2025 Dec 22;27:e83543. doi: 10.2196/83543 (PMC12721491; doi:10.2196/83543)
Supplement: Multimedia Appendix 1 [file jmir-v27-e83543-s001.docx]

Appendix 1 - Search strategies for implementation of internet-delivered therapist-guided therapy in child and adolescent mental health services

Date searched: 6 June 2025 (initial search 31 January 2024)

| **Database** | **Number of retrieved records** |
| --- | --- |
| PsycInfo (Ovid) | 9 070 |
| Medline (Ovid) | 8 872 |
| Embase (Ovid) | 15 800 |
| Cinahl (Ebsco) | 3 064 |
| Cochrane Central Register of Controlled Trials (CENTRAL) (Wiley) | 4 229 |
| Web of Science (Clarivate) | 7 602 |
| ProQuest Dissertations & Thesis | 1 389 |
| Total number of hits: | 50 026 |
| Total number of hits after removing duplicates: | 32 171 |

*The search in ProQuest Dissertations & Thesis was only run in 2024, due to no longer access in 2025.*

Database:  **APA PsycInfo (Ovid)**1806 to May 2025 Week 4
Date: 6 June 2025

Hits: 9070

| **#** | **Searches** | **Results** |
| --- | --- | --- |
| 1 | mental health services/ or community mental health services/ or mental health programs/ or psychological first aid/ or health care services/ or behavioral health services/ or primary health care/ or health care delivery/ or public health service/ or mental health/ or community health/ or behavioral health/ or psychiatry/ or community psychiatry/ or consultation liaison psychiatry/ or neuropsychiatry/ or social psychiatry/ or clinics/ or child guidance clinics/ or psychiatric clinics/ or walk in clinics/ or community mental health centers/ or outpatients/ or outpatient treatment/ | 307162 |
| 2 | (mental health* or psychological health* or psychiatr* or neuropsychiatr* or (primary adj3 care*) or psychological first aid* or healthcare* or health care* or (community adj1 mental) or (counsel?ing adj4 service*) or outpatient* or out-patient* or child guidance clinic* or student-run clinic* or camhs* or cap).ti,ab,id. | 778028 |
| 3 | 1 or 2 | 828754 |
| 4 | pediatrics/ or puberty/ or ("childhood birth 12 yrs" or "adolescence 13 17 yrs" or "young adulthood 18 29 yrs").ag. | 1481814 |
| 5 | (child* or kid or kids* or minors* or adolescen* or teen* or preteen* or tween* or juvenil* or young adult* or youth or youngster or young people* or puberty* or prepuberty* or girl* or boy or boys* or pediatric* or peadiatric*).ti,ab,id. | 1184941 |
| 6 | 4 or 5 | 1901893 |
| 7 | 3 and 6 | 296463 |
| 8 | youth mental health/ or child psychiatry/ or adolescent psychiatry/ or exp childhood development/ or exp adolescent development/ | 215087 |
| 9 | or/7-8 | 474898 |
| 10 | evidence based practice/ or knowledge transfer/ | 26750 |
| 11 | (implement* or ((evidence-based or evidencebased) adj2 practice*) or integrat* or application* or adapt* or develop* or disseminat* or (knowledge adj3 (translat* or transfer*)) or translational* or initiat*).ti,ab,id. | 2072195 |
| 12 | or/10-11 | 2077748 |
| 13 | exp cognitive behavior therapy/ or exp behavior therapy/ or psychotherapy/ or psychotherapeutic techniques/ or psychotherapeutic processes/ or psychotherapeutic counseling/ or counseling/ or treatment/ or preventive mental health services/ | 343972 |
| 14 | (((cognitive or behavi* or prevent* or mental* or psych*) adj3 (therap* or treat* or intervention* or program* or strateg* or training*)) or psychotherap* or counsel?ing* or cbt or icbt or ((mental* or psych*) adj10 intervention*)).ti,ab,id. | 614835 |
| 15 | exp mental disorders/ or exp major depression/ or exp anxiety/ or exp anxiety disorders/ or exp obsessive compulsive disorder/ or exp posttraumatic stress disorder/ or exp autism spectrum disorders/ or attention deficit disorder/ or attention deficit disorder with hyperactivity/ | 1176604 |
| 16 | (((mental* or psych*) adj3 (disorder* or disease*)) or anxiet* or depression* or depressiv* or obsessive-compulsiv* or ocd or posttrauma* or post-trauma* or ptsd* or autis* or attention deficit* or adhd).ti,ab,id. | 788070 |
| 17 | or/13-16 | 1845396 |
| 18 | computer assisted therapy/ or exp telemedicine/ or internet/ or digital technology/ or mobile technology/ or electronic health services/ or digital interventions/ or mobile health/ or computers/ or exp mobile devices/ or computer applications/ or mobile applications/ or computer software/ | 100160 |
| 19 | (internet* or online* or computer* or digital* or telemedic* or tele-medic* or telehealth* or tele-health* or teletherap* or tele-therap* or telecounsel* or tele-counsel* or teleconsultation* or tele-consultation* or teleconference* or tele-conferenc* or telepsych* or tele-psych* or software* or smartphone* or smart phone* or cell phone* or mobile phone* or mobile device* or mobile appl* or mobile health* or mobile technolog* or tablet computer* or ehealth* or e-health* or e-therap* or etherap* or e-mental health* or emental health* or e-aid or eaid or e-counsel?ing or ecounsel?ing or cybercounsel?ing or cyber-counsel?ing).ti,ab,id. | 368771 |
| 20 | web*.ti,id. | 17869 |
| 21 | (web* not "web of science").ab. | 59766 |
| 22 | "web of science".ab. and web*.ab. /freq=2 | 973 |
| 23 | or/18-22 | 415992 |
| 24 | 9 and 12 and 17 and 23 | 9875 |
| 25 | "systematic review"/ or "literature review"/ or meta analysis/ | 29141 |
| 26 | ("literature review" or "systematic review" or "meta analysis").md. or review*.dt. or (systematic review or scoping review or literature review or meta-analys* or metaanalys*).ti. | 371592 |
| 27 | or/25-26 | 373286 |
| 28 | 24 not 27 | 9070 |

Database:  **MEDLINE ALL (Ovid)**1946 to June 05, 2025

Date: 6 June 2025

Hits: 8872

| **#** | **Searches** | **Results** |
| --- | --- | --- |
| 1 | Mental health services/ or Community mental health services/ or Community mental health centers/ or Community health services/ or Community health centers/ or Psychological first aid/ or Health services/ or Primary health care/ or "Delivery of health care"/ or Mental health/ or Psychiatry/ or Community psychiatry/ or Neuropsychiatry/ or Outpatients/ or Outpatient clinics, hospital/ or Child guidance clinics/ | 473825 |
| 2 | (mental health* or psychological health* or psychiatr* or neuropsychiatr* or (primary adj3 care*) or psychological first aid* or healthcare* or health care* or (community adj1 mental) or (counsel?ing adj4 service*) or outpatient* or out-patient* or child guidance clinic* or student-run clinic* or camhs* or cap).ti,ab,kf. | 1807017 |
| 3 | 1 or 2 | 1959898 |
| 4 | Pediatrics/ or Child/ or Adolescent/ or Young adult/ or Puberty/ | 3861854 |
| 5 | (child* or kid or kids* or minors* or adolescen* or teen* or preteen* or tween* or juvenil* or young adult* or youth or youngster or young people* or puberty* or prepuberty* or girl* or boy or boys* or pediatric* or peadiatric*).ti,ab,kf. | 2618144 |
| 6 | 4 or 5 | 4873419 |
| 7 | 3 and 6 | 464631 |
| 8 | Child Psychiatry/ or Adolescent Psychiatry/ or Child Health Services/ or Adolescent Health Services/ or Child health/ or Adolescent health/ or Child development/ or Adolescent development/ or Child behavior/ or Adolescent behavior/ | 145631 |
| 9 | or/7-8 | 581631 |
| 10 | Health plan implementation/ or Evidence-Based Practice/ or Translational Science, Biomedical/ | 19784 |
| 11 | (implement* or ((evidence-based or evidencebased) adj2 practice*) or integrat* or application* or adapt* or develop* or disseminat* or (knowledge adj3 (translat* or transfer*)) or translational* or initiat*).ti,ab,kf. | 9662274 |
| 12 | or/10-11 | 9667579 |
| 13 | exp Cognitive Behavioral Therapy/ or exp Behavior therapy/ or Psychotherapy/ or Psychotherapeutic Processes/ or Counseling/ or Therapeutics/ | 200679 |
| 14 | (((cognitive or behavi* or prevent* or mental* or psych*) adj3 (therap* or treat* or intervention* or program* or strateg* or training*)) or psychotherap* or counsel?ing* or cbt or icbt or ((mental* or psych*) adj10 intervention*)).ti,ab,kf. | 873795 |
| 15 | exp Mental disorders/ or Depression/ or exp Depressive disorder/ or exp Anxiety/ or exp Anxiety disorders/ or exp Obsessive-compulsive disorder/ or Stress Disorders, Post-Traumatic/ or exp Autism spectrum disorder/ or "Attention deficit and disruptive behavior disorders"/ or Attention deficit disorder with hyperactivity/ | 1713806 |
| 16 | (((mental* or psych*) adj3 (disorder* or disease*)) or anxiet* or depression* or depressiv* or obsessive-compulsiv* or ocd or posttrauma* or post-trauma* or ptsd* or autis* or attention deficit* or adhd).ti,ab,kf. | 1012022 |
| 17 | or/13-16 | 2824614 |
| 18 | Therapy, Computer-Assisted/ or Telemedicine/ or Internet/ or Internet-Based Intervention/ or Digital technology/ or Computers/ or Computers, handheld/ or Smartphone/ or Software/ or Mobile applications/ or Web browser/ | 325145 |
| 19 | (internet* or online* or computer* or digital* or telemedic* or tele-medic* or telehealth* or tele-health* or teletherap* or tele-therap* or telecounsel* or tele-counsel* or teleconsultation* or tele-consultation* or teleconference* or tele-conferenc* or telepsych* or tele-psych* or software* or smartphone* or smart phone* or cell phone* or mobile phone* or mobile device* or mobile app* or mobile health* or mobile technolog* or tablet computer* or ehealth* or e-health* or e-therap* or etherap* or e-mental health* or emental health* or e-aid or eaid or e-counsel?ing or ecounsel?ing or cybercounsel?ing or cyber-counsel?ing).ti,ab,kf. | 1240768 |
| 20 | web*.ti,kf. | 46896 |
| 21 | (web* not "web of science").ab. | 179096 |
| 22 | "web of science".ab. and web*.ab. /freq=2 | 4942 |
| 23 | or/18-22 | 1490316 |
| 24 | 9 and 12 and 17 and 23 | 10705 |
| 25 | (review or systematic review or meta-analysis or clinical trial protocol).pt. | 3711559 |
| 26 | (systematic review or scoping review or literature review or meta-analys* or metaanalys*).ti. | 493143 |
| 27 | or/25-26 | 3824922 |
| 28 | 24 not 27 | 8872 |

Database:  **Embase (Ovid)** 1974 to 2025 June 04

Date: 6 June 2025

Hits: 11118

| **#** | **Searches** | **Results** |
| --- | --- | --- |
| 1 | Mental health service/ or Community mental health service/ or Community mental health/ or Psychological first aid/ or Health service/ or Health care/ or Primary health care/ or Primary medical care/ or Health care delivery/ or Public health service/ or Mental health/ or Psychiatry/ or Liaison psychiatry/ or Neuropsychiatry/ or Social psychiatry/ or Outpatient department/ or Outpatients/ or Outpatient care/ or Community mental health center/ or Mental health center/ or Student-run clinic/ | 1432432 |
| 2 | (mental health* or psychological health* or psychiatr* or neuropsychiatr* or (primary adj3 care*) or psychological first aid* or healthcare* or health care* or (community adj1 mental) or (counsel?ing adj4 service*) or outpatient* or out-patient* or child guidance clinic* or student-run clinic* or camhs* or cap).ti,ab,kf. | 2502387 |
| 3 | 1 or 2 | 3032139 |
| 4 | Pediatrics/ or Child/ or Childhood/ or Juvenile/ or Adolescence/ or Puberty/ or Prepuberty/ or Young adult/ | 3043358 |
| 5 | (child* or kid or kids* or minors* or adolescen* or teen* or preteen* or tween* or juvenil* or young adult* or youth or youngster or young people* or puberty* or prepuberty* or girl* or boy or boys* or pediatric* or peadiatric*).ti,ab,kf. | 3350355 |
| 6 | 4 or 5 | 4444731 |
| 7 | 3 and 6 | 561226 |
| 8 | Child psychiatry/ or Child health care/ or Child health/ or Adolescent health/ or Child development/ or Adolescent development/ or Child behavior/ or Adolescent behavior/ | 219478 |
| 9 | or/7-8 | 721741 |
| 10 | Implementation science/ or Evidence based practice/ or Translational science/ or Translational research/ | 141971 |
| 11 | (implement* or ((evidence-based or evidencebased) adj2 practice*) or integrat* or application* or adapt* or develop* or disseminat* or (knowledge adj3 (translat* or transfer*)) or translational* or initiat*).ti,ab,kf. | 12088043 |
| 12 | or/10-11 | 12146654 |
| 13 | exp Cognitive behavioral therapy/ or exp Behavior therapy/ or Psychotherapy/ or Counseling/ or Therapy/ or Psychiatric treatment/ | 1856676 |
| 14 | (((cognitive or behavi* or prevent* or mental* or psych*) adj3 (therap* or treat* or intervention* or program* or strateg* or training*)) or psychotherap* or counsel?ing* or cbt or icbt or ((mental* or psych*) adj10 intervention*)).ti,ab,kf. | 1186926 |
| 15 | exp mental disease/ or depression/ or exp anxiety/ or exp anxiety disorders/ or exp obsessive compulsive disorder/ or posttraumatic stress disorder/ or exp autism/ or attention deficit hyperactivity disorder/ | 3225120 |
| 16 | (((mental* or psych*) adj3 (disorder* or disease*)) or anxiet* or depression* or depressiv* or obsessive-compulsiv* or ocd or posttrauma* or post-trauma* or ptsd* or autis* or attention deficit* or adhd).ti,ab,kf. | 1371903 |
| 17 | or/13-16 | 5752305 |
| 18 | Computer assisted therapy/ or Telehealth/ or Telemedicine/ or Internet/ or Web-Based Intervention/ or Web browser/ or Computer/ or Online system/ or Software/ or Mobile application/ or Mobile health application/ or Telepsychiatry/ or e-counseling/ or Teleconsultation/ | 662322 |
| 19 | (internet* or online* or computer* or digital* or telemedic* or tele-medic* or telehealth* or tele-health* or teletherap* or tele-therap* or telecounsel* or tele-counsel* or teleconsultation* or tele-consultation* or teleconference* or tele-conferenc* or telepsych* or tele-psych* or software* or smartphone* or smart phone* or cell phone* or mobile phone* or mobile device* or mobile app* or mobile health* or mobile technolog* or tablet computer* or ehealth* or e-health* or e-therap* or etherap* or e-mental health* or emental health* or e-aid or eaid or e-counsel?ing or ecounsel?ing or cybercounsel?ing or cyber-counsel?ing).ti,ab,kf. | 1687401 |
| 20 | web*.ti,kf. | 53419 |
| 21 | (web* not "web of science").ab. | 240757 |
| 22 | "web of science".ab. and web*.ab. /freq=2 | 4202 |
| 23 | or/18-22 | 2030560 |
| 24 | 9 and 12 and 17 and 23 | 18123 |
| 25 | "review"/ or "systematic review"/ or meta analysis/ or clinical protocol/ or clinical trial protocol/ | 3685627 |
| 26 | (systematic review or scoping review or literature review or meta-analys* or metaanalys*).ti. | 571395 |
| 27 | or/25-26 | 3800717 |
| 28 | 24 not 27 | 15800 |

Database:  **Cinahl (EBSCOhost)**

Date: 6 June 2025

Hits: 3064

| # | Query | Results |
| --- | --- | --- |
| S1 | (MH "Mental Health Services") OR (MH "Community Mental Health Services") OR (MH "Community Health Centers") OR (MH "Psychological First Aid") OR (MH "Health Services") OR (MH "Primary Health Care") OR (MH "Health Care Delivery") OR (MH "Mental Health") OR (MH "Psychiatry") OR (MH "Outpatients") OR (MH "Outpatient Service") | 336,396 |
| S2 | TI ("mental health*" or "psychological health*" or psychiatr* or neuropsychiatr* or (primary N2 care*) or "psychological first aid*" or healthcare* or "health care*" or (community N0 mental) or (counsel#ing N3 service*) or outpatient* or out-patient* or "child guidance clinic*" or "student-run clinic*” or camhs* or cap) OR AB (("mental health*" or "psychological health*" or psychiatr* or neuropsychiatr* or (primary N2 care*) or "psychological first aid*" or healthcare* or "health care*" or (community N0 mental) or (counsel#ing N3 service*) or outpatient* or out-patient* or "child guidance clinic*" or "student-run clinic*” or camhs* or cap) | 779,295 |
| S3 | S1 OR S2 | 924,455 |
| S4 | (MH "Pediatrics") OR (MH "Child") OR (MH "Adolescence") OR (MH "Young Adult") OR (MH "Puberty") | 1,074,900 |
| S5 | TI ( child* or kid or kids* or minors* or adolescen* or teen* or preteen* or tween* or juvenil* or "young adult*" or youth or youngster or "young people*" or puberty* or prepuberty* or girl* or boy or boys* or pediatric* or peadiatric* ) OR AB ( child* or kid or kids* or minors* or adolescen* or teen* or preteen* or tween* or juvenil* or "young adult*" or youth or youngster or "young people*" or puberty* or prepuberty* or girl* or boy or boys* or pediatric* or peadiatric* ) | 917,384 |
| S6 | S4 OR S5 | 1,447,496 |
| S7 | S3 AND S6 | 199,476 |
| S8 | (MH "Child Psychiatry") OR (MH "Adolescent Psychiatry") OR (MH "Child health services") OR (MH "Adolescent Health Services ") OR (MH "Child health") OR (MH "Adolescent Health") | 38,413 |
| S9 | S7 OR S8 | 227,455 |
| S10 | (MH "Implementation Science") OR (MH "Professional Practice, Evidence-Based") OR (MH "Translational Medical Research") | 29,800 |
| S11 | TI ( implement* or (("evidence-based" or evidencebased) N1 practice*) or integrat* or application* or adapt* or develop* or disseminat* or (knowledge N2 (translat* or transfer*)) or translational* or initiat* ) OR AB ( implement* or (("evidence-based" or evidencebased) N1 practice*) or integrat* or application* or adapt* or develop* or disseminat* or (knowledge N2 (translat* or transfer*)) or translational* or initiat* ) | 1,604,228 |
| S12 | S10 OR S11 | 1,620,234 |
| S13 | (MH " Cognitive therapy+") OR (MH "Behavior Therapy+") OR (MH "Psychotherapy") OR (MH "Counseling") OR (MH "Psychotherapeutic Processes") OR (MH "Therapeutics") | 107,107 |
| S14 | TI ( ((cognitive or behavi* or prevent* or mental* or psych*) N2 (therap* or treat* or intervention* or program* or strateg* or training*)) or psychotherap* or counsel#ing* or cbt or icbt or ((mental* or psych*) N9 intervention*)) ) OR AB ( ((cognitive or behavi* or prevent* or mental* or psych*) N2 (therap* or treat* or intervention* or program* or strateg* or training*)) or psychotherap* or counsel#ing* or cbt or icbt or ((mental* or psych*) N9 intervention*)) ) | 306,043 |
| S15 | (MH "Mental Disorders+") OR (MH "Depression") OR (MH "Anxiety+") OR (MH "Anxiety Disorders+") OR (MH "Obsessive-Compulsive Disorder+") OR (MH "Stress Disorders, Post-Traumatic") OR (MH "Autistic Disorder") OR (MH "Attention Deficit Hyperactivity Disorder") | 719,171 |
| S16 | TI ( ((mental* or psych*) N2 (disorder* or disease*)) or anxiet* or depression* or depressiv* or "obsessive-compulsiv*" or ocd or posttrauma* or "post-trauma*" or ptsd* or autis* or "attention deficit*" or adhd ) OR TI ( ((mental* or psych*) N2 (disorder* or disease*)) or anxiet* or depression* or depressiv* or "obsessive-compulsiv*" or ocd or posttrauma* or "post-trauma*" or ptsd* or autis* or "attention deficit*" or adhd ) | 180,502 |
| S17 | S13 OR S14 OR S15 OR S16 | 1,012,111 |
| S18 | (MH "Therapy, Computer Assisted") OR (MH "Internet") OR (MH "Internet-Based Intervention") OR (MH "Digital Technology") OR (MH "Computers and Computerization") OR (MH "Computers, Portable") OR (MH "Computers, Hand-Held") OR (MH "Smartphone") OR (MH "Mobile Applications") OR (MH "Software") OR (MH "Online Systems") OR (MH "Telehealth") OR (MH "Telemedicine") OR (MH "Telepsychiatry") | 157,868 |
| S19 | TI ( internet* or web* or online* or computer* or digital* or telemedic* or "tele-medic*" or telehealth* or "tele-health*" or teletherap* or "tele-therap*" or telecounsel* or "tele-counsel*" or teleconsultation* or "tele-consultation*" or teleconference* or "tele-conferenc*" or telepsych* or "tele-psych*" or software* or smartphone* or "smart phone*" or "cell phone*" or "mobile phone*" or "mobile device*" or "mobile app*" or "mobile health*" or "mobile technolog*" or "tablet computer*" or ehealth* or "e-health*" or "e-therap*" or etherap* or "e-mental health*" or "emental health*" or "e-aid" or eaid or "e-counsel#ing" or ecounsel?ing or cybercounsel?ing or "cyber-counsel#ing" ) OR AB ( internet* or online* or computer* or digital* or telemedic* or "tele-medic*" or telehealth* or "tele-health*" or teletherap* or "tele-therap*" or telecounsel* or "tele-counsel*" or teleconsultation* or "tele-consultation*" or teleconference* or "tele-conferenc*" or telepsych* or "tele-psych*" or software* or smartphone* or "smart phone*" or "cell phone*" or "mobile phone*" or "mobile device*" or "mobile app*" or "mobile health*" or "mobile technolog*" or "tablet computer*" or ehealth* or "e-health*" or "e-therap*" or etherap* or "e-mental health*" or "emental health*" or "e-aid" or eaid or "e-counsel#ing" or ecounsel?ing or cybercounsel?ing or "cyber-counsel#ing" ) | 373,843 |
| S20 | S18 OR S19 | 445,126 |
| S21 | S9 AND S12 AND S17 AND S20 | 3,498 |
| S22 | S9 AND S12 AND S17 AND S20  Limiters - Publication Type: Book Review, Meta Analysis, Protocol, Review, Systematic Review Search modes - Boolean/Phrase | 434 |
| S23 | S21 NOT S22 | 3,064 |

Database:  **Cochrane Central Register of Controlled Trials (CENTRAL) (Wiley)**

Date: 6 June 2025

Hits: 4229

| ID | Search | Hits |
| --- | --- | --- |
| #1 | [mh ^"Mental health services"] or [mh ^"Community mental health services"] or [mh ^"Community mental health centers"] or [mh ^"Community health services"] or [mh ^"Community health centers"] or [mh ^"Psychological first aid"] or [mh ^"Health services"] or [mh ^"Primary health care"] or [mh ^"Delivery of health care"] or [mh ^"Mental health"] or [mh ^"Psychiatry"] or [mh ^"Community psychiatry"] or [mh ^"Neuropsychiatry"] or [mh ^"Outpatients"] or [mh ^"Outpatient clinics, hospital"] or [mh ^"Child guidance clinics"] | 17703 |
| #2 | ((mental NEXT health*) or (psychological NEXT health*) or psychiatr* or neuropsychiatr* or (primary NEAR/2 care*) or (psychological NEXT first NEXT aid*) or healthcare* or (health NEXT care*) or (community NEAR/0 mental) or (counsel?ing NEAR/3 service*) or outpatient* or out-patient* or (child NEXT guidance NEXT clinic*) or (student-run NEXT clinic*) or camhs* or cap):ti,ab,kw | 233781 |
| #3 | #1 or #2 | 234675 |
| #4 | [mh ^"Pediatrics"] or [mh ^"Child"] or [mh ^"Adolescent"] or [mh ^"Young adult"] or [mh ^"Puberty"] | 222857 |
| #5 | (child* or kid or kids* or minors* or adolescen* or teen* or preteen* or tween* or juvenil* or (young NEXT adult*) or youth or youngster or (young NEXT people*) or puberty* or prepuberty* or girl* or boy or boys* or pediatric* or peadiatric*):ti,ab,kw | 409891 |
| #6 | #4 or #5 | 409891 |
| #7 | #3 and #6 | 56872 |
| #8 | [mh ^"Child Psychiatry"] or [mh ^"Adolescent Psychiatry"] or [mh ^"Child Health Services"] or [mh ^"Adolescent Health Services"] or [mh ^"Child health"] or [mh ^"Adolescent health"] or [mh ^"Child development"] or [mh ^"Adolescent development"] or [mh ^"Child behavior"] or [mh ^"Adolescent behavior"] | 7135 |
| #9 | [1-#8] | 62246 |
| #10 | [mh ^"Health plan implementation"] or [mh ^"Evidence-Based Practice"] or [mh ^"Translational Science, Biomedical"] | 786 |
| #11 | (implement* or ((evidence-based or evidencebased) NEAR/1 practice*) or integrat* or application* or adapt* or develop* or disseminat* or (knowledge NEAR/2 (translat* or transfer*)) or translational* or initiat*):ti,ab,kw | 484616 |
| #12 | [2-#11] | 484616 |
| #13 | [mh "Cognitive Behavioral Therapy"] or [mh "Behavior therapy"] or [mh ^"Psychotherapy"] or [mh ^"Psychotherapeutic Processes"] or [mh ^"Counseling"] or [mh ^"Therapeutics"] | 33806 |
| #14 | (((cognitive or behavi* or prevent* or mental* or psych*) NEAR/2 (therap* or treat* or intervention* or program* or strateg* or training*)) or psychotherap* or counsel?ing* or cbt or icbt or ((mental* or psych*) NEAR/9 intervention*)):ti,ab,kw | 207383 |
| #15 | [mh "Mental disorders"] or [mh ^"Depression"] or [mh "Depressive disorder"] or [mh "Anxiety"] or [mh "Anxiety disorders"] or [mh "Obsessive-compulsive disorder"] or [mh ^"Stress Disorders, Post-Traumatic"] or [mh "Autism spectrum disorder"] or [mh ^"Attention deficit and disruptive behavior disorders"] or [mh ^"Attention deficit disorder with hyperactivity"] | 127293 |
| #16 | (((mental* or psych*) NEAR/2 (disorder* or disease*)) or anxiet* or depression* or depressiv* or obsessive-compulsiv* or ocd or posttrauma* or post-trauma* or ptsd* or autis* or (attention NEXT deficit*) or adhd):ti,ab,kw | 202416 |
| #17 | [3-#16] | 375535 |
| #18 | [mh ^"Therapy, Computer-Assisted"] or [mh ^"Telemedicine"] or [mh ^"Internet"] or [mh ^"Internet-Based Intervention"] or [mh ^"Digital technology"] or [mh ^"Computers"] or [mh ^"Computers, handheld"] or [mh ^"Smartphone"] or [mh ^"Software"] or [mh ^"Mobile applications"] or [mh ^"Web browser"] | 15902 |
| #19 | (internet* or web* or online* or computer* or digital* or telemedic* or tele-medic* or telehealth* or tele-health* or teletherap* or tele-therap* or telecounsel* or tele-counsel* or teleconsultation* or tele-consultation* or teleconference* or tele-conferenc* or telepsych* or tele-psych* or software* or smartphone* or (smart NEXT phone*) or (cell NEXT phone*) or (mobile NEXT phone*) or (mobile NEXT device*) or (mobile NEXT app*) or (mobile NEXT health*) or (mobile NEXT technolog*) or (tablet NEXT computer*) or ehealth* or e-health* or e-therap* or etherap* or (e-mental NEXT health*) or (emental NEXT health*) or e-aid or eaid or e-counsel?ing or ecounsel?ing or cybercounsel?ing or cyber-counsel?ing):ti,ab,kw | 188547 |
| #20 | [4-#19] | 188547 |
| #21 | #9 and #12 and #17 and #20 | 4340 |
| #22 | #21 in Trials | 4235 |
| #23 | ("systematic review" or "scoping review" or "literature review" or meta-analys* or metaanalys*):ti | 7678 |
| #24 | #22 NOT #23 | 4229 |

Database:  **Web of Science Core Collection (Clarivate)**

Science Citation Index Expanded 1945-present, Social Sciences Citation Index 1956-present,

Arts & Humanities Citation Index 1975-present, Emerging Sources Citation Index 2019-present.

Date: 6 June 2025

Hits: 7602

| # | Search Query | Results |
| --- | --- | --- |
| 1 | TS=("mental health*" or "psychological health*" or psychiatr* or neuropsychiatr* or (primary NEAR/2 care*) or "psychological first aid*" or healthcare* or "health care*" or (community NEAR/0 mental) or (counsel?ing NEAR/3 service*) or outpatient* or "out-patient*" or "child guidance clinic*" or "student-run clinic*" or camhs* or cap) | 2,067,901 |
| 2 | TS=(child* or kid or kids* or minors* or adolescen* or teen* or preteen* or tween* or juvenil* or "young adult*" or youth or youngster or "young people*" or puberty* or prepuberty* or girl* or boy or boys* or pediatric* or peadiatric*) | 3,533,721 |
| 3 | TS=(implement* or (("evidence-based" or evidencebased) NEAR/1 practice*) or integrat* or application* or adapt* or develop* or disseminat* or (knowledge NEAR/2 (translat* or transfer*)) or translational* or initiat*) | 18,493,075 |
| 4 | TS=(((cognitive or behavi* or prevent* or mental* or psych*) NEAR/2 (therap* or treat* or intervention* or program* or strateg* or training*)) or psychotherap* or counsel?ing* or cbt or icbt or ((mental* or psych*) NEAR/9 intervention*)) | 872,817 |
| 5 | TS=(((mental* or psych*) NEAR/2 (disorder* or disease*)) or anxiet* or depression* or depressiv* or "obsessive-compulsiv*" or ocd or posttrauma* or "post-trauma*" or ptsd* or autis* or "attention deficit*" or adhd) | 1,395,695 |
| 6 | #4 OR #5 | 2,076,122 |
| 7 | TS=(internet* or web* or online* or computer* or digital* or telemedic* or "tele-medic*" or telehealth* or "tele-health*" or teletherap* or "tele-therap*" or telecounsel* or "tele-counsel*" or teleconsultation* or "tele-consultation*" or teleconference* or "tele-conferenc*" or telepsych* or "tele-psych*" or software* or smartphone* or "smart phone*" or "cell phone*" or "mobile phone*" or "mobile device*" or "mobile app*" or "mobile health*" or "mobile technolog*" or "tablet computer*" or ehealth* or "e-health*" or "e-therap*" or etherap* or "e-mental health*" or "emental health*" or "e-aid" or eaid or "e-counsel?ing" or ecounsel?ing or cybercounsel?ing or "cyber-counsel?ing") | 3,347,692 |
| 8 | #1 AND #2 AND #3 AND #6 AND #7 | 9,716 |
| 9 | (#1 AND #2 AND #3 AND #6 AND #7) NOT (DT==("REVIEW")) | 7,816 |
| 10 | TI=(systematic review or scoping review or literature review or meta-analys* or metaanalys*) | 683,171 |
| 11 | #9 NOT #10 | 7,602 |

Database:  **ProQuest Dissertations & Thesis Citation Index (Clarivate)**

Date: 31 January 2024 *(search not updated due to no access in 2025)*

Hits: 1389

| # | Search Query | Results |
| --- | --- | --- |
| 1 | TS=("mental health*" or "psychological health*" or psychiatr* or neuropsychiatr* or (primary NEAR/2 care*) or "psychological first aid*" or healthcare* or "health care*" or (community NEAR/0 mental) or (counsel?ing NEAR/3 service*) or outpatient* or "out-patient*" or "child guidance clinic*" or "student-run clinic*" or camhs* or cap) | 209841 |
| 2 | TS=(child* or kid or kids* or minors* or adolescen* or teen* or preteen* or tween* or juvenil* or young adult* or youth or youngster or young people* or puberty* or prepuberty* or girl* or boy or boys* or pediatric* or peadiatric*) | 439325 |
| 3 | TS=(implement* or (("evidence-based" or evidencebased) NEAR/1 practice*) or integrat* or application* or adapt* or develop* or disseminat* or (knowledge NEAR/2 (translat* or transfer*)) or translational* or initiat*) | 2612815 |
| 4 | TS=(((cognitive or behavi* or prevent* or mental* or psych*) NEAR/2 (therap* or treat* or intervention* or program* or strateg* or training*)) or psychotherap* or counsel?ing* or cbt or icbt or ((mental* or psych*) NEAR/9 intervention*)) | 183056 |
| 5 | TS=(((mental* or psych*) NEAR/2 (disorder* or disease*)) or anxiet* or depression* or depressiv* or "obsessive-compulsiv*" or ocd or posttrauma* or "post-trauma*" or ptsd* or autis* or "attention deficit*" or adhd) | 147364 |
| 6 | #4 OR #5 | 290657 |
| 7 | TS=(internet* or web* or online* or computer* or digital* or telemedic* or "tele-medic*" or telehealth* or "tele-health*" or teletherap* or "tele-therap*" or telecounsel* or "tele-counsel*" or teleconsultation* or "tele-consultation*" or teleconference* or "tele-conferenc*" or telepsych* or "tele-psych*" or software* or smartphone* or "smart phone*" or "cell phone*" or "mobile phone*" or "mobile device*" or "mobile app*" or "mobile health*" or "mobile technolog*" or "tablet computer*" or ehealth* or "e-health*" or "e-therap*" or etherap* or "e-mental health*" or "emental health*" or "e-aid" or eaid or "e-counsel?ing" or ecounsel?ing or cybercounsel?ing or "cyber-counsel?ing") | 647851 |
| 8 | #1 AND #2 AND #3 AND #6 AND #7 | 1389 |
