# Supplementary material for: Barriers to and Facilitators of Implementation of Internet-Delivered Therapist-Guided Therapy in Child and Adolescent Mental Health Services: Systematic Review and Bayesian Meta-Analysis
Source: J Med Internet Res. 2025 Dec 22;27:e83543. doi: 10.2196/83543 (PMC12721491; doi:10.2196/83543)
Supplement: Multimedia Appendix 2 [file jmir-v27-e83543-s002.docx]

Appendix 2 - Supplementary detailed description of methods and review statistics

*Screening*

ASReview version 1.6.6 was applied for the screening of abstracts and titles of search results. Adopting the SAFE procedure, two independent researchers: 1) Screened a random set of 1% of the total number of abstracts, providing training data for the AI-tool, 2) Applied the training data from step one in addition to ten target articles, to build a simple active learning model for sorting the remaining abstracts by relevancy (with Naïve Bayes as classifier and TD-IDF as feature extractor), 3) Screened the most relevant abstracts until both reviewers had a) rejected 50 abstracts in a row, and b) screened a minimum of 5% of the total dataset. During this phase the two reviewers stopped to discuss disagreements and updated the sorting model after 10, 40, 50 and then after approximately every 200 abstracts. 4) Screened the remaining abstracts using a deep learning model from the SAFE procedure (neural network as a classifier and sBert as a feature extractor) until 50 consecutive abstracts were excluded; 5) Evaluated and strengthened the quality of the AI-assisted screening process by reviewing a randomly selected 1% and the 10 highest and lowest ranked abstracts that were not screened, and making sure that all screened articles had been reviewed by two researchers.

*Data extraction*

Implementation outcome data were extracted for quantitative statistical synthesis only, as a synthesis of qualitative descriptions of stakeholders’ experiences of fidelity, acceptability and cost-effectiveness falls outside of the scope of this review. The included studies defined therapy dropout inconsistently, and the studies’ differently reported definition of dropout were extracted. Where a study reported several measures of dropout, the following priority order was used for extraction for pooled dropout estimates: self-report/active withdrawal, failure to complete arbitrary cut-offs for completion, failure to complete 100% of modules, and lost to follow-up. Only data regarding stakeholders reported experienced facilitators and barriers to implementation were extracted, not expected or suggested barriers and facilitators during intervention development without experience with the intervention implemented.

*Quality assessment procedures*

For the qualitative studies, we applied the Critical Appraisal Skills Program (CASP) Quality Research List [56]. This checklist assesses whether 1) the research aims are clearly stated; 2) qualitative methodology, research design, recruitment strategy, and data collection methods are appropriate; 3) relationships between researchers and participants are adequately considered; 4) ethical issues are significantly addressed; 5) data analyses are sufficiently rigorous; 6) findings are clearly stated; and 7) research is valuable overall. For the quantitative studies, we used the Cochrane Risk of Bias tool for randomized controlled trials, and the ROBINS-I tool for non-randomized studies of interventions. In mixed methods studies, the choice of quality assessment tools was tailored to the specific facilitators. Because the available data did not suffice to analyse the relationship between facilitating and hindering factors for implementation and implementation outcomes, the assessment constructs regarding group differences and confounding factors were not applicable from RoB2 (Domain 1-2) and ROBINS-I (Domain 1-4) [57]. Some concerns were found regarding the measurement and reporting of patient fidelity among three studies, and critical concerns regarding missing data in terms of stakeholder satisfaction among three studies. Most qualitative studies lacked an adequate consideration of potential researcher bias, and many lacked a sufficiently rigorous description and discussion of their qualitative data analysis.

*Bayesian meta-analysis procedures*

Random effects meta-analyses were run on the following implementation outcomes; therapy dropout, mean proportion of modules completed by patients, mean therapist time per patient per week, mean patient satisfaction rate and mean CSQ-8 patient satisfaction score. We conducted standard diagnostics for absence of convergence and checked for divergent transitions and found no obvious issues, indicating stable estimates of limits of credible intervals [58]. Effective sample sizes (estimates of the number of independent samples from the posterior distribution) were estimated for the bulk and tail of the distributions separately and were above 400 for all parameters [58]. The PSRF (potential scale deduction factor, R-hat) of all parameters were lower than 1.01. We refitted the models after conducting multiple imputation with the R-package ‘mice’ to create and analyze 20 multiple imputed datasets, using predictive mean matching as the imputation method and all extracted study variables as predictors for the imputation model [59-61]. All models were estimated using Hamiltonian Monte Carlo via the R-package *brms* [62]. We assessed the quality of our models through convergence plots and posterior predictive checks. Detailed results of these diagnostics can be found in Appendix 7. All models were estimated using Hamiltonian Monte Carlo via the R-package *brms* [62].

*Review statistics*

The reviewed studies were published between 2007 and 2025, representing global research from various sites; Sweden (*n* = 20, 40%), USA (*n* = 9, 18%), United Kingdom (*n* = 4, 8%), Australia (*n* 4, = 8%), Netherlands (*n* = 2, 4%), Canada (*n*= 2, 4%), Denmark (*n* = 2, 4%), Germany (*n* = 2, 4%), New Zealand (*n* = 1, 2%), Finland (*n* = 1, 2%), India (*n* = 1, 2%), Spain (*n* = 1, 2%), and one multinational study in Europe (2%). The reviewed studies used different research designs: RCT (*n* = 18, 36%), observational (*n* = 17, 34%), qualitative (*n* = 4, 8%) and mixed methods (*n* = 11, 22%). The included literature represented a range of mental health care contexts; primary (*n* = 11, 22%), secondary (*n* = 12, 24%), tertiary (*n* = 10, 20%), mixed (*n* = 3, 6%), research/university mental health clinics (*n* = 7, 14%) and seven studies did not report the site context (14%). The e-therapy programs used in the studies targeted a range of different mental disorders: anxiety (*n* = 24, 48%), depression (*n* = 9, 18%), transdiagnostic (*n* = 6, 12%), Tourette’s syndrome/ tics disorders (*n* = 3, 6%), sleep disorders (*n* = 2, 4%), autism spectrum disorder (*n* = 1, 2%), addiction (*n* = 1, 2%), conduct disorders (*n* = 1, 2%), well-being (*n* = 1, 2%), self-injury (*n* = 1, 2%), and chronic pain (*n* = 1, 2%). Most of the e-therapy programs were CBT-based (n=47, 94%), and two were based on family therapeutic interventions (n=2, 4%), and one was based on psychodynamic therapy (n=1, 2%). Most programs included parallel content for both patients and their parents (n= 31, 62%). Therapy programs varied in the amount and method of therapist support, using asynchronous chat (n=43, 86%), scheduled synchronous guidance online or face-to-face (n=28, 56%) and on demand phone calls, video or face-to-face sessions (17, 34%).
