# Supplementary material for: Barriers to and Facilitators of Implementation of Internet-Delivered Therapist-Guided Therapy in Child and Adolescent Mental Health Services: Systematic Review and Bayesian Meta-Analysis
Source: J Med Internet Res. 2025 Dec 22;27:e83543. doi: 10.2196/83543 (PMC12721491; doi:10.2196/83543)
Supplement: Multimedia Appendix 3 [file jmir-v27-e83543-s003.docx]

Appendix 3 - Data Extraction

Table 1. Study design, intervention features and implementation factors in the reviewed studies on internet-delivered, therapist-guided therapy in child and adolescent mental health services from 2007 to 2025

|  | **Country** | **Study design** | **Setting** | **Sample** | **Psychiatric Condition** | **Digital health intervention** | **Implementation Outcomes reported** | **Barrier/Facilitator investigated** |
| --- | --- | --- | --- | --- | --- | --- | --- | --- |
| Alvarez-Jiminez et al., 2025 [5] | Australia | Prospective cohort study with assessments at baseline, 6 months and 12 months | 93 youth mental health services, comprising 262 priamry and sexondary clinics | 5702 patients aged 12-21 years (mean: 17.5 years) | Depression, anxiety and sleep disorders | “MOST”, a CBT- based intervention with syncrounous support from a clinician and a peer worker and/or vocational worker. Improve Your Mood (Depression), Finding Your Calm (Anxiety), Improve Your Sleep (Sleep), Improve Your Confidence (Social Anxiety), and Social Hacks (Social Skills). Separate developmentally appropriate content is available for 12–14-year-olds. Young people are encouraged to complete their journey with asynchronous support from a clinician but can complete on their own. | Dropout rate (number of patients lost to follow-up after the baseline measure), mean number of login sessions, mean patient time over the first 12 weeks, proportion of patients using the program at 14 days, 12 weeks and 24 weeks. Patient rated satisfaction level and rate, and qualitative written feedback. | yes, post-treatment open survey questions |
| Andren et al., 2019 [6] | Sweden | Prospective RCT study with pre, post and 3, 6 and 12 month follow up assessments, comparing the feasibility of two online programs ("HRT" and "ERP”) in a therapist- and parent- guided format | Specialist health clinic | 23 patients aged 8-16 (BIP TIC ERC n=12 (age M=11.8, SD2.51), BIP TIC HRT n=11 (age M=12.79, SD= 2.62) | Tourette's disorder and tic disorder | "BIP TIC HRT" and "BIP TIC ERP", both 10-week internet-delivered CBT programmes with separate modules for patient and parents, with asynchronous daily therapist support via chat | Dropout rate (<100% intervention completion), mean number of modules completed, patient and parent reported treatment credibility, satisfaction and recommendation (on a scale of 1-5), therapist time per patient | no |
| Andren et al., 2024 [7] | Sweden | Prospective 12-month controlled follow-up of a single-masked, parallel group, superiority RCT comparing therapist-supported  internet-delivered ERP with therapist-supported internet-delivered education (comparator). | Research clinic with nationwide recruitment | 221 patients (n IG= 111, aged 12.1 (2.3) years) | Tics/Tourettes | “ORBIT”, a 10-module web-based CBT program with parallel parent modules, asynchronous therapist support via chat, and phone calls if necessary | Direct and indirect costs for treatment provided. Dropout rate (number of patients not completing the first 4 child chapters), mean number of completed modules, therapist time per patient per week, patient and parent rated credibility, satisfaction (CSQ-8) and therapist rated patient adherence | no |
| Aspvall et al., 2018 [8] | Sweden | Prospective cohort study with assessments pre-, post-treatment and 3-month follow-up. | Research unit at a secondary health clinic | 11 patients aged 8-11 (M=9.5, SD=1.0) | OCD | "BIP OCD Junior", 12 parallel child and parent modules of internet delivered CBT with asynchronous therapist support and calls if necessary | Dropout rate (lost to follow up), mean number of completed modules by patients and parents, parent and patient reported treatment satisfaction and credibility, therapist time per patient | no |
| Aspvall et al., 2021 [9] | Sweden | Prospective observational cohort study with assessments at baseline and posttreatment, comparing stepped-care with in-person CBT | 2 specialist OCD clinics | 152 patients aged 8-17 (M=13.4 , SD=2.5), (n IG = 74) | OCD | Guided internet-delivered CBT over 16 weeks consisting of 14 modules, parents have access to parallel modules, with asynchronous therapist support through chat, and additional phone calls if needed | Dropout rate (patients lost to follow-up), mean number of modules completed for patients and parents, cost per patient, therapist time per patient, satisfaction scale (CSQ-8) | no |
| Aspvall et al., 2020 [10] | Sweden, UK & Australia | Prospective, observational cohort study with assessments at baseline, post-treatment and follow-up | 2 specialist OCD clinics and 1 university clinic | 31 patients (M age=14, SD=2.3) | OCD | "BIP", a 12-module internet-delivered CBT program with 5 parallel modules for parents in the UK and Australia and 12 modules in Sweden, and asynchronous chat-based therapist support with occasional phone calls if needed | Dropout rate (<100% intervention completion), mean number of modules completed, assessment completion rate, therapist time per patient | yes, post-treatment therapist feedback about feasibility and acceptability in open questions about advantages and/or concerns |
| Beaumont et al., 2021 [11] | Australia | Prospective RCT study with assessments pre-, post treatment and 10 weeks after the intervention began and 6 weeks follow up, comparing a computer game-based social skills training intervention with cognitive skills training game as a control group | Research clinic | 70 patients aged 7-12 and their parents (n IG =35) | Social skills with autism spectrum disorders | multimedia-based group intervention, with webinars and video coaching for parents, computer game for children and parent training slides and teacher handouts. Synchronous support from therapist to parents weekly | Dropout rate (parents reporting not completing the 10-week program), mean number of sessions attended, parent rated program satisfaction and child satisfaction | yes, post-treatment open survey questions to parents about their experience with the program |
| Berg et al., 2020 [12] | Sweden | Prospective RCT study with assessments pre-, post treatment and 6 months follow up using a 2x2 design with two factors: with or without learning support and/or chat sessions | NA | 120 patients (M age= 16.97, SD= 1.20) | Anxiety and depression | Transdiagnostic ICBT program with 8 weekly modules and asynchronous therapist support through chat, in addition to half receiving 30 mins weekly synchronous chat sessions and half receiving learning support | Dropout rate (Patients actively dropping out of the study), mean number of modules completed, and mean therapist time per patient per week | no |
| Bjureberg et al., 2018 [13] | Sweden | Prospective cohort-study with assessments at pre-, post-treatment, 3 and 7-month follow up | Secondary mental health clinic | 25 patients and their parents, aged 13-17 | nonsuicidal self-injury (NSSI) | “Online ERITA”, an 11-module online emotion regulation therapy program with 6 modules for parents, weekly asynchronous therapist support via chat, and synchronous text messages and phone calls if inactive online | Dropout rate (number of patients who actively dropped out of the treatment), patient and parent reported program credibility, expectancy and satisfaction (CSQ-8), mean number of modules completed for patients and parents, mean therapist time per patient | yes, post-treatment open survey questions about the patients experiences with the program |
| De Bruin et al., 2018 [14] | Netherlands | Prospective RCT study with assessments at baseline, post-treatment and 2, 6 and 12-month follow up comparing the intervention with face-to-face treatment and waitlist | Primary mental health clinic | 116 patients (M age= 15.6, SD= 1.6) (n IG= 39) | Insomnia | "SleepingSmart", an online CBT program with 6 weekly modules and 2 booster sessions, with asynchronous and synchronous therapist support via chat | Dropout rate (<100% intervention completion) | no |
| Dingwall et al., 2023 [15] | Australia | Prospective cohort study with assessment at baseline and 4 weeks, and qualitative interviews at 4 weeks. | NA | 30 patients (M=14, SD=1.4) | Well-being | “AIMhi-Y”, a 4-week app-delivered treatment of low-intensity, culturally adapted CBT to indigenous youth, with standardizes weekly supportive text messages from the therapist | Dropout rate (<100% intervention completion), recruitment rate, retention rate, and patient reported ease of use, usefulness, satisfaction, cultural relevancy, and acceptability | yes, post-treatment interviews |
| Farmer, 2007 [16] | Canada | Prospective qualitative case study with baseline performance evaluation and needs analysis, bimonthly observation, and post-implementation interviews | Community health centre | 12 health care practitioners treating 26 patients aged 12-18 years | Tobacco addiction | "Smoking Zine", a computer-based, guided self-help program, with synchronous therapist support in groups of 7-8 youths, and during health care visits | Dropout rate (patients lost to follow-up) | yes, post-implementation interviews with health care practitioners |
| Geirhos et al., 2022 [17] | Germany | Prospective RCT study with assessments at baseline, post-treatment and 6 months post baseline follow up | Research/University clinic | 30 patients aged 12-21 (M=16.1; SD= 2.2) (n IG=15) | comorbid depression or anxiety symptoms | "YouthCoachCD", a 7-module computer-delivered CBT program with an introductory session and a daily mood diary app, and therapist chat and phone calls when the patients do not adhere to the treatment | Dropout rate (<80% intervention completion), mean number of modules completed, patient rated intervention satisfaction rate and CSQ-8 and recommendation | yes, reason for dropout is reported |
| Georen et al., 2022 [18] | Sweden | Prospective mixed-method study with qualitative semi structured interviews post-treatment, assessments pre-, mid-, and post-treatment, and 2 follow-ups | Secondary health clinic | 6 patients aged 13-17 | Insomnia in adolescents with ASD | "CBT-I", an 8-module internet-delivered CBT program for insomnia with psychoeducational videos, synchronous therapist bi-weekly support by phone or chat, and supplementary manual for parents | Dropout rate (number of patients who never used the intervention, from qualitative self report) | yes, post-treatment feedback from patients and parents |
| Gladstone et al., 2020 [19] | US | Prospective randomised control study with assessments pre, mid, post and follow up comparing CATCH-IT to active control group receiving online health education | 31 primary care clinics | 369 patients (IG= 193, CG n=176) aged 13 to 18 (M=15.4, SD 1.5) | Depression | "CATCH_IT" , a 14-module internet-delivered behavioural activation, cognitive behavioural and interpersonal psychotherapy program including 6-15 minutes motivational interviewing before and after and three motivational phone call during the program by a general practitioner | Dropout rate (<100% intervention completion), enrolment rate, mean number of program completion | yes, missing scores were quantitively analyzed in relation to demographic variables and symptom severity |
| Jolstedt et al., 2018 a[20] | Sweden | Prospective cohort study with assessments pre, post and follow-up | Mixed primary and secondary health clinic | 19 patients aged 8-12, (M=10.5, SD=1.6) | Anxiety | "BIP" | Dropout rate (number of patients who were not active during the treatment period), mean number of modules completed, patient and parent reported satisfaction | yes, post-treatment clinician reported barriers and facilitators |
| Jolstedt et al. 2018 b [21] | Sweden | Prospective RCT study with assessments pre, post and follow-up | Research unit at a secondary health clinic | 131 patients aged 8–12 (M=10.0, SD=1.4), 1:1 IG CG | Anxiety | "BIP" | Dropout rate (patients lost to follow-up, not providing data post-treatment), mean number of completed modules, patient and parent reported treatment credibility and satisfaction rate, therapist time per patient | yes, dropouts were quantitatively analysed in relation to symptom severity |
| Khan et al., 2021[22] | UK | Prospective cohort study with assessment at baseline, 3 weeks, post treatment and at 3-month follow up, and qualitative interviews with children and their parents at follow up | 2 secondary health clinics | 112 patients aged 9-17 (M=12, SD=2.1), interviews with 20 children and parents, 6 clinicians and 4 therapists | Tic disorder | "ORBIT" | Dropout rate (<4 modules completed), mean number of modules completed, completion rate, patient and parent reported treatment credibility and satisfaction | yes, post-treatment interviews with patients and parents, quantitative analyses of child engagement in relation to parent engagement and therapist support. |
| Khanna & Kendall, 2010 [23] | US | Prospective RCT study with assessments at pre- and posttreatment and 3-month follow-up comparing CCAL to two active control groups: individual CBT and digital self help | NA | 49 patients aged 7–13 (M =10.1, SD=1.6) (n IG=16) | Anxiety | "CCAL", a computer-delivered CBT program with 12 modules with 35-minute levels and video game rewards, and two parent sessions. The last 6 modules are completed with the assistance of a therapist. | Dropout rate (<100% intervention completion), therapist adherence, patient and parent rated satisfaction | yes, reason for dropout is reported |
| Kurki et al., 2018 [24] | Finland | Retrospective qualitative study | 4 secondary and tertiary mental health clinics | 9 nurses (who treated 70 adolescents) | Depression | “Depis-Net”, a 6-module internet-based therapy program with peer communication and weekly asynchronous therapist support via chat | NA | yes, post-treatment therapist experiences and attitudes of the program |
| Larsson et al., 2025 [25] | Sweden | Prospective naturalistic open trial of two groups with or without parental support before, during, and after treatment | 3 primary adolescent mental health care clinics | 24 patients aged 13-18 years. 9 without parental support; 15 with parental support | Anxiety | “iACT”. a guided, internet-based, self-help program called Anxiety Help for Adolescents, with 8 modules over 10 weeks, with asynchronous weekly therapist support, and synchronous in-person support if necessary. Parents received 3 physical meetings during their adolescent’s treatment period. | Dropout rate (number of patients who did not complete 100% of the program), mean number of completed modules | yes, reason for dropout is reported |
| Lilja et al., 2021 [26] | Sweden | Prospective mixed methods study with quantitative assessments pre and post, and qualitative interviews during treatment | 3 primary health clinics | 14 patients and parents (9 aged 13-15, 5 aged 16-18) | Anxiety | "Anxiety help for Adolescents", an internet-delivered CBT program with 8 transdiagnostic modules and asynchronous weekly therapist support through chat. The program relies heavily on exposure therapy as well as Acceptance and Commitment Therapy (iACT) | dropout rate (lost to follow up) | yes, post-treatment feedback from patients |
| Lincke et al., 2025 [27] | Germany | Single-Group, Naturalistic Feasibility Trial with assessments during the intervention period and 90-day follow-up | 2 outpatient university hospital clinics | 36 patients aged 15 (1) | Depression | “e-MICHI” (Manualized Intervention to Cope With Depressive Symptoms, Help Strengthen Resources and Improve Emotion Regulation) an app-delivered cognitive behavioral therapy–based intervention with 6 modules over 6 weeks and 3 face-to-face sessions with a therapist, asynchronous support and synchronous support on demand. | Dropout rate (number of patients who completed the first module but stopped before completion of the whole program), mean number of modules completed, number of messages sent by patients, Therapist rated adherence, patient and therapist rated satisfaction | yes, reason for dropout is reported |
| Mazenc, 2023 [28] | Canada | prospective cohort study with quantitative assessment pre, mid and post treatment and at 3-month follow-up, and follow-up interviews with parents | Secondary health clinic | 91 parents of children aged 7-12 (M=9.4, SD=1.7) | Anxiety | "ACE", a parent-administered internet-delivered CBT program for anxiety with 7 weekly modules and asynchronous therapist support | Dropout rate (<100% intervention completion), parent reported treatment credibility and expectancy, satisfaction, usability | yes, reason for dropout, post-treatment interviews about the experiences with the treatment and quantitative analyses of dropout and engagement in relation to symptom severity and demographic variables |
| Midgley et al., 2021 [1] | UK | Prospective cohort-study with assessments at baseline and posttreatment | Anna Freud National centre | 23 patients aged 16-18 | Depression | Affect-focused psychodynamic internet-based therapy with 8 modules over 10 weeks and 30 minute weekly synchronous support from a therapist | Dropout rate (<100% intervention completion), patient reported acceptability, satisfaction rate, and usability of the program | yes, post-treatment open survey questions about the patients experiences with the program |
| Miklowitz et al., 2021 [29] | US | Prospective cohort study with assessment at baseline and every 9 weeks over 27 weeks | NA | 22 patients (M age= 15.4, SD= 1.8), and their 34 parents | Mood disorders | App-enhanced Functional Family Therapy (FFT), containing 12 modules for family use between 8 family therapy sessions over 4 months | Dropout rate (<100% intervention completion), mean proportion of program completion, patient and parent reported ease of use and satisfaction | no |
| Millier et al., 2021 [30] | US | Mixed methods study of engagement with assessments pre and post treatment | gastroenterology clinics at an academic medical centre | 60 patients aged 9-15 (M=11.7, SD=1.7) | Anxiety in youth with abdominal pain | "ADAPT", an internet-delivered CBT program with 2 in person sessions, 4 web modules and synchronous therapist support via 15-minute phone calls with the child and the caregiver | Dropout rate (lost to follow up), mean proportion of web tools used, videos and hand outs viewed | yes, post-treatment open survey questions |
| Molleda et al., 2017 [31] | US | Retrospective semi structured interviews and focus groups analysed using a general inductive approach | 2 primary health clinics | 9 health care practitioners, and 6 parents of patients aged 12-16 | behavioural problems in Hispanic adolescents | "eHealth Familias Unidas", an internet-delivered parental training program, with 8 modules and synchronous therapist support | Dropout rate (number of patients who actively dropped out of the study) | yes, post-treatment interviews with parents and therapists |
| Nordh et al., 2017 [32] | Sweden | Prospective cohort study with assessments pre, post and follow-up | Secondary health clinic | 30 patients aged 13–17 (M=15, SD=1.22) | Social aniety | a 9-module internet-delivered CBT program over 12 weeks with three group exposure sessions at the clinic, and 5 modules for parents. Both patients and parents receive asynchronous weekly therapist support, and telephone calls if needed | Dropout rate (<2 modules completed), mean number of modules completed for patients and parents, parent and patient reported ease of use | no |
| Nordh et al, 2021 [33] | Sweden | Prospective RCT study with assessments at baseline, posttreatment and 3 month follow up, comparing ICBT to an active control group (internet-delivered support therapy) | Research unit at a secondary health clinic | 103 patients aged 10-17 (M=14.1, SD=2.1) (n IG=51) | Social aniety | internet-delivered CBT with 10 modules, 5 separate parent modules, with asynchronous therapist support and three 30-minute video call sessions | Dropout rate (lost to follow up), mean number of modules completed for patients and parents, therapist rated treatment adherence, parent rated treatment credibility and satisfaction (CSQ-8), therapist time per patient | no |
| Rautio et al., 2023 [34] | Sweden | Prospective cohort study with assessments pre-, post-treatment and at 12-month follow-up | Specialist clinic | 20 patients aged 12-17 (M=15.8, sd=1.3) | Body dysmorphic disorder (BDD) | internet delivered CBT with 12 parallel modules for patient and parent and asynchronous therapist support through chat, phone calls were possible if necessary | dropout rate (<100% intervention completion), mean number of modules completed by patient and parent, therapist rated adherence to treatment, patient and parent rated treatment credibility and satisfaction, therapist time per patient | no |
| Salloum et al., 2015 [35] | US | Retrospective focus-groups and interviews and surveys of satisfaction and treatment barriers | Community mental health center | 6 patients aged 7-13 (M=10.67, SD= 2.42), 7 parents, 3 therapists, 3 project coordinators and 3 administrators | Anxiety | "CCAL" | Patient and parent reported treatment satisfaction (CSQ-8) | yes, post-treatment qualitative interviews |
| Sandin et al., 2020 [36] | Spain | Prospective cohort study with assessment pre and post treatment | Research/University clinic | 12 patients aged 13-18 (M= 15.6, SD= 1.6) | Anxiety and depression | "iUP-A", a computer-delivered CBT program with 8 weekly parallel parent and patient modules, and asynchronous therapist support via chat, and weekly 10-minute phone calls with parents | Dropout rate (patients who actively dropped out of treatment), mean number of modules completed, patient and parent reported program satisfaction | yes, post-treatment open questions to patients and parents to assess their experience with the program |
| Silfvernagel et al., 2015 [37] | Sweden | Prospective observational cohort study with assessments pre, and post intervention | Secondary health clinic | 11 patients aged 14-19 (M=16.8, SD = 1.66) | Anxiety | A tailored internet-delivered CBT, 6-8 weeks with 6-9 modules, with phone calls or face-to-face sessions if required. | Dropout rate (<2 modules completed), mean number of modules completed | no |
| Silk et al., 2020 [38] | US | Prospective cohort study with assessments pre-, post-treatment and at 2 month follow-up | Research/University clinic | 34 patients aged 9–14 (M= 11.4, SD= 1.5) | anxiety disorder | "SmartCAT 2.0", a mobile-delivered interactive and gamified CBT program for anxiety disorders, with face-to-face sessions and asynchronous therapist support via chat | Dropout rate (<100% intervention completion), patient and parent reported program satisfaction (CSQ-8), usability and therapist reported usability | yes, reason for dropout is reported |
| Spence et al., 2008 [39] | Australia | 2 case studies with quantitative and qualitative assessments pre, post and 6-month follow up | NA | 2 patients aged 10 and 17, and their parent | Anxiety | "BRAVE-ONLINE", a computer-based CBT with 10 modules and 5/6 parent modules, asynchronous weekly therapist support, and one 30 min phone call to coach the family in development of an appropriate exposure hierarchy mid-treatment | Proportion of program completion, patient and parent reported expectancy, credibility and satisfaction, therapist time per patient | yes, post-treatment feedback from three open-ended questions to clients about program satisfaction and dissatisfaction, and reported experiences with implementation of the online program from the authors |
| Srivastava et al., 2020 [40] | India | Prospective RCT study with assessment of baseline, mid and post treatment comparing intervention with active control group (treatment as usual) | Tertiary mental health clinic | 21 patients (M age=16, SD=1.5) (n IG=11) | Depression | "SmartTeen", an app-based CBT program with 12 modules, with synchronous face-to-face therapist support | Dropout rate (<100% intervention completion), mean number of completed modules, patient reported acceptability, satisfaction | yes, post-treatment open survey questions |
| Stallard et al., 2011 [41] | UK | Prospective RCT study with assessments at pre and post intervention compared to waitlist | Tertiary clinic | 20 patients aged 11-16 (M=12) (n IG= 10) | Depression and anxiety | "Think, feel, do", a 6 session CD-ROM based CBT program, with synchronous therapist support 30-45 minutes per session | Dropout rate (<100% intervention completion), patient reported program satisfaction and recommendation | yes, post-treatment patient feedback on the experience of the program |
| Stasiak et al., 2018 [42] | New Zealand | Prospective cohort study with assessments pre-, post-treatment and 6 month follow-up. | Primary health clinic | 42 patients (M age=11.1, SD=2.5) | Anxiety | "BRAVE-ONLINE" | Dropout rate (<100% intervention completion), mean number of completed modules by the patients and parents, patient and parent reported treatment satisfaction | yes, post-intervention feedback from patients and parents were collected with open questions about satisfaction with the program |
| Stjerneklar et al., 2018 [43] | Denmark | Retrospective cohort study with semi structured interviews and questionnaires | University clinic | 6 patients (M age= 15, SD=1.79) | Anxiety | "ChilledOut", a 12-week internet-delivered CBT program with 8 modules, with a pamphlet and closed online peer-network for parents and weekly therapist phone calls for patients. Parents are invited to contact the therapist via e-mail a short phone call if needed | Dropout rate (number of patients who actively dropped out of the treatment), mean number of modules completed, parent and patient satisfaction (CSQ-8) | yes, post-treatment open questions about facilitators and barriers |
| Stjerneklar et al., 2019 [44] | Denmark | Prospective RCT study with assessments at baseline, posttreatment, 3 and 12 month follow up, comparing ChilledOut with waitlist | University clinic | 70 patients aged 13-17 (M = 15.0, SD = 1.30) 1:1 IG CG | Anxiety disorder | "ChilledOut" | Dropout rate (patients who had not remained in contact with the therapist for the entire intervention), mean number of modules completed, patient and parent reported satisfaction rate and recommendation (the experience of service questionnaire, scale 1-3) | yes, post-treatment open questions |
| Topooco et al., 2018 [45] | Sweden | Prospective RCT study with assessments at baseline, posttreatment and 6 months follow up comparing ICBT with an active control group | NA | 33 patients (M age= 17.2, SD=1) | Depression | Internet delivered CBT program with 8 weekly modules and 30 min synchronous chat sessions | Dropout rate (patients who did not compete 100% of the treatment), mean number of modules completed and mean therapist time per patient per week | no |
| Topooco et al., 2019 [46] | Sweden | Prospective RCT study with assessments at baseline and posttreatment, comparing the intervention group with waitlist | Community health care centre | 70 patients (M age=17,5, SD=1.15) 1:1 IG, CG | Depression | Internet-delivered CBT with 8 weekly modules and synchronous therapist chat sessions and feedback within 24 hrs on weekdays | Dropout rate (<50% intervention completion), mean number of modules completed, patient reported treatment credibility | no |
| Van Voorhees et al., 2009; 2010; Eisen et al., 2013; [47] [48] [49] | US | Prospective RCT study with assessments pre, post and follow-up comparing motivational interview and brief advice as therapist support for the digital health intervention, and Observational study evaluating the quality of a marketing strategy and fielding experiences for depression prevention | 13 primary care clinics | 84 patients (M age=17.26, SD=1.85) 63 health care practitioners | Depression | "CATCH-IT" | Dropout rate (<50% completion), enrolment rate, cost per enrolee, patient engagement rate, therapist fidelity to MI, mean number of completed modules, Therapist reported program and MI sustainability, credibility and acceptability | yes, post-implementation interviews with health care practitioners and statistical analyses of implementation outcomes in relation to demographic variables and therapist reported feasibility |
| VIgerland, et al., 2024 [50] | Sweden | Prospective open trial with a naturalistic follow-up, with assessments at baseline, after 12 weeks when the treatment period was completed (post-treatment) and 12 weeks after post-treatment (three-month follow-up; primary endpoint). | Non-specialized secondary health clinic | 83 patients aged 13.43 (2.51) | Anxiety | “BIP Anxiety” or “BIP OCD” for children, “BIP Anxiety for Adolescents” for adolescents, with 12 modules and 12 parallell modules for parents and asynchronous therapist support and additional synchronous support on demand | Dropout rate (number of patients who actively dropped out of the study), mean number of completed modules, patient reported treatment satisfaction rate, treatment credibility, and satisfaction, therapist logged therapist time per patient per week and therapist rated patient adherence | yes, reason for dropout is reported and therapist turnover |
| Voerman et al., 2015 [51] | Netherlands | Prospective cohort study with assessments at 7 weeks before treatment, pretreatment, posttreatment, 3 month follow up, comparing intervention with waitlist | NA | 69 patients aged 12-17 (M=14.9, SD=1.1) (n IG= 35) | Chronic Pain | "Move it now", a 7-module internet-delivered CBT program for adolescents with chronic pain, with 2 separate modules for parents, asynchronous therapist-support by email weekly and phone calls every other week | Dropout rate (number of patients who actively dropped out of the study), patient reported treatment satisfaction rate | yes, reason for dropout is reported |
| Waite et al., 2019 [52] | UK | Prospective RCT study with assessments at baseline, post-treatment and 6-month post baseline follow up | Specialist clinic for anxiety disorders | 60 patients aged 13-18 (M = 14.7, SD = 1.42) | Anxiety | "BRAVE for Teenagers-ONLINE", an internet-delivered CBT program with 10 parallel patient and parent modules, each of approximately 60-minute duration and two booster sessions, with asynchronous e-mail feedback and phone calls from an assigned therapist | Dropout rate (<100% intervention completion), patient reported satisfaction rate and recommendation | yes, reason for dropout is reported |
| Weineland et al., 2020 [53] | Sweden | Prospective qualitative study with semistructured interviews pre and post icbt experience | 8 primary health clinics | 14 therapists | Anxiety | "Anxiety help for Adolescents" | NA | yes, pre and posttreatment feedback from therapists about their attitudes and experiences |
| Weintraub et al., 2022 [54] | US | Prospective cohort study with assessments at pre and post treatment | 2 specialist clinics | 31 patients (M age= 15.1, SD=1.5) | Mood or psychotic spectrum disorders | Modified Unified Protocol for Adolescents, an app-enhanced CBT program with 9 weekly group sessions for adolescents and their parents delivered in modules. Therapists evaluate and set homework between sessions in the app | Dropout rate (lost to follow up), mean number of sessions completed, patient and parent reported acceptability | no |
| Wickberg et al., 2022 [55] | Sweden | Prospective observational cohort study comparing ICBT with a benchmark sample of patients receiving face-to-face specialist treatment as usual | Specialist clinic for OCD and related disorders | 22 patients (M age=13.9, SD=1.6) (n IG=54) | comorbid OCD in young people with ASD | 16-week ASD-adapted internet-delivered CBT for OCD, consisting of two age appropriate versions of 14 modules and parallel parent modules, with asynchronous chat support with licenced psychologists and phone calls when necessary | Dropout rate (lost to follow up), mean number of modules completed by patient and parent, therapist rated adherence, patient rated and parent rated credibility and satisfaction (CSQ-8), therapist time per patient | yes, open questions mid treatment about patients experiences and suggestions for improvement, from families at post treatment assessment, and a focus group meeting with the therapists about their experiences and suggestions for improvement. Satisfaction was quantitively analysed in relation to symptom severity. |

**References**

1. Midgley N, Guerrero-Tates B, Mortimer R, Edbrooke-Childs J, Mechler J, Lindqvist K, et al. The Depression: Online Therapy Study (D:OTS)-A Pilot Study of an Internet-Based Psychodynamic Treatment for Adolescents with Low Mood in the UK, in the Context of the COVID-19 Pandemic. Int J Environ Res Public Health. 2021 12 09;18(24):09. PMID: 34948601. doi: 10.3390/ijerph182412993.

2. Van Voorhees B, Gladstone TRG, Sobowale K, Brown CH, Aaby DA, Terrizzi DA, et al. 24-Month Outcomes of Primary Care Web-Based Depression Prevention Intervention in Adolescents: Randomized Clinical Trial. J Med Internet Res. 2020 10 28;22(10):e16802. PMID: 33112254. doi: 10.2196/16802.

3. Drozd F, Vaskinn L, Bergsund HB, Haga SM, Slinning K, Bjorkli CA. The implementation of Internet interventions for depression: A scoping review. Journal of Medical Internet Research. 2016;.18(9):pp. PMID: 2016-58575-020. doi: https://dx.doi.org/10.2196/jmir.5670.

4. Pollak RM, Mortillo M, Murphy MM, Mulle JG. Behavioral Phenotypes and Comorbidity in 3q29 Deletion Syndrome: Results from the 3q29 Registry. J Autism Dev Disord. 2024 Jan 12;12:12. PMID: 38216835. doi: 10.1007/s10803-023-06218-w.

5. Alvarez-Jimenez M, Nicholas J, Valentine L, Liu P, Mangelsdorf S, Baker S, et al. A national evaluation of a multi-modal, blended, digital intervention integrated within Australian youth mental health services. Acta Psychiatrica Scandinavica. 2025 Mar;151(3):317–31. PMID: 39260824. doi: 10.1111/acps.13751.

6. Andren P, Aspvall K, Fernandez de la Cruz L, Wiktor P, Romano S, Andersson E, et al. Therapist-guided and parent-guided internet-delivered behaviour therapy for paediatric Tourette's disorder: a pilot randomised controlled trial with long-term follow-up. BMJ Open. 2019 02 15;9(2):e024685. PMID: 30772854. doi: 10.1136/bmjopen-2018-024685.

7. Andren P, Sampaio F, Ringberg H, Wachtmeister V, Warnstrom M, Isomura K, et al. Internet-Delivered Exposure and Response Prevention for Pediatric Tourette Syndrome: 12-Month Follow-Up of a Randomized Clinical Trial. JAMA Network Open. 2024 05 01;7(5):e248468. PMID: 38700867. doi: 10.1001/jamanetworkopen.2024.8468.

8. Aspvall K, Andren P, Lenhard F, Andersson E, Mataix-Cols D, Serlachius E. Internet-delivered cognitive behavioural therapy for young children with obsessive-compulsive disorder: Development and initial evaluation of the BIP OCD junior programme. BJPsych Open. 2018 May;4(3):106–12. PMID: 2019-79796-001. doi: 10.1192/bjo.2018.10.

9. Aspvall K, Sampaio F, Lenhard F, Melin K, Norlin L, Serlachius E, et al. Cost-effectiveness of Internet-Delivered vs In-Person Cognitive Behavioral Therapy for Children and Adolescents With Obsessive-Compulsive Disorder. JAMA Netw Open. 2021 Jul 1;4(7):e2118516. PMID: 34328501. doi: 10.1001/jamanetworkopen.2021.18516.

10. Aspvall K, Lenhard F, Melin K, Krebs G, Norlin L, Näsström K, et al. Implementation of internet-delivered cognitive behaviour therapy for pediatric obsessive-compulsive disorder: Lessons from clinics in Sweden, United Kingdom and Australia. Internet Interventions-the Application of Information Technology in Mental and Behavioural Health. 2020 Apr;20. PMID: WOS:000550243900011. doi: ARTN 10030810.1016/j.invent.2020.100308.

11. Beaumont R, Walker H, Weiss J, Sofronoff K. Randomized Controlled Trial of a Video Gaming-Based Social Skills Program for Children on the Autism Spectrum. Journal of Autism and Developmental Disorders. 2021 October;51(10):3637–50. PMID: 2007728197. doi: 10.1007/s10803-020-04801-z.

12. Berg M, Rozental A, de Brun Mangs J, Näsman M, Strömberg K, Viberg L, et al. The Role of Learning Support and Chat-Sessions in Guided Internet-Based Cognitive Behavioral Therapy for Adolescents With Anxiety: A Factorial Design Study. Frontiers in Psychiatry. 2020 2020–June–10;Volume 11 - 2020. doi: 10.3389/fpsyt.2020.00503.

13. Bjureberg J, Sahlin H, Hedman-Lagerlof E, Gratz KL, Tull MT, Jokinen J, et al. Extending research on Emotion Regulation Individual Therapy for Adolescents (ERITA) with nonsuicidal self-injury disorder: open pilot trial and mediation analysis of a novel online version. BMC Psychiatry. 2018 10 11;18(1):326. PMID: 30305103. doi: 10.1186/s12888-018-1885-6.

14. de Bruin EJ, Bögels SM, Oort FJ, Meijer AM. Improvements of adolescent psychopathology after insomnia treatment: results from a randomized controlled trial over 1 year. Journal of child psychology and psychiatry, and allied disciplines. 2018;59(5):509–22. PMID: CN-01959502. doi: 10.1111/jcpp.12834.

15. Dingwall KM, Povey J, Sweet M, Friel J, Shand F, Titov N, et al. Feasibility and Acceptability of the Aboriginal and Islander Mental Health Initiative for Youth App: Nonrandomized Pilot With First Nations Young People. JMIR Hum Factors. 2023 Jun 07;10:e40111. PMID: 37285184. doi: 10.2196/40111.

16. Farmer AP. Adoption of an innovation: The story behind preventive services in a community health centre [Dissertation/Thesis]2007.

17. Geirhos A, Domhardt M, Lunkenheimer F, Temming S, Holl RW, Minden K, et al. Feasibility and potential efficacy of a guided internet- and mobile-based CBT for adolescents and young adults with chronic medical conditions and comorbid depression or anxiety symptoms: a randomized controlled pilot trial. BMC Pediatr. 2022 Jan;22(1):15. PMID: WOS:000749189600001. doi: 10.1186/s12887-022-03134-3.

18. Georen L, Jansson-Frojmark M, Nordenstam L, Andersson G, Olsson NC. Internet-delivered Cognitive Behavioral Therapy for insomnia in youth with autism spectrum disorder: A pilot study. Internet Interv. 2022 Sep;29:100548. PMID: 35651733. doi: 10.1016/j.invent.2022.100548.

19. Gladstone T, Buchholz KR, Fitzgibbon M, Schiffer L, Lee M, Voorhees BWV. Randomized Clinical Trial of an Internet-Based Adolescent Depression Prevention Intervention in Primary Care: Internalizing Symptom Outcomes. Int J Environ Res Public Health. 2020 10 22;17(21):22. PMID: 33105889. doi: 10.3390/ijerph17217736.

20. Jolstedt M, Ljotsson B, Fredlander S, Tedgard T, Hallberg A, Ekeljung A, et al. Implementation of internet-delivered CBT for children with anxiety disorders in a rural area: A feasibility trial. Internet Interv. 2018 Jun;12:121–9. PMID: 30135776. doi: 10.1016/j.invent.2017.11.003.

21. Jolstedt M, Wahlund T, Lenhard F, Ljotsson B, Mataix-Cols D, Nord M, et al. Efficacy and cost-effectiveness of therapist-guided internet cognitive behavioural therapy for paediatric anxiety disorders: a single-centre, single-blind, randomised controlled trial. Lancet Child Adolesc Health. 2018 11;2(11):792–801. PMID: 30241993. doi: 10.1016/s2352-4642(18)30275-x.

22. Khan K, Hollis C, Hall CL, Murray E, Davies EB, Andren P, et al. Fidelity of Delivery and Contextual Factors Influencing Children's Level of Engagement: Process Evaluation of the Online Remote Behavioral Intervention for Tics Trial. J Med Internet Res. 2021 06 21;23(6):e25470. PMID: 34152270. doi: 10.2196/25470.

23. Khanna MS, Kendall PC. Computer-assisted cognitive behavioral therapy for child anxiety: results of a randomized clinical trial. Journal of consulting and clinical psychology. 2010;78(5):737–45. PMID: CN-00772841. doi: 10.1037/a0019739.

24. Kurki M, Anttila M, Koivunen M, Marttunen M, Valimaki M. Nurses' experiences of the use of an Internet-based support system for adolescents with depressive disorders. Inform Health Soc Care. 2018 Sep;43(3):234–47. PMID: 28139155. doi: 10.1080/17538157.2016.1269110.

25. Larsson A, Weineland S, Nissling L, Lilja JL. The Impact of Parental Support on Adherence to Therapist-Assisted Internet-Delivered Acceptance and Commitment Therapy in Primary Care for Adolescents With Anxiety: Naturalistic 12-Month Follow-Up Study. JMIR Pediatrics and Parenting. 2025 Jan 03;8:e59489. PMID: 39752209. doi: 10.2196/59489.

26. Lilja JL, Rupcic Ljustina M, Nissling L, Larsson AC, Weineland S. Youths' and Parents' Experiences and Perceived Effects of Internet-Based Cognitive Behavioral Therapy for Anxiety Disorders in Primary Care: Mixed Methods Study. JMIR Pediatr Parent. 2021 11 01;4(4):e26842. PMID: 34723830. doi: 10.2196/26842.

27. Lincke L, Martin-Doring T, Daunke A, Sadkowiak A, Nolkemper DA, Sproeber-Kolb N, et al. Integration of a Mental Health App (e-MICHI) Into a Blended Treatment of Depression in Adolescents: Single-Group, Naturalistic Feasibility Trial. JMIR Formative Research. 2025 May 01;9:e58427. PMID: 40313202. doi: 10.2196/58427.

28. Mazenc KJ. An investigation of engagement in parent-administered, Internet-delivered cognitive behaviour therapy for childhood anxiety: Intervention usage and subjective experience.DP - 2023. Dissertation Abstracts International Section A: Humanities and Social Sciences. 2023;84(5-A):No Pagination Specified. PMID: 2023-33083-281.

29. Miklowitz DJ, Weintraub MJ, Posta F, Walshaw PD, Frey SJ, Morgan-Fleming GM, et al. Development and Open Trial of a Technology-Enhanced Family Intervention for Adolescents at Risk for Mood Disorders. J Affect Disord. 2021 02 15;281:438–46. PMID: 33360365. doi: 10.1016/j.jad.2020.12.012.

30. Miller AK, Ely SL, Barber Garcia BN, Richardson P, Cunningham NR. Engagement during a Mixed In-Person and Remotely Delivered Psychological Intervention for Youth with Functional Abdominal Pain Disorders and Anxiety. Children (Basel). 2021 Sep 02;8(9):02. PMID: 34572207. doi: 10.3390/children8090775.

31. Molleda L, Bahamon M, St George SM, Perrino T, Estrada Y, Correa Herrera D, et al. Clinic Personnel, Facilitator, and Parent Perspectives of eHealth Familias Unidas in Primary Care. J Pediatr Health Care. 2017 May – Jun;31(3):350–61. PMID: 28012799. doi: 10.1016/j.pedhc.2016.11.001.

32. Nordh M, Vigerland S, Ost LG, Ljotsson B, Mataix-Cols D, Serlachius E, et al. Therapist-guided internet-delivered cognitive-behavioural therapy supplemented with group exposure sessions for adolescents with social anxiety disorder: a feasibility trial. BMJ Open. 2017 Dec 14;7(12):e018345. PMID: 29247101. doi: 10.1136/bmjopen-2017-018345.

33. Nordh M, Wahlund T, Jolstedt M, Sahlin H, Bjureberg J, Ahlen J, et al. Therapist-Guided Internet-Delivered Cognitive Behavioral Therapy vs Internet-Delivered Supportive Therapy for Children and Adolescents With Social Anxiety Disorder: A Randomized Clinical Trial. JAMA Psychiatry. 2021 Jul 1;78(7):705–13. PMID: 33978699. doi: 10.1001/jamapsychiatry.2021.0469.

34. Rautio D, Andren P, Gumpert M, Jolstedt M, Jassi A, Krebs G, et al. Therapist-guided, Internet-delivered cognitive behaviour therapy for adolescents with body dysmorphic disorder: A feasibility trial with long-term follow-up. Internet Interv. 2023 Dec;34:100688. PMID: 38034863. doi: 10.1016/j.invent.2023.100688.

35. Salloum A, Crawford EA, Lewin AB, Storch EA. Consumers' and providers' perceptions of utilizing a computer-assisted cognitive behavioral therapy for childhood anxiety. Behav. 2015 Jan;43(1):31–41. PMID: 23886438. doi: 10.1017/s1352465813000647.

36. Sandín B, García-Escalera J, Valiente RM, Espinosa V, Chorot P. Clinical Utility of an Internet-Delivered Version of the Unified Protocol for Transdiagnostic Treatment of Emotional Disorders in Adolescents (iUP-A): A Pilot Open Trial. International Journal of Environmental Research and Public Health. 2020 Nov;17(22):17. PMID: WOS:000594315300001. doi: 10.3390/ijerph17228306.

37. Silfvernagel K, Gren-Landell M, Emanuelsson M, Carlbring P, Andersson G. Individually tailored internet-based cognitive behavior therapy for adolescents with anxiety disorders: A pilot effectiveness study. Internet Interv. 2015 September 01;2(3):297–302. PMID: 606623223. doi: 10.1016/j.invent.2015.07.002.

38. Silk JS, Pramana G, Sequeira SL, Lindhiem O, Kendall PC, Rosen D, et al. Using a smartphone app and clinician portal to enhance brief cognitive behavioral therapy for childhood anxiety disorders. Behav. 2020 Jan;51(1):69–84. PMID: 2019-41438-001. doi: 10.1016/j.beth.2019.05.002.

39. Spence SH, Donovan CL, March S, Gamble A, Anderson R, Prosser S, et al. Online CBT in the treatment of child and adolescent anxiety disorders: Issues in the development of BRAVE-ONLINE and two case illustrations. Behavioural and Cognitive Psychotherapy. 2008 July;36(4):411–30. PMID: 352165018. doi: 10.1017/s135246580800444x.

40. Srivastava P, Mehta M, Sagar R, Ambekar A. Smartteen- a computer assisted cognitive behavior therapy for Indian adolescents with depression- a pilot study. Asian J Psychiatr. 2020 April;50(no pagination). PMID: 2005075842. doi: 10.1016/j.ajp.2020.101970.

41. Stallard P, Richardson T, Velleman S, Attwood M. Computerized CBT (Think, Feel, Do) for Depression and Anxiety in Children and Adolescents: Outcomes and Feedback from a Pilot Randomized Controlled Trial. Behavioural and Cognitive Psychotherapy. 2011 May;39(3):273–84. PMID: WOS:000289726000002. doi: 10.1017/s135246581000086x.

42. Stasiak K, Merry SN, Frampton C, Moor S. Delivering solid treatments on shaky ground: Feasibility study of an online therapy for child anxiety in the aftermath of a natural disaster. Psychother. 2018 07;28(4):643–53. PMID: 27781568. doi: 10.1080/10503307.2016.1244617.

43. Stjerneklar S, Hougaard E, Nielsen AD, Gaardsvig MM, Thastum M. Internet-based cognitive behavioral therapy for adolescents with anxiety disorders: A feasibility study. Internet Interv. 2018 Mar;11:30–40. PMID: WOS:000457134500004. doi: 10.1016/j.invent.2018.01.001.

44. Stjerneklar S, Hougaard E, McLellan LF, Thastum M. A randomized controlled trial examining the efficacy of an internet-based cognitive behavioral therapy program for adolescents with anxiety disorders. PLoS ONE. 2019;.14(9):ArtID e0222485. PMID: 2019-57199-001. doi: https://dx.doi.org/10.1371/journal.pone.0222485.

45. Topooco N, Berg M, Johansson S, Liljethörn L, Radvogin E, Vlaescu G, et al. Chat- and internet-based cognitive–behavioural therapy in treatment of adolescent depression: randomised controlled trial. BJPsych Open. 2018;4(4):199–207. doi: 10.1192/bjo.2018.18.

46. Topooco N, Bylehn S, Dahlstrom Nysater E, Holmlund J, Lindegaard J, Johansson S, et al. Evaluating the Efficacy of Internet-Delivered Cognitive Behavioral Therapy Blended With Synchronous Chat Sessions to Treat Adolescent Depression: Randomized Controlled Trial. J Med Internet Res. 2019 11 01;21(11):e13393. PMID: 31682572. doi: 10.2196/13393.

47. Van Voorhees BW, Fogel J, Pomper BE, Marko M, Reid N, Watson N, et al. Adolescent Dose and Ratings of an Internet-Based Depression Prevention Program: A Randomized Trial of Primary Care Physician Brief Advice versus a Motivational Interview. J. 2009;9(1):1–19. PMID: 20694059.

48. Eisen JC, Marko-Holguin M, Fogel J, Cardenas A, Bahn M, Bradford N, et al. Pilot Study of Implementation of an Internet-Based Depression Prevention Intervention (CATCH-IT) for Adolescents in 12 US Primary Care Practices: Clinical and Management/Organizational Behavioral Perspectives. The Primary Care Companion to CNS Disorders. 2013;15(6). PMID: 24800110. doi: 10.4088/PCC.10m01065.

49. Van Voorhees BW, Watson N, Bridges JF, Fogel J, Galas J, Kramer C, et al. Development and pilot study of a marketing strategy for primary care/internet-based depression prevention intervention for adolescents (the CATCH-IT intervention). Primary Care Companion to the Journal of Clinical Psychiatry. 2010;12(3). PMID: 20944776. doi: 10.4088/PCC.09m00791blu.

50. Vigerland S, Fredlander S, Aspvall K, Jolstedt M, Lenhard F, Mataix-Cols D, et al. Effectiveness of internet-delivered cognitive behavioural therapy for anxiety and obsessive-compulsive disorders within routine clinical care in rural Sweden. Internet Interventions. 2024 Jun;36:100738. PMID: 38617387. doi: 10.1016/j.invent.2024.100738.

51. Voerman JS, Remerie S, Westendorp T, Timman R, Busschbach JJ, Passchier J, et al. Effects of a Guided Internet-Delivered Self-Help Intervention for Adolescents With Chronic Pain. The journal of pain. 2015;16(11):1115–26. PMID: CN-01137503. doi: 10.1016/j.jpain.2015.07.011.

52. Waite P, Marshall T, Creswell C. A randomized controlled trial of internet-delivered cognitive behaviour therapy for adolescent anxiety disorders in a routine clinical care setting with and without parent sessions. Child Adolesc Ment Health. 2019 Sep;24(3):242–50. PMID: 32677216. doi: 10.1111/camh.12311.

53. Weineland S, Ribbegardh R, Kivi M, Bygdell A, Larsson A, Vernmark K, et al. Transitioning from face-to-face treatment to iCBT for youths in primary care - therapists' attitudes and experiences. Internet Interv. 2020 Dec;22:100356. PMID: 33318951. doi: 10.1016/j.invent.2020.100356.

54. Weintraub MJ, Ichinose MC, Zinberg J, Done M, Morgan-Fleming GM, Wilkerson CA, et al. App-enhanced transdiagnostic CBT for adolescents with mood or psychotic spectrum disorders. J Affect Disord. 2022 08 15;311:319–26. PMID: 35594972. doi: 10.1016/j.jad.2022.05.094.

55. Wickberg F, Lenhard F, Aspvall K, Serlachius E, Andren P, Johansson F, et al. Feasibility of internet-delivered cognitive-behavior therapy for obsessive-compulsive disorder in youth with autism spectrum disorder: a clinical benchmark study. Internet Interv. 2022;28. PMID: CN-02375952. doi: 10.1016/j.invent.2022.100520.

56. Long HA, French DP, Brooks JM. Optimising the value of the critical appraisal skills programme (CASP) tool for quality appraisal in qualitative evidence synthesis. Research Methods in Medicine & Health Sciences. 2020;1(1):31–42. doi: 10.1177/2632084320947559.

57. Viswanathan M, Patnode CD, Berkman ND, Bass EB, Chang S, Hartling L, et al. Recommendations for assessing the risk of bias in systematic reviews of health-care interventions. Journal of Clinical Epidemiology. 2018 2018/05/01/;97:26–34. doi: https://doi.org/10.1016/j.jclinepi.2017.12.004.

58. Gelman A, Vehtari A, Simpson D, Margossian CC, Carpenter B, Yao Y, et al. Bayesian workflow. arXiv preprint arXiv:201101808. 2020. doi: https://doi.org/10.48550/arXiv.2011.01808.

59. Rognli EW, Zahl-Olsen R, Rekdal SS, Hoffart A, Bertelsen TB. Editorial perspective: Bayesian statistical methods are useful for researchers in child and adolescent mental health. J Child Psychol Psychiatry. 2023 Feb;64(2):339–42. PMID: 35818323. doi: 10.1111/jcpp.13662.

60. Reis DJ, Kaizer AM, Kinney AR, Bahraini NH, Holliday R, Forster JE, et al. A Practical Guide to Random-Effects Bayesian Meta-Analyses With Application to the Psychological Trauma and Suicide Literature. Psychol Trauma-Us. 2023 Jan;15(1):121–30. PMID: WOS:000827803000001. doi: 10.1037/tra0001316.

61. van Buuren S, Groothuis-Oudshoorn K. mice: Multivariate Imputation by Chained Equations in R. Journal of Statistical Software. 2011 12/12;45(3):1 – 67. doi: 10.18637/jss.v045.i03.

62. Bürkner P-C. brms: An R Package for Bayesian Multilevel Models Using Stan. Journal of Statistical Software. 2017 08/29;80(1):1 – 28. doi: 10.18637/jss.v080.i01.
