# Supplementary material for: Barriers to and Facilitators of Implementation of Internet-Delivered Therapist-Guided Therapy in Child and Adolescent Mental Health Services: Systematic Review and Bayesian Meta-Analysis
Source: J Med Internet Res. 2025 Dec 22;27:e83543. doi: 10.2196/83543 (PMC12721491; doi:10.2196/83543)
Supplement: Multimedia Appendix 4 [file jmir-v27-e83543-s004.docx]

Appendix 4– Themes and quotes from qualitative synthesis

| Facilitators (patient factors) |
| --- |
| **Need for flexibility**  “proceed through website at own pace and convenience” [12]  “Other advantages mentioned by adolescents and parents were: (a) the comfort of home” [40]  “flexibility” [44]  “Participating children and their parents reported the ICBT program allowed flexibility” [52]  “Another advantage of this independence was the opportunity to work when and how it suited them. The young people described how they worked on the treatment on their own and that it was up to them to formulate goals and implement changes at their own pace. Several described how they adapted their time or work with the treatment to fit in with other demands in their life. Being able to pause the treatment or adjust when they worked with it to continue to meet their school’s requirements was an important benefit for most young people” [22]  “Many participants believed that, for patients suited to the treatment, iCBT can be more efficient than standard CBT, as the treatment platform is available at any time, and accessible from any place.” [50]  “They frequently commented on the convenience of using an online program at home, in their own time, and at their own pace”[39]  “three open-ended questions provide information on what the clients liked most about the program, what they liked least, and which parts of the program they perceived to be most important components of therapy. case 1 reported that they liked the self-directed nature of the program because they were able to complete sessions in their own time and at their own pace.” [36]  “facilitators' and parents' experience with flexibility and accessability of the program) [28]  “perceiving MOST as a valuable resource and a supportive community with 24/7 availability and the flexibility to go at their own pace” [1] |
| **Need for alternative to face-to-face consultations**  “virtual environment non-judgemental”[12]  “anonymity and less shame in comparison to regular courses of therapy. Some participants believed that iCBT is suitable for patients who have difficulty visiting a clinic, and that this might render therapy accessible to those who would otherwise go without treatment. (And) elimination of transportation time and costs” [40]  “lower median household income was significantly associated with higher efficacy and higher implementation” [44]  “Some participants believed that iCBT is suitable for patients who have difficulty visiting a clinic, and that this might render therapy accessible to those who would otherwise go without treatment. Some participants expressed hopes that iCBT would lessen their emotional strain, since it was seen as less emotionally demanding than face-to-face treatments. In general, the participants felt that iCBT improved their work by increasing task variety and lessening the stress sometimes associated with face-to-face psychotherapy.” [50]  “Young people valued connecting with others experiencing simi lar challenges, which normalised their experience and fostered a sense of connection and belonging.” [1] |
| **Capability for self-management**  “potential to encourage patient empowerment and self-management”[12]  “the format was said to encourage the child's independence” [52]  “Most young people highlighted their ability to keep the treatment work to themselves as an important positive experience” [22]  “The participants reported better outcomes with patients who can maintain consistent discipline, accept personal responsibility, and enjoy working in an independent manner”[50] |
| **Educated, resourceful & engaged parents**  “lower percentage of ethnic minority patient participants was significantly associated with higher adoption and higher reach, and higher median household income was significantly associated with higher maintenance and higher public health impact” [44]  “Higher pre-treatment child anxiety symptoms, lower pre-treatment parent mental health symptoms, and higher levels of parent education were associated with a greater likelihood of program completion. Parent education was the only statistically significant demographic predictor of completion. “ [24]  “Parental support could compensate for low motivation.” [50]  “Factors associated with higher engagement factor scores included higher parental engagement as evidenced by the number of parental chapters completed (n=111; ρ=0.73; P<.001), and more therapist time for parents (n=111; ρ=0.46; P<.001). A multiple linear regression indicated that parents’ chapter completion (β=.69; t110=10.18; P<.001) and therapist time for parents (β=.19; t110=2.95; P=.004) were the only significant independent predictors of child engagement factor scores. Many of the parents interviewed outlined that they were often the main motivating force behind their child’s level of engagement by reminding their child to practice the learned techniques” [18]  “The telephone support (one had chat-based support) was helpful according to the participants. The parents (mothers) planned and organized all that was needed, e.g., adding the treatment to the participant's daily schedule, and they all helped the participants to get started with the modules and homework. They reported also helping them to complete the tasks, such as looking at the modules together. According to some (three), participants, the telephone support was perceived as a checkpoint that prompted the participant to do the homework” [14]  “Parents were particularly positive about the parent component of the program that guided them through their child’s treatment, giving the whole family tips on managing anxiety” [39]  “the pivotal role therapist support could play in keeping parents on track throughout the program”[39] |
| **Positive attitudes to treatment content, format, clinic, efficacy and aim**  “symptom severity at post-treatment, suggesting that clinical improvement was the main driver of participant satisfaction. ‘How much do you expect you/your child will improve with this treatment?’ (from 1 = not at all improved to 5 = very much improved), the children answered on average 4.4 (s. d. = 0.5) and the parents 4.4 (s.d. = 0.7)” [52]  “case 2 reported that the program had taught them both skills to manage anxiety more effectively and cope better with future anxiety-provoking situations” [36]  “(e) the intervention was effective; and (f) skills were used beyond the duration of the program. Parents: An Ambivalent Attitude (… ) more negative parent beliefs about child anxiety predicted higher objective engagement but were also associated with higher child anxiety scores across treatment. Higher credibility and content satisfaction were associated with higher subjective engagement” [24]  “All adolescents in the study, regardless of their previous experience with psychological treatment, described being offered treatment on the internet as something new. Most young people described feeling uncertain about what it would mean to work with their mental health via the internet. Several said they were offered internet treatment at their health unit as an alternative and that they saw it as an opportunity to get help faster, which contributed to a more neutral and positive attitude” [22]  “self-referred versus clinic referred (P=.04) gave higher engagement; parental trust in the clinic)”[18]  “parents' relationship with health care provider and trust in the clinic. perception of the programs content and aim being important” [28] |
| **Facilitators (Intervention factors)** |
| **Engaging format**  “interactive, fun and appealing to youth, more engaging than text-based format, credible website” [12]  “The young people in the study were consistently positive about the treatment program and would recommend it to others. They described various components, tools, and metaphors from the program that they had been thinking about or had worked with. Adolescents appreciated how the program alternated between text, pictures, and films, and that several people with different anxiety problems were presented in the program” [22]  “Young people and their parents commented that the program gave them valuable skills and strategies. The child-friendly format (e.g., animations, bright and captivating imagery and sound effects, ease of use, simple “to the point” scenarios) made the program enjoyable for young people. Some parents commented that the computer format was more engaging for their children than talking to someone” [39]  “the content was engaging” [24]  “On the whole, parents agreed with their child that the dose received was just right, with a parent claiming that if it were longer, it would have negatively affected engagement levels) engaging features like the comics and toolkit” [1] |
| **Technical ease of use**  “All six participants found the Chilled-Out program easy to use. All participants liked the program being structured around 8 separate modules, thereby making the content more manageable and offering the possibility of revisiting program elements that were at first sight hard to comprehend. Several of them explained that they surfed online daily, making it easy to familiarize with and use Chilled Out. Three participants (Participant 2, Participant 3, Participant 6) expressed the notion that it was not the website itself or the different online exercises that had been difficult, but the fact that they had to expose themselves ‘in real life’ to the things they feared the most” [40]  Participating children and their parents reported the ICBT program to be accessible. The content was experienced as helpful and informative” [52]  “The participants reported satisfaction and positive experiences regarding treatment structure. They found it easy to follow, limiting mental energy required since the structure was the same and predictable e.g., the disposition using the weekend to go through the new module which was delivered at the end of a week. Most of the participants reported that homework was demanding or boring. On other hand, it was reported as helpful in solving problems and useful” [14]  “the layout was user-friendly and the intervention was accessible” [24]  “ease of use, ease of correcting mistakes in the platform, visual design, layout. not needing parent consent” [25]  “The division into modules and the standardization of content was predominantly seen as positive” [50]  “perceiving MOST as a valuable resource and a supportive community with userfriendly navigation” [1] |
| **Structured & focused treatment content, Standardization & consistency in therapeutic relationship**  “standardize health care provided to clients, track of client's care, primary and secondary providers may vary but the main MD always the same” [12]  “Therapists also appreciated that the ICBT program brought structure to the treatment and helped to keep the intervention focus on the OCD” [52]  “One reported advantage with ICBT was the reduced risk of therapist-drift (i.e., not following the treatment manual correctly), which made it easier for the clinicians to focus on exposure training.” [16]  “The clear structure of the program was perceived to ensure that all important treatment components are delivered, and that the patient receives evidence-based and equivalent care” [50]  “positive receptiveness from demonstrated effectiveness” [32]  “feasibility screening was significantly associated with higher maintenance and higher public health impact. feasibility of the Internet component of the intervention was significantly associated with higher efficacy and higher implementation”[44]  “The working alliance was the only factor associated with both objective and subjective engagement (i.e., a stronger working alliance predicted higher engagement; parents' relationship with health care provider)” [24]  “perception of the programs content and aim being important” [28] |
|  |
| **Facilitators (therapist factors)** |
| **Positive attitudes towards treatment efficacy, ease of use, new work tasks and time-efficiency**  “clinicians experienced the treatment as time saving compared to regular face-to-face CBT” [16]  “Many participants expressed a hope that iCBT would enable them to work more flexibly, that is, remotely and according to their own schedule. There were also expectations that iCBT would increase task variety, some participants expressed hopes that iCBT would lessen their emotional strain, since it was seen as less emotionally demanding than face-to-face treatments. Using a treatment program with a pre-determined structure was seen as a cognitive relief, as the time and energy normally spent on case conceptualization and session preparation could be cut drastically. The participants collectively expressed confidence that iCBT can have good treatment results. most participants believed that iCBT allows more patients to receive care with fewer delays, and iCBT was therefore considered a time efficiency treatment modality. “[50]  “Flexibility to use different therapists and easy to use” [32]  “nurses' attitudes and technology related factors, user friendliness, user-centred design, safety of the system,” [20]  “Therapists could buy-in to the intervention” [28]  Qualitative feedback from the therapists indicated that they particularly appreciated the inclusion of a mood diary due to its potential to monitor and reflect on emotional change.” [23] |
|  |
| **Facilitators (organizational factors)** |
| **Continuous training & education**  “Resources for self-directed learning, continuing education targeting specific populations, more training on smoking cessation methods” [12]  “Problem solving was accomplished by offering educational programs” [44]  “Designated staff training time, adjustments to productivity requirements until everyone is trained, clinical supervision and hands on training about implementing the program” [32] |
| **Leadership-support & structure for implementation**  “Regular clinic team meetings to discuss smoking cessation issues, regular meetings to address teamwork and QI issues for professional development in behaviour change, appoint health educator to coordinate and deliver smoking cessation programs” [12]  “With small incentive gifts (ie, food), assign person to order and stock health education materials and update and seek new material. Most practices reported problem solving was successful in implementing the marketing plan and was more easily accomplished in small practices”[44]  “Implementation support weekly”[32]  “Organizational support, having an implementation plan, support from the nurse manager”[20]  “Factors associated with higher engagement factor scores included participants enrolled at the London site versus the Nottingham site (P=.01)” [18] |
| **Clear guidelines for intervention delivery**  “Monitoration of practitioner adherence, implement evidence-based medicine using best practices guidelines, place best practices guidelines in chart, local consensus process for developing clinical practice guidelines for continuing care” [12]  “To develop guidelines regarding which patients may be best suited to ICBT in terms of patient motivation prior to initiating ICBT, have routines for risk monitoring, and the importance of therapist training on how to deliver ICBT (e.g. how to give optimal support online and cope with inactive participants” [6]  “Phone consultations with physicians in the small and intermediate size practices. Larger practices needed assistance in creating formal instructions; having an implementation plan ) pamphlets, build coalition with community partners, inform practitioners and staff of services and resource options, develop communication strategy within the organization : communication sheet, prescription box, consult form, list of services and resource options compiled and available for practitioners, access via website, listserv, e-newsletter for linkage and networks”[44]  “Having a marketing and outreach plan”[32] |
| **Barriers (patient factors)** |
| **Lack of time and motivation for self-management**  “time consuming to navigate the entire website. lack motivation, youth has to be motivated to move through entire website” [12]  “Two (7%) would, however, have liked more time” [41]  “Time: four adolescents and one parent explained that they had found it difficult to complete the program within the given time frame of 12 weeks. Also, they had been struggling fitting program work and phone calls into their busy schedules” [40]  “Child- and parent reported measures were missing mostly due to the child and/or parent forgetting or not wanting to login to the platform” [16]  “Some experienced the program as time limited, while others believed that more log-ins would have helped to keep their work with the program more consistent” [22]  “a mother reported having to quit her own studies to support her son. hard to find time for the telephone call which led to their “routine being disturbed” and for the homework. one other participant reported difficulties getting started (procrastination) and difficulties due to lack of planning/organizing, and one reported difficulty staying focused” [14]  “Some participants also said that, for patients with clearly delineated problems of limited severity, the iCBT program is sometimes unnecessarily long and comprehensive, and that a conventional treatment for those patients might be completed in just a few sessions, compared to the 8 weeks needed for Anxiety Help for Adolescents. The participants felt that iCBT allowed patients with low motivation, patterns of avoidance, and/or procrastination tendencies to postpone or skip treatment activities more easily. The general impression among the participants was that compliance was lower in iCBT than in standard, face-to-face CBT. The participants reported that, because of poor patient involvement, iCBT treatments are sometimes prolonged, taking up to twice as long as intended to complete)reason for dropout” [50]  “Doing weekly sessions and homework tasks became challenging for some” [39]  “too much time or effort required. Reasons for no uptake of the intervention were: no motivation; non-completers had lower levels of engagement than completers, including lower numbers of lessons accessed, messages sent by participant, and total logins”[13]  “Two participants withdrew after the first session, one due to an unexpected relocation and one due to time constraints” [35]  “one reported a lack of time for the intervention”[33]  “Three of the generated themes related to program challenges. The themes were: (a) motivation as a barrier to implementation; (b) stressful life events as a barrier to implementation (c) time commitment as a barrier to implementation”[24]  “one withdrew from the study due to distance/time burden”[19]  “no time, forgetting about it, its repetitiveness, and having better things to do”[11]  “Some parents found it difficult to support their children because of hectic schedules, which was captured by the theme of busy lives. Some parents found motivating their child to engage very challenging” [18]  “the feasibility of finding time to implement the learned strategies with their children, relaying concern that it would be difficult to take time out of their schedules to do so.” [27]  “Two families dropped out after pretesting, but before beginning training because changes in their family commitments and schedules prevented them from being able to commit to a consistent training schedule. motivation difficulties may serve as obstacles in completing the module. lack of interest”  [7]  “scheduling conflicts; lack of time management, scheduling difficulties, childcare problems (in both groups, lack of time for school-work”[25]  “intervention too time-consuming (N=2)” [48]  “too time consuming to combine with school”[37]  “as well as for those who have not responded sufficiently to previous treatment or were ambivalent to CBT”[6]  “Some children experienced the treatment content as too extensive, causing boredom and concentration issues. Some parents reported difficulties to motivate their children to engage regularly with the treatment, Therapists also noted that those who had executive functioning problems and negative experiences of previous CBT treatment had more problems adhering to ICBT”[52]  “online delivery has the advantage of flexibility for the family, it is also easy to put off completion of sessions when competing situations arise”[36]  “lack of child engagement”[7]  “Too time consuming (n=2)”[21] |
| **Clinical complexity & severity**  “Therapists perceived that the treatment was less suitable for patients with more complex symptoms such as mental compulsions, rigid rules, and reading/writing compulsions” [6]  “elevated depressed mood at baseline”[44]  “The reason for dropping out was that these families during treatment were judged to be in need of interventions other than CBT for anxiety, e.g., due to school problems” [16]  “drop outs had higher parent-reported child anxiety symptoms (t=2·021, p=0·048), lower parent-reported child quality of life (t=2·269, p=0·027), and a higher number of comorbid diagnoses (t=2·678, p=0·009) at baseline than those who remained in the study”[17]  “Some children did not feel represented by the case vignettes presented when their particular type of OCD was not exemplified” [52]  “Two adolescents withdrew from the study during the waitlist period due to risk of suicide, which had not been present at recruitment”(86)  “several young people said they wanted the program to be even more individualized” [22]  “Some participants expressed concern that their patients' problems were partly due to circumstances and relational patterns in the family environment and that the clear individual focus on the child in iCBT may make it difficult to identify and work with such family factors. Both before and after the implementation of iCBT, the participants underlined the importance of careful patient selection. The term “the right patient” was used repeatedly to describe which patient is suited and which is not suited for treatment. All participants shared experiences of difficulties, and even failure, in treating patients for whom iCBT was less suitable than traditional face-to-face CBT. It is also, according to the participants, important that the patient suffers primarily from anxiety, rather than depression, and that his or her symptoms are not too severe. Although the treatment program is designed to be transdiagnostic, the participants believed that patients with more wide-ranging and long-standing problems should probably be excluded. Several participants expressed that, because of the cognitive emphasis, patients with learning difficulties, neuropsychiatric conditions, and/or dyslexia might find the program difficult to follow.”[50]  “ two were withdrawn due to worsening symptoms”[19]  “The design of appropriate hierarchies is a particular challenge where the child has multiple anxiety disorders, and other comorbid problems. Although the core material of the program is common to the treatment of all anxiety disorders, there is a need for adjunct modules that provide additional therapy with content specific to these presenting problems”[36]  “this was even more challenging for those with children who have comorbidities”[18]  “worsening psychological complaints (n=2)”[48]  “therapists report reasons for patient dropout as other problems in need of treatment”[47] |
| **Preferences for other treatment formats**  “one (3%) expressed the need for face-to-face sessions with the therapist”[41]  “Some families wished for more support in real time, such as telephone calls or in-person meetings [48]  “not satisfied with therapeutic support (n=2), )therapists report reasons for patient dropout as the program being too stressful and preferring to meet face-to-face”[47] |
| **Barriers (Intervention factors)** |
| **Technical difficulties**  “Technical difficulties; four adolescents and one parent reported having experienced technical issues such as slow loading videos or problems logging in to the program” [40]  “most young people also described the disadvantages of working via the internet. In many cases, they lacked confidence in their own ability to work therapeutically via the internet” [22]  “Regarding technical improvements, the participants suggested a written version of the videoclips in the modules, information about the length of the videos in the modules, and that the structure of the webpage could be easier to get acquainted with and the possibility of saving the exercises on the phone instead of needing to log on to the webpage to get access to the files, to make them more easily accessible”[14]  “The key concerns included technical glitches or limitations (e.g., incompatibility with certain computer settings/ devices, sessions not saving correctly, not being able to play relaxation tracks on a stereo)”[39]  “Barriers to use included not having the app on their own phone (due to it only being available on Android devices for the trial)”[11]  “Two child participants reported technical issues with the ORBIT platform, which was related to intermittent problems with connectivity”[18]  “difficulty of logging into the platform (one-time passcode, two-step verification)(2/21), too much reading)”[25]  difficulties with internet access, website login”[27]  “4/57 (7%) of originally included participants dropped out due to technical problems. Access to free Wi-Fi seems to be essential for adolescents to be able to use the app in their living environments. Ways to use the app offline would be desirable to make it accessible to even more patients”[23] “Selected examples include making MOST a smartphone app (now available), voice and video chat, easy content search, and better navigation, more content topics, faster human response times and notifications.”[1] |
| **Lack of adjustments to age & maturity**  “missing scores at 24 months were associated with CATCH-IT higher age at baseline, Immaturity[15]  two parents worried that adolescents might not be mature enough to go through the program on their own and ignore the many distractions that they face in their homes and daily lives in general” [40]  “Participants further felt, based on developmental psychological theories on child development, that patients should ideally be in their upper teens”[50]  “Some of the older CYP felt that the content and presentation of the intervention were childlike and aimed more toward younger participants” [18]  “confusing program content or content not challenging enough” [32] |
| **Time-consuming when used simultaneously as face-to face consultations**  “computers at the point-of-care disruptive to flow of visit and rapport building, inadequate time to navigate website with client during health visit” [12]  “There was a shared opinion among all participants that, when working with patients for whom iCBT works less well, time efficiency drops, as the therapist is required to complement the treatment with repeated notifications, phone calls, and physical appointments meant to motivate the patient and individually tailor the treatment to the non-responding patient. The participants reported that, because of poor patient involvement, iCBT treatments are sometimes prolonged, taking up to twice as long as intended to complete”[50]  “Only a few of the therapists were involved during the whole study period and thus did not have time to become skilled ICBT therapists. Difficulties managing ICBT as well as their ordinary clinical workload were one stated reason for therapist turnover. “[47] |
| **Barriers (therapist factors)** |
| **Insecurity in clinical assessments of treatment progress & compliance without face-to-face contact**  “additional time required to assess quality of information client brings, difficult tot track youth's use” [12]  “Reported difficulties were to get a detailed assessment of how much some patients adhered to the ERP exercises” [6]  “Clinicians also reported being unsure how to handle inactive families and children not responding to treatment, e.g., where and when to terminate ICBT and offer something else instead. It was for example difficult for the clinicians to determine whether inactivity (i.e., not logging in working in the platform) meant that the child was not practising exposure tasks”[16]  It is much harder for the therapist to explore factors such as treatment resistance and non-compliance over the internet compared to face-to-face contact. The reduced amount of verbal communication and absence of visual cues also reduces the amount of information available to the therapist. difficulties that the authors have experienced in implementing the online program with other cases: One of the greatest challenges has been the design of appropriate exposure hierarchies. Despite inclusion of a phone call to assist with this process in the child program, and the addition of detailed guidelines in the adolescent program, the development of an appropriate hierarchy online, with minimal therapist contact, is difficult”[36] |
| **Negative attitudes towards treatment format, safety, time-efficiency & aim**  “For willingness, ratings were in the “strongly disagree/disagree” range.” [44]  “some expressed concerns about organizational motivations for introducing iCBT. There were fears that iCBT is being implemented in health care primarily to meet the needs of the organization for a quick, cheap, and standardized intervention. Prior to implementation, some participants expressed a lack of trust in information technology (IT), based on the concern that it could at any time interfere with their work” [50]  “nurses attitudes” ( low levels of study interest by nursing staff or physicians (2 large primary care practices) [20]  low levels of study interest by nursing staff or physicians[46] |
| **Barriers (organizational factors)** |
| **Lack of time and resources to start up the implementation**  “lack time resources, lack capacity for adapting and leading change. lack quality improvement tools and resources for change” [12]  “lack of local mental health specialists to treat cases of major depression identified by screening, difficulty implementing screening within a busy practice schedule. difficulty incorporating the shared decision-making style of motivational interviewing into their counseling approach.” [44]  “A total of 4,691 adolescents were lost to screening barriers, and 2,443 adolescents were lost to enrollment barriers. start-up costs, technical assistance, staff turnover and leave” [15]  “limited space, recruitment time creating disruption to the treatment flow” [28]  “clinicians with minimal training in ICBT”[47] |
| **Competing demands**  “competing demands for change” [12]  “limited practice time to manage follow-ups with other competing priorities” [44] |
| **Lack of clear structures & procedures for patient flow**  “lack structural supports, minimal leadership, lack common goals and direction” [12]  “Common barriers included lack of established procedures for depression screening (1 small primary care practice), unrelated practice management problems (2 medium size primary care practices), and the need to create new policies in larger practices (2 primary care practices)” [44]  “The lack of clear guidelines on parental involvement was a source of collective concern among the participants. There was no consensus as to how to handle patient inactivity, although many participants described ambitious attempts to increase patients' motivation. Other participants took a more passive stance, as they felt that adding telephone calls and even clinic visits would mean to deviate from the treatment guidelines and add substantially to their own workload. These participants felt that it was important not to turn iCBT into a “blended” treatment, where it is supplemented by components of conventional, face-to-face CBT”[50] |

**References**

1. Midgley N, Guerrero-Tates B, Mortimer R, Edbrooke-Childs J, Mechler J, Lindqvist K, et al. The Depression: Online Therapy Study (D:OTS)-A Pilot Study of an Internet-Based Psychodynamic Treatment for Adolescents with Low Mood in the UK, in the Context of the COVID-19 Pandemic. Int J Environ Res Public Health. 2021 12 09;18(24):09. PMID: 34948601. doi: 10.3390/ijerph182412993.

2. Van Voorhees B, Gladstone TRG, Sobowale K, Brown CH, Aaby DA, Terrizzi DA, et al. 24-Month Outcomes of Primary Care Web-Based Depression Prevention Intervention in Adolescents: Randomized Clinical Trial. J Med Internet Res. 2020 10 28;22(10):e16802. PMID: 33112254. doi: 10.2196/16802.

3. Drozd F, Vaskinn L, Bergsund HB, Haga SM, Slinning K, Bjorkli CA. The implementation of Internet interventions for depression: A scoping review. Journal of Medical Internet Research. 2016;.18(9):pp. PMID: 2016-58575-020. doi: https://dx.doi.org/10.2196/jmir.5670.

4. Pollak RM, Mortillo M, Murphy MM, Mulle JG. Behavioral Phenotypes and Comorbidity in 3q29 Deletion Syndrome: Results from the 3q29 Registry. J Autism Dev Disord. 2024 Jan 12;12:12. PMID: 38216835. doi: 10.1007/s10803-023-06218-w.

5. Alvarez-Jimenez M, Nicholas J, Valentine L, Liu P, Mangelsdorf S, Baker S, et al. A national evaluation of a multi-modal, blended, digital intervention integrated within Australian youth mental health services. Acta Psychiatrica Scandinavica. 2025 Mar;151(3):317–31. PMID: 39260824. doi: 10.1111/acps.13751.

6. Andren P, Aspvall K, Fernandez de la Cruz L, Wiktor P, Romano S, Andersson E, et al. Therapist-guided and parent-guided internet-delivered behaviour therapy for paediatric Tourette's disorder: a pilot randomised controlled trial with long-term follow-up. BMJ Open. 2019 02 15;9(2):e024685. PMID: 30772854. doi: 10.1136/bmjopen-2018-024685.

7. Andren P, Sampaio F, Ringberg H, Wachtmeister V, Warnstrom M, Isomura K, et al. Internet-Delivered Exposure and Response Prevention for Pediatric Tourette Syndrome: 12-Month Follow-Up of a Randomized Clinical Trial. JAMA Network Open. 2024 05 01;7(5):e248468. PMID: 38700867. doi: 10.1001/jamanetworkopen.2024.8468.

8. Aspvall K, Andren P, Lenhard F, Andersson E, Mataix-Cols D, Serlachius E. Internet-delivered cognitive behavioural therapy for young children with obsessive-compulsive disorder: Development and initial evaluation of the BIP OCD junior programme. BJPsych Open. 2018 May;4(3):106–12. PMID: 2019-79796-001. doi: 10.1192/bjo.2018.10.

9. Aspvall K, Sampaio F, Lenhard F, Melin K, Norlin L, Serlachius E, et al. Cost-effectiveness of Internet-Delivered vs In-Person Cognitive Behavioral Therapy for Children and Adolescents With Obsessive-Compulsive Disorder. JAMA Netw Open. 2021 Jul 1;4(7):e2118516. PMID: 34328501. doi: 10.1001/jamanetworkopen.2021.18516.

10. Aspvall K, Lenhard F, Melin K, Krebs G, Norlin L, Näsström K, et al. Implementation of internet-delivered cognitive behaviour therapy for pediatric obsessive-compulsive disorder: Lessons from clinics in Sweden, United Kingdom and Australia. Internet Interventions-the Application of Information Technology in Mental and Behavioural Health. 2020 Apr;20. PMID: WOS:000550243900011. doi: ARTN 10030810.1016/j.invent.2020.100308.

11. Beaumont R, Walker H, Weiss J, Sofronoff K. Randomized Controlled Trial of a Video Gaming-Based Social Skills Program for Children on the Autism Spectrum. Journal of Autism and Developmental Disorders. 2021 October;51(10):3637–50. PMID: 2007728197. doi: 10.1007/s10803-020-04801-z.

12. Berg M, Rozental A, de Brun Mangs J, Näsman M, Strömberg K, Viberg L, et al. The Role of Learning Support and Chat-Sessions in Guided Internet-Based Cognitive Behavioral Therapy for Adolescents With Anxiety: A Factorial Design Study. Frontiers in Psychiatry. 2020 2020–June–10;Volume 11 - 2020. doi: 10.3389/fpsyt.2020.00503.

13. Bjureberg J, Sahlin H, Hedman-Lagerlof E, Gratz KL, Tull MT, Jokinen J, et al. Extending research on Emotion Regulation Individual Therapy for Adolescents (ERITA) with nonsuicidal self-injury disorder: open pilot trial and mediation analysis of a novel online version. BMC Psychiatry. 2018 10 11;18(1):326. PMID: 30305103. doi: 10.1186/s12888-018-1885-6.

14. de Bruin EJ, Bögels SM, Oort FJ, Meijer AM. Improvements of adolescent psychopathology after insomnia treatment: results from a randomized controlled trial over 1 year. Journal of child psychology and psychiatry, and allied disciplines. 2018;59(5):509–22. PMID: CN-01959502. doi: 10.1111/jcpp.12834.

15. Dingwall KM, Povey J, Sweet M, Friel J, Shand F, Titov N, et al. Feasibility and Acceptability of the Aboriginal and Islander Mental Health Initiative for Youth App: Nonrandomized Pilot With First Nations Young People. JMIR Hum Factors. 2023 Jun 07;10:e40111. PMID: 37285184. doi: 10.2196/40111.

16. Farmer AP. Adoption of an innovation: The story behind preventive services in a community health centre [Dissertation/Thesis]2007.

17. Geirhos A, Domhardt M, Lunkenheimer F, Temming S, Holl RW, Minden K, et al. Feasibility and potential efficacy of a guided internet- and mobile-based CBT for adolescents and young adults with chronic medical conditions and comorbid depression or anxiety symptoms: a randomized controlled pilot trial. BMC Pediatr. 2022 Jan;22(1):15. PMID: WOS:000749189600001. doi: 10.1186/s12887-022-03134-3.

18. Georen L, Jansson-Frojmark M, Nordenstam L, Andersson G, Olsson NC. Internet-delivered Cognitive Behavioral Therapy for insomnia in youth with autism spectrum disorder: A pilot study. Internet Interv. 2022 Sep;29:100548. PMID: 35651733. doi: 10.1016/j.invent.2022.100548.

19. Gladstone T, Buchholz KR, Fitzgibbon M, Schiffer L, Lee M, Voorhees BWV. Randomized Clinical Trial of an Internet-Based Adolescent Depression Prevention Intervention in Primary Care: Internalizing Symptom Outcomes. Int J Environ Res Public Health. 2020 10 22;17(21):22. PMID: 33105889. doi: 10.3390/ijerph17217736.

20. Jolstedt M, Ljotsson B, Fredlander S, Tedgard T, Hallberg A, Ekeljung A, et al. Implementation of internet-delivered CBT for children with anxiety disorders in a rural area: A feasibility trial. Internet Interv. 2018 Jun;12:121–9. PMID: 30135776. doi: 10.1016/j.invent.2017.11.003.

21. Jolstedt M, Wahlund T, Lenhard F, Ljotsson B, Mataix-Cols D, Nord M, et al. Efficacy and cost-effectiveness of therapist-guided internet cognitive behavioural therapy for paediatric anxiety disorders: a single-centre, single-blind, randomised controlled trial. Lancet Child Adolesc Health. 2018 11;2(11):792–801. PMID: 30241993. doi: 10.1016/s2352-4642(18)30275-x.

22. Khan K, Hollis C, Hall CL, Murray E, Davies EB, Andren P, et al. Fidelity of Delivery and Contextual Factors Influencing Children's Level of Engagement: Process Evaluation of the Online Remote Behavioral Intervention for Tics Trial. J Med Internet Res. 2021 06 21;23(6):e25470. PMID: 34152270. doi: 10.2196/25470.

23. Khanna MS, Kendall PC. Computer-assisted cognitive behavioral therapy for child anxiety: results of a randomized clinical trial. Journal of consulting and clinical psychology. 2010;78(5):737–45. PMID: CN-00772841. doi: 10.1037/a0019739.

24. Kurki M, Anttila M, Koivunen M, Marttunen M, Valimaki M. Nurses' experiences of the use of an Internet-based support system for adolescents with depressive disorders. Inform Health Soc Care. 2018 Sep;43(3):234–47. PMID: 28139155. doi: 10.1080/17538157.2016.1269110.

25. Larsson A, Weineland S, Nissling L, Lilja JL. The Impact of Parental Support on Adherence to Therapist-Assisted Internet-Delivered Acceptance and Commitment Therapy in Primary Care for Adolescents With Anxiety: Naturalistic 12-Month Follow-Up Study. JMIR Pediatrics and Parenting. 2025 Jan 03;8:e59489. PMID: 39752209. doi: 10.2196/59489.

26. Lilja JL, Rupcic Ljustina M, Nissling L, Larsson AC, Weineland S. Youths' and Parents' Experiences and Perceived Effects of Internet-Based Cognitive Behavioral Therapy for Anxiety Disorders in Primary Care: Mixed Methods Study. JMIR Pediatr Parent. 2021 11 01;4(4):e26842. PMID: 34723830. doi: 10.2196/26842.

27. Lincke L, Martin-Doring T, Daunke A, Sadkowiak A, Nolkemper DA, Sproeber-Kolb N, et al. Integration of a Mental Health App (e-MICHI) Into a Blended Treatment of Depression in Adolescents: Single-Group, Naturalistic Feasibility Trial. JMIR Formative Research. 2025 May 01;9:e58427. PMID: 40313202. doi: 10.2196/58427.

28. Mazenc KJ. An investigation of engagement in parent-administered, Internet-delivered cognitive behaviour therapy for childhood anxiety: Intervention usage and subjective experience.DP - 2023. Dissertation Abstracts International Section A: Humanities and Social Sciences. 2023;84(5-A):No Pagination Specified. PMID: 2023-33083-281.

29. Miklowitz DJ, Weintraub MJ, Posta F, Walshaw PD, Frey SJ, Morgan-Fleming GM, et al. Development and Open Trial of a Technology-Enhanced Family Intervention for Adolescents at Risk for Mood Disorders. J Affect Disord. 2021 02 15;281:438–46. PMID: 33360365. doi: 10.1016/j.jad.2020.12.012.

30. Miller AK, Ely SL, Barber Garcia BN, Richardson P, Cunningham NR. Engagement during a Mixed In-Person and Remotely Delivered Psychological Intervention for Youth with Functional Abdominal Pain Disorders and Anxiety. Children (Basel). 2021 Sep 02;8(9):02. PMID: 34572207. doi: 10.3390/children8090775.

31. Molleda L, Bahamon M, St George SM, Perrino T, Estrada Y, Correa Herrera D, et al. Clinic Personnel, Facilitator, and Parent Perspectives of eHealth Familias Unidas in Primary Care. J Pediatr Health Care. 2017 May – Jun;31(3):350–61. PMID: 28012799. doi: 10.1016/j.pedhc.2016.11.001.

32. Nordh M, Vigerland S, Ost LG, Ljotsson B, Mataix-Cols D, Serlachius E, et al. Therapist-guided internet-delivered cognitive-behavioural therapy supplemented with group exposure sessions for adolescents with social anxiety disorder: a feasibility trial. BMJ Open. 2017 Dec 14;7(12):e018345. PMID: 29247101. doi: 10.1136/bmjopen-2017-018345.

33. Nordh M, Wahlund T, Jolstedt M, Sahlin H, Bjureberg J, Ahlen J, et al. Therapist-Guided Internet-Delivered Cognitive Behavioral Therapy vs Internet-Delivered Supportive Therapy for Children and Adolescents With Social Anxiety Disorder: A Randomized Clinical Trial. JAMA Psychiatry. 2021 Jul 1;78(7):705–13. PMID: 33978699. doi: 10.1001/jamapsychiatry.2021.0469.

34. Rautio D, Andren P, Gumpert M, Jolstedt M, Jassi A, Krebs G, et al. Therapist-guided, Internet-delivered cognitive behaviour therapy for adolescents with body dysmorphic disorder: A feasibility trial with long-term follow-up. Internet Interv. 2023 Dec;34:100688. PMID: 38034863. doi: 10.1016/j.invent.2023.100688.

35. Salloum A, Crawford EA, Lewin AB, Storch EA. Consumers' and providers' perceptions of utilizing a computer-assisted cognitive behavioral therapy for childhood anxiety. Behav. 2015 Jan;43(1):31–41. PMID: 23886438. doi: 10.1017/s1352465813000647.

36. Sandín B, García-Escalera J, Valiente RM, Espinosa V, Chorot P. Clinical Utility of an Internet-Delivered Version of the Unified Protocol for Transdiagnostic Treatment of Emotional Disorders in Adolescents (iUP-A): A Pilot Open Trial. International Journal of Environmental Research and Public Health. 2020 Nov;17(22):17. PMID: WOS:000594315300001. doi: 10.3390/ijerph17228306.

37. Silfvernagel K, Gren-Landell M, Emanuelsson M, Carlbring P, Andersson G. Individually tailored internet-based cognitive behavior therapy for adolescents with anxiety disorders: A pilot effectiveness study. Internet Interv. 2015 September 01;2(3):297–302. PMID: 606623223. doi: 10.1016/j.invent.2015.07.002.

38. Silk JS, Pramana G, Sequeira SL, Lindhiem O, Kendall PC, Rosen D, et al. Using a smartphone app and clinician portal to enhance brief cognitive behavioral therapy for childhood anxiety disorders. Behav. 2020 Jan;51(1):69–84. PMID: 2019-41438-001. doi: 10.1016/j.beth.2019.05.002.

39. Spence SH, Donovan CL, March S, Gamble A, Anderson R, Prosser S, et al. Online CBT in the treatment of child and adolescent anxiety disorders: Issues in the development of BRAVE-ONLINE and two case illustrations. Behavioural and Cognitive Psychotherapy. 2008 July;36(4):411–30. PMID: 352165018. doi: 10.1017/s135246580800444x.

40. Srivastava P, Mehta M, Sagar R, Ambekar A. Smartteen- a computer assisted cognitive behavior therapy for Indian adolescents with depression- a pilot study. Asian J Psychiatr. 2020 April;50(no pagination). PMID: 2005075842. doi: 10.1016/j.ajp.2020.101970.

41. Stallard P, Richardson T, Velleman S, Attwood M. Computerized CBT (Think, Feel, Do) for Depression and Anxiety in Children and Adolescents: Outcomes and Feedback from a Pilot Randomized Controlled Trial. Behavioural and Cognitive Psychotherapy. 2011 May;39(3):273–84. PMID: WOS:000289726000002. doi: 10.1017/s135246581000086x.

42. Stasiak K, Merry SN, Frampton C, Moor S. Delivering solid treatments on shaky ground: Feasibility study of an online therapy for child anxiety in the aftermath of a natural disaster. Psychother. 2018 07;28(4):643–53. PMID: 27781568. doi: 10.1080/10503307.2016.1244617.

43. Stjerneklar S, Hougaard E, Nielsen AD, Gaardsvig MM, Thastum M. Internet-based cognitive behavioral therapy for adolescents with anxiety disorders: A feasibility study. Internet Interv. 2018 Mar;11:30–40. PMID: WOS:000457134500004. doi: 10.1016/j.invent.2018.01.001.

44. Stjerneklar S, Hougaard E, McLellan LF, Thastum M. A randomized controlled trial examining the efficacy of an internet-based cognitive behavioral therapy program for adolescents with anxiety disorders. PLoS ONE. 2019;.14(9):ArtID e0222485. PMID: 2019-57199-001. doi: https://dx.doi.org/10.1371/journal.pone.0222485.

45. Topooco N, Berg M, Johansson S, Liljethörn L, Radvogin E, Vlaescu G, et al. Chat- and internet-based cognitive–behavioural therapy in treatment of adolescent depression: randomised controlled trial. BJPsych Open. 2018;4(4):199–207. doi: 10.1192/bjo.2018.18.

46. Topooco N, Bylehn S, Dahlstrom Nysater E, Holmlund J, Lindegaard J, Johansson S, et al. Evaluating the Efficacy of Internet-Delivered Cognitive Behavioral Therapy Blended With Synchronous Chat Sessions to Treat Adolescent Depression: Randomized Controlled Trial. J Med Internet Res. 2019 11 01;21(11):e13393. PMID: 31682572. doi: 10.2196/13393.

47. Van Voorhees BW, Fogel J, Pomper BE, Marko M, Reid N, Watson N, et al. Adolescent Dose and Ratings of an Internet-Based Depression Prevention Program: A Randomized Trial of Primary Care Physician Brief Advice versus a Motivational Interview. J. 2009;9(1):1–19. PMID: 20694059.

48. Eisen JC, Marko-Holguin M, Fogel J, Cardenas A, Bahn M, Bradford N, et al. Pilot Study of Implementation of an Internet-Based Depression Prevention Intervention (CATCH-IT) for Adolescents in 12 US Primary Care Practices: Clinical and Management/Organizational Behavioral Perspectives. The Primary Care Companion to CNS Disorders. 2013;15(6). PMID: 24800110. doi: 10.4088/PCC.10m01065.

49. Van Voorhees BW, Watson N, Bridges JF, Fogel J, Galas J, Kramer C, et al. Development and pilot study of a marketing strategy for primary care/internet-based depression prevention intervention for adolescents (the CATCH-IT intervention). Primary Care Companion to the Journal of Clinical Psychiatry. 2010;12(3). PMID: 20944776. doi: 10.4088/PCC.09m00791blu.

50. Vigerland S, Fredlander S, Aspvall K, Jolstedt M, Lenhard F, Mataix-Cols D, et al. Effectiveness of internet-delivered cognitive behavioural therapy for anxiety and obsessive-compulsive disorders within routine clinical care in rural Sweden. Internet Interventions. 2024 Jun;36:100738. PMID: 38617387. doi: 10.1016/j.invent.2024.100738.

51. Voerman JS, Remerie S, Westendorp T, Timman R, Busschbach JJ, Passchier J, et al. Effects of a Guided Internet-Delivered Self-Help Intervention for Adolescents With Chronic Pain. The journal of pain. 2015;16(11):1115–26. PMID: CN-01137503. doi: 10.1016/j.jpain.2015.07.011.

52. Waite P, Marshall T, Creswell C. A randomized controlled trial of internet-delivered cognitive behaviour therapy for adolescent anxiety disorders in a routine clinical care setting with and without parent sessions. Child Adolesc Ment Health. 2019 Sep;24(3):242–50. PMID: 32677216. doi: 10.1111/camh.12311.

53. Weineland S, Ribbegardh R, Kivi M, Bygdell A, Larsson A, Vernmark K, et al. Transitioning from face-to-face treatment to iCBT for youths in primary care - therapists' attitudes and experiences. Internet Interv. 2020 Dec;22:100356. PMID: 33318951. doi: 10.1016/j.invent.2020.100356.

54. Weintraub MJ, Ichinose MC, Zinberg J, Done M, Morgan-Fleming GM, Wilkerson CA, et al. App-enhanced transdiagnostic CBT for adolescents with mood or psychotic spectrum disorders. J Affect Disord. 2022 08 15;311:319–26. PMID: 35594972. doi: 10.1016/j.jad.2022.05.094.

55. Wickberg F, Lenhard F, Aspvall K, Serlachius E, Andren P, Johansson F, et al. Feasibility of internet-delivered cognitive-behavior therapy for obsessive-compulsive disorder in youth with autism spectrum disorder: a clinical benchmark study. Internet Interv. 2022;28. PMID: CN-02375952. doi: 10.1016/j.invent.2022.100520.

56. Long HA, French DP, Brooks JM. Optimising the value of the critical appraisal skills programme (CASP) tool for quality appraisal in qualitative evidence synthesis. Research Methods in Medicine & Health Sciences. 2020;1(1):31–42. doi: 10.1177/2632084320947559.

57. Viswanathan M, Patnode CD, Berkman ND, Bass EB, Chang S, Hartling L, et al. Recommendations for assessing the risk of bias in systematic reviews of health-care interventions. Journal of Clinical Epidemiology. 2018 2018/05/01/;97:26–34. doi: https://doi.org/10.1016/j.jclinepi.2017.12.004.

58. Gelman A, Vehtari A, Simpson D, Margossian CC, Carpenter B, Yao Y, et al. Bayesian workflow. arXiv preprint arXiv:201101808. 2020. doi: https://doi.org/10.48550/arXiv.2011.01808.

59. Rognli EW, Zahl-Olsen R, Rekdal SS, Hoffart A, Bertelsen TB. Editorial perspective: Bayesian statistical methods are useful for researchers in child and adolescent mental health. J Child Psychol Psychiatry. 2023 Feb;64(2):339–42. PMID: 35818323. doi: 10.1111/jcpp.13662.

60. Reis DJ, Kaizer AM, Kinney AR, Bahraini NH, Holliday R, Forster JE, et al. A Practical Guide to Random-Effects Bayesian Meta-Analyses With Application to the Psychological Trauma and Suicide Literature. Psychol Trauma-Us. 2023 Jan;15(1):121–30. PMID: WOS:000827803000001. doi: 10.1037/tra0001316.

61. van Buuren S, Groothuis-Oudshoorn K. mice: Multivariate Imputation by Chained Equations in R. Journal of Statistical Software. 2011 12/12;45(3):1 – 67. doi: 10.18637/jss.v045.i03.

62. Bürkner P-C. brms: An R Package for Bayesian Multilevel Models Using Stan. Journal of Statistical Software. 2017 08/29;80(1):1 – 28. doi: 10.18637/jss.v080.i01.
