# Supplementary material for: Barriers to and Facilitators of Implementation of Internet-Delivered Therapist-Guided Therapy in Child and Adolescent Mental Health Services: Systematic Review and Bayesian Meta-Analysis
Source: J Med Internet Res. 2025 Dec 22;27:e83543. doi: 10.2196/83543 (PMC12721491; doi:10.2196/83543)
Supplement: Multimedia Appendix 5 [file jmir-v27-e83543-s005.docx]

Appendix 5 – Prior specifications

Table 1. Specifications of weakly informative priors for intercepts and between-study heterogeneity hyperparameters used in the Bayesian random-effects meta-analyses

| Parameters | Prior distribution | Reasoning |
| --- | --- | --- |
| Mean Dropout (log odds) | Normal (-0.524, 1.5) | Weakly informative prior. When transformed to probability scale no negative values, less likely in the extremes, more plausible with less than 50% dropout rate than more than 50%. |
| Mean program completion proportion (for patients) | Normal (0.6, 0.2) | Weakly informative prior. Assumed large variation in how much of the program is completed due to variability in program type and context and therapist support. Assumed that the completion rate is less likely in the extremes, is normally distributed, and that it is more probable to complete more than half than less. |
| Mean Therapist Time per Patient Per Week | Normal (0.5, 0.15) | Weakly informative prior. Assumed large variation in study program and amount of therapist support, but some form of therapist support. Assumed that time spent is less than normal therapy. |
| Mean CSQ-8 patient score (satisfaction measure from 8-32) | Normal (20, 4) | Weakly informative prior. Assumed ceiling effect of self-report scores, with large variation allowing for lower scores. Assumed normally distributed. |
| Mean Satisfaction Rate | Normal (0.55, 0.2) | Weakly informative prior. Assumed ceiling effect for satisfaction report, but not impossible to be below 50%. |
| Tau (between study heterogeneity hyperparameter/SD for random effects) | Half Cauchy/log Normal (0, x) | Very weakly informative prior: Assumed positive value, probability mass decreasing away from 0, with scale specific prior assumptions for sd for each model:  For dropout: 1-81% sd is probable. Log Normal(-2.5, 0.75)  For completion rate: half of the range of the prior for sd is probable (40%), (0, 0.2).  Therapist time: 30 min (0, 15).  CSQ: 8 points (0, 4).  Satisfaction rate: 40% (0, 0.2). |
| Regression coefficient for time and design | Normal (0, x) | Weakly informative prior: It is safe to assume that the probability of time and design having an effect could be positive or negative, but it is assumed that one factor alone will have less than 50% impact, and the range will differ for the sd for each scale:  For dropout: 0-40% sd is probable, not excluding higher than 40%. (0, 0.2) (on log scale: normal(-0.75, 1.5))  For completion rate: half of the range of the prior for sd is probable (40%), (0, 0.2). |
