# Supplementary material for: Barriers to and Facilitators of Implementation of Internet-Delivered Therapist-Guided Therapy in Child and Adolescent Mental Health Services: Systematic Review and Bayesian Meta-Analysis
Source: J Med Internet Res. 2025 Dec 22;27:e83543. doi: 10.2196/83543 (PMC12721491; doi:10.2196/83543)
Supplement: Multimedia Appendix 6 [file jmir-v27-e83543-s006.docx]

Appendix 6 – Model diagnostics of convergence


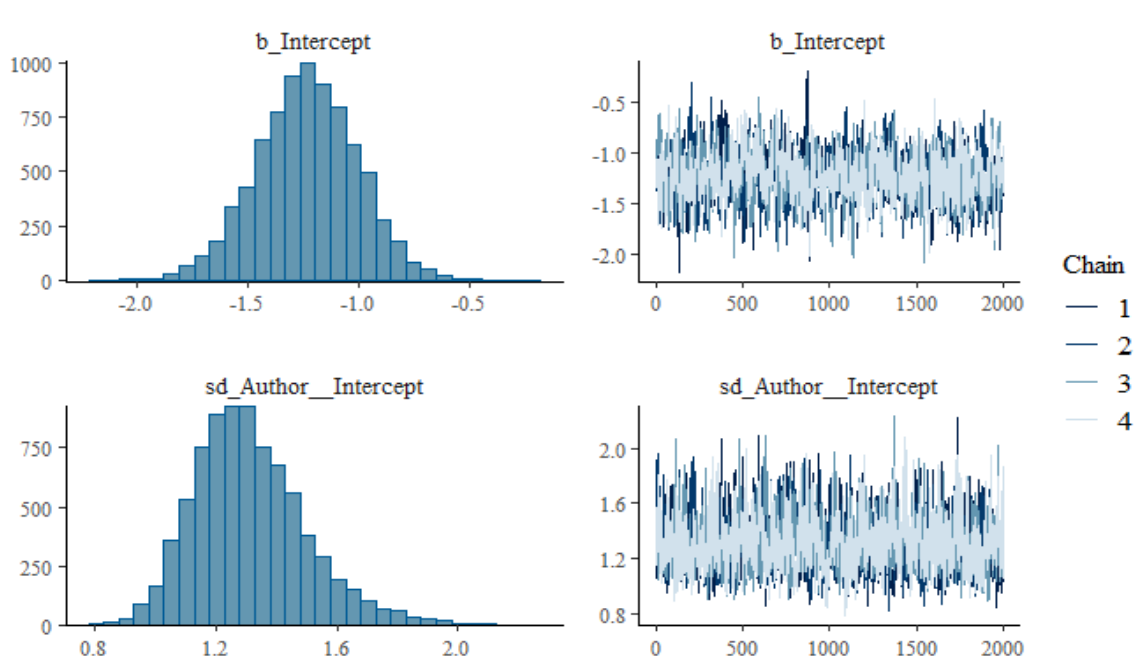


Figure 1. Summary plot of posterior density and convergence of complete cases Bayesian random-effects meta-analysis model on log odds scale (b_Intercept: mean patient therapy dropout, sd_Author_Intercept: between-study heterogeneity (tau)) (N=46)


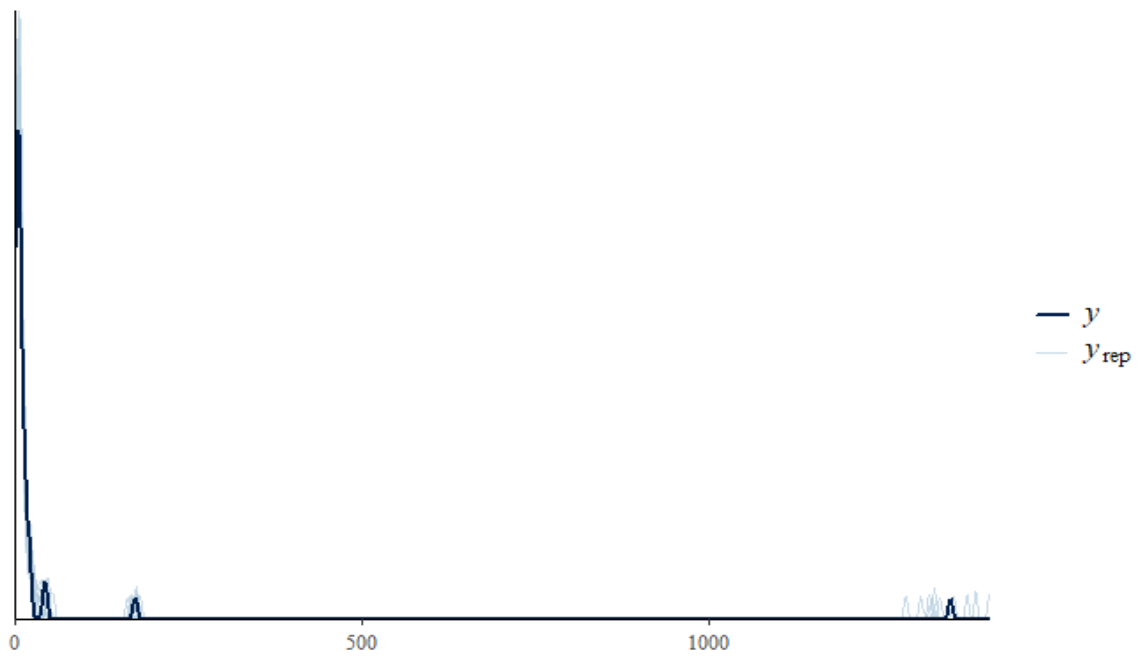


Figure 2. Posterior predictive check plot of multiple imputation Bayesian random-effects meta-analysis model of mean patient therapy dropout (log odds) (Y: observed data, Yrep: simulated data from the posterior distribution)


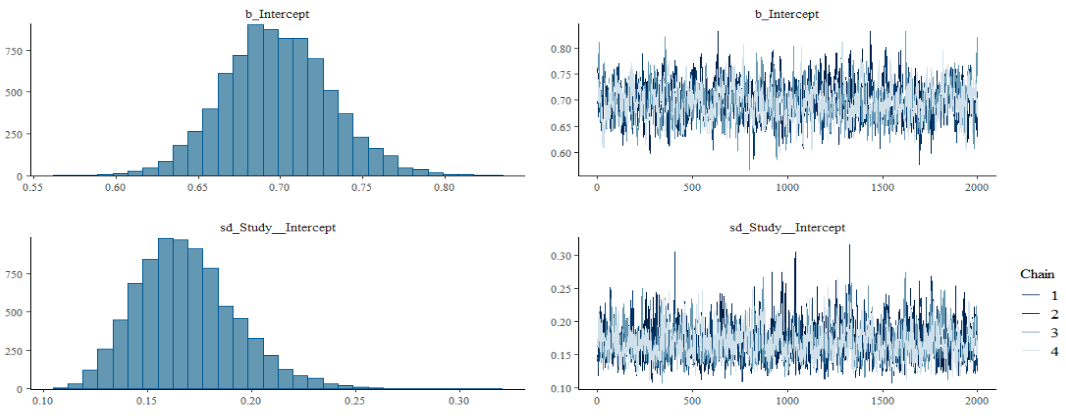


Figure 3. Summary plot of posterior density and convergence of complete cases Bayesian random-effects meta-analysis model (b_Intercept: mean patient program completion, sd_Study_Intercept: between-study heterogeneity (tau)) (N=31)


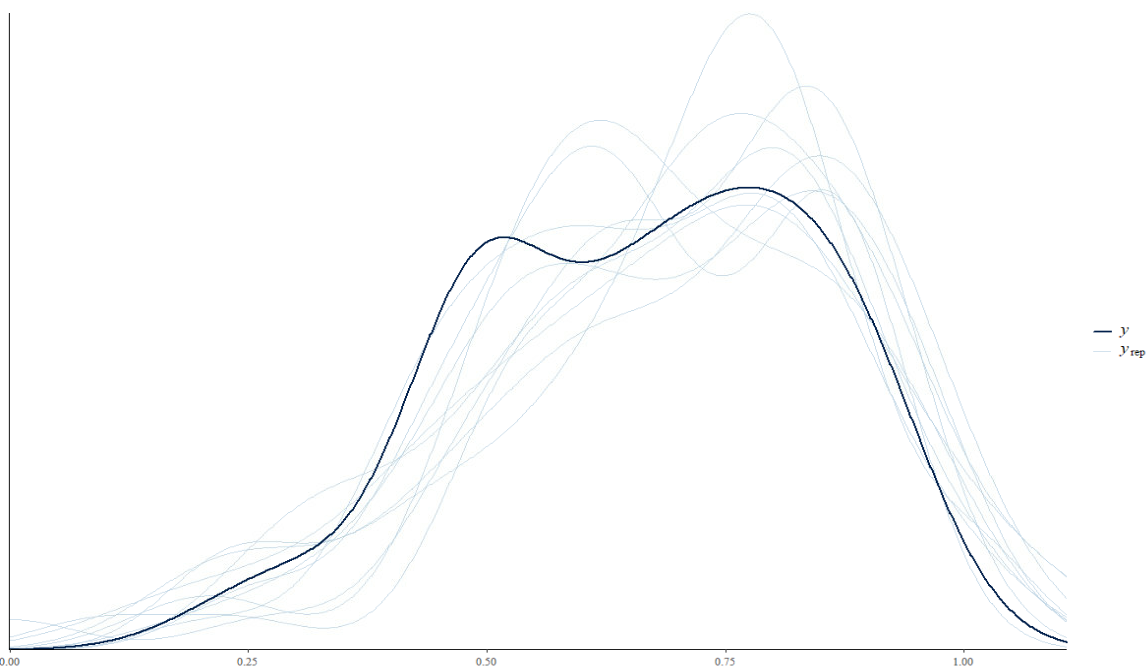


Figure 4. Posterior predictive check plot of multiple imputation Bayesian random-effects meta-analysis model of mean proportion of program completed by patients (Y: observed data, Yrep: simulated data from the posterior distribution)


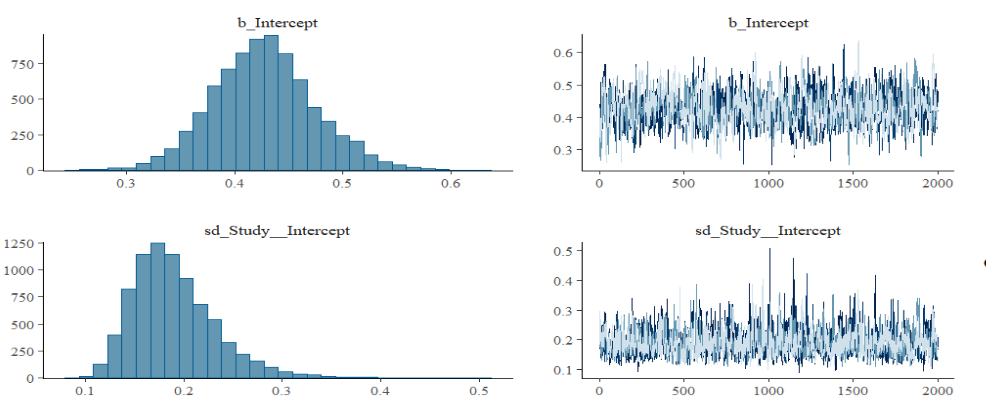


Figure 5. Summary plot of posterior density and convergence of complete cases Bayesian random-effects meta-analysis model (b_Intercept: mean therapist time per patient per week (hours), sd_Study_Intercept: between-study heterogeneity (tau)) (N=15)


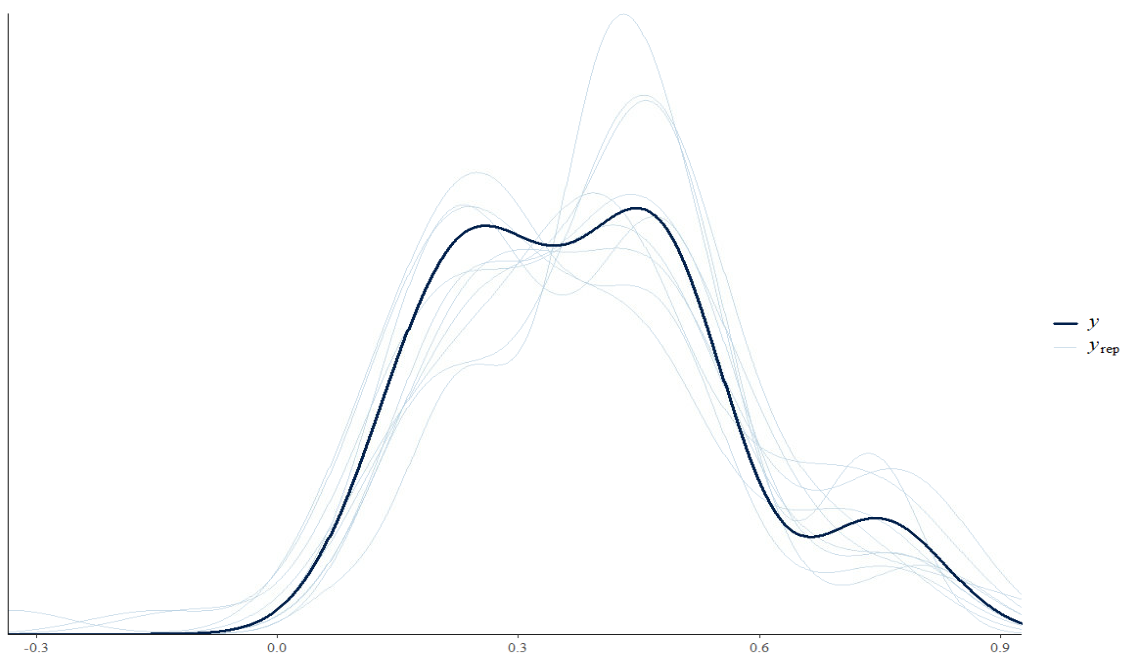


Figure 6. Posterior predictive check plot of multiple imputation Bayesian random-effects meta-analysis model of mean therapist time per patient per week (hours) (Y: observed data, Yrep: simulated data from the posterior distribution)


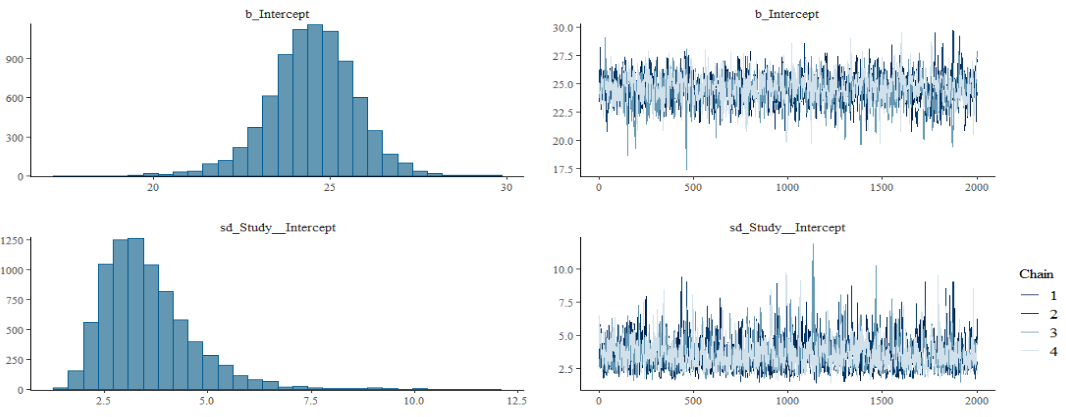


Figure 7. Summary plot of posterior density and convergence of complete cases Bayesian random-effects meta-analysis model (b_Intercept: mean patient satisfaction level from the client satisfaction questionnaire (CSQ-8, 8-31), sd_Study_Intercept: between-study heterogeneity (tau)) (N=9)


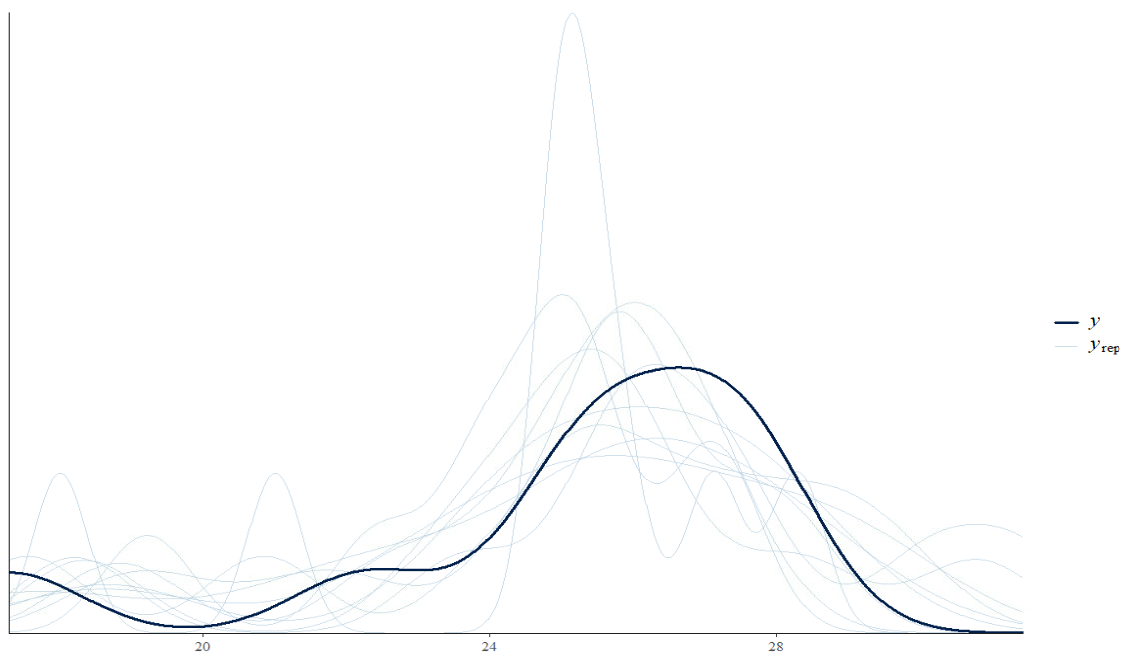


Figure 8. Posterior predictive check plot of multiple imputation Bayesian random-effects meta-analysis model of mean patient satisfaction level from the client satisfaction questionnaire (CSQ-8, 8-31) (Y: observed data, Yrep: simulated data from the posterior distribution)


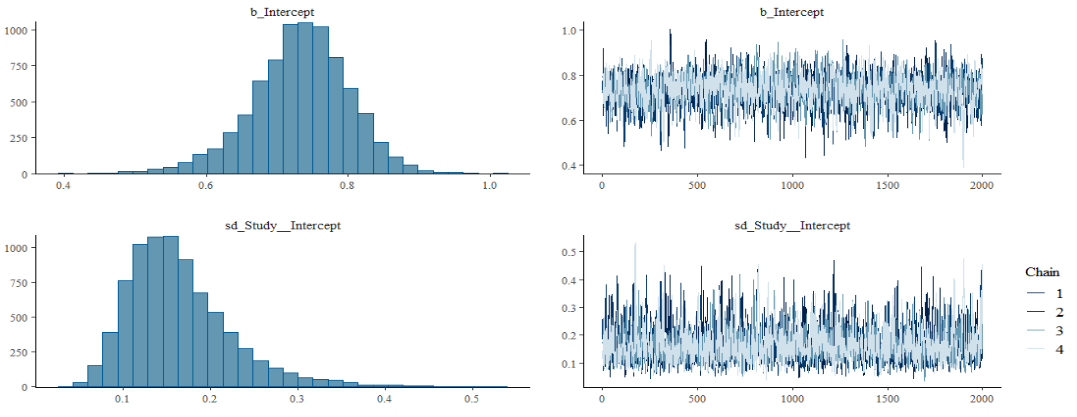


Figure 9. Summary plot of posterior density and convergence of complete cases Bayesian random-effects meta-analysis model (b_Intercept: mean patient satisfaction rating, sd_Study_Intercept: between-study heterogeneity (tau)) (N=9)


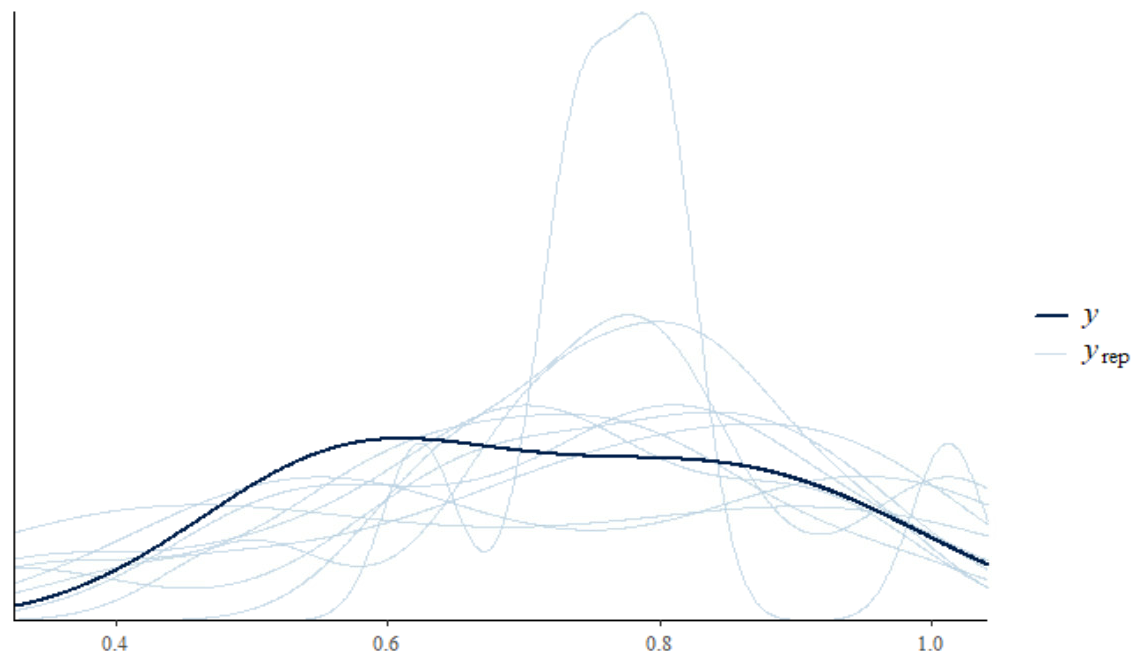


Figure 10. Posterior predictive check plot of multiple imputation Bayesian random-effects meta-analysis model of mean patient rated satisfaction (Y: observed data, Yrep: simulated data from the posterior distribution)
