# Supplementary material for: Barriers to and Facilitators of Implementation of Internet-Delivered Therapist-Guided Therapy in Child and Adolescent Mental Health Services: Systematic Review and Bayesian Meta-Analysis
Source: J Med Internet Res. 2025 Dec 22;27:e83543. doi: 10.2196/83543 (PMC12721491; doi:10.2196/83543)
Supplement: Multimedia Appendix 7 [file jmir-v27-e83543-s007.docx]

Appendix 7 - Summary of risk of bias assessments

Table 1. Summary of risk of bias assessments of aggregated implementation outcomes extracted from non-randomized studies (ROBINS-I V2) included in the review of implementation of internet-delivered, therapist-guided therapy in Child and Adolescent Mental Health Services

| **The Risk Of Bias In Non-randomized Studies – of Interventions, Version 2 (ROBINS-I V2)** | | | | | |
| --- | --- | --- | --- | --- | --- |
| Study | Outcome assessed for risk of bias | 5. Bias due to missing data | 6. Bias in measurement of outcome | 7. Bias in selection of the reported results | Overall bias |
| Alvarez-Jiminez et al 2025 | Dropout rate | low | low | low | low |
|  | Satisfaction rate | critical | low | low | critical |
| Aspvall et al 2018 | Dropout rate | low | low | low | low |
|  | Completion rate | low | low | low | low |
|  | Therapist time | low | low | low | low |
| Aspvall et al 2020 | Dropout rate | low | low | low | low |
|  | Completion rate | low | low | low | low |
|  | Therapist time | low | low | low | low |
| Bjureberg et al 2018 | Dropout rate | low | low | low | low |
|  | Completion rate | low | low | low | low |
|  | Satisfaction rate | low | low | low | low |
|  | Satisfaction scale | low | low | low | low |
| Jolstedt et al 2018 | Dropout rate | low | low | low | low |
|  | Completion rate | low | low | low | low |
| Larsson et al 2025 | Dropout rate | low | low | low | low |
|  | Completion rate | low | low | low | low |
| Lincke et al 2025 | Dropout rate | low | low | low | low |
|  | Completion rate | low | low | low | low |
| Midgley et al 2021 | Dropout rate | low | low | low | low |
|  | Satisfaction rate | low | low | low | low |
| Miklowitz et al 2021 | Dropout rate | low | low | low | low |
|  | Completion rate | low | low | low | low |
| Nordh et al 2017 | Dropout rate | low | low | low | low |
|  | Completion rate | low | low | low | low |
| Rautio et al 2023 | Dropout rate | low | low | low | low |
| Sandin et al 2020 | Dropout rate | low | low | low | low |
|  | Completion rate | low | low | low | low |
| Silk et al 2020 | Dropout rate | low | low | low | low |
|  | Satisfaction rate | low | low | low | low |
|  | Satisfaction scale | low | low | low | low |
| Silvernagel et al 2015 | Dropout rate | low | low | low | low |
|  | Completion rate | low | low | low | low |
| Stasiak et al 2018 | Dropout rate | low | low | low | low |
|  | Completion rate | low | low | low | low |
| Voerman et al 2015 | Dropout rate | low | low | low | low |
|  | Satisfaction rate | critical | low | low | critical |
| Vigerland et al 2024 | Dropout rate | low | low | low | low |
|  | Completion rate | low | low | low | low |
|  | Satisfaction rate | low | low | low | low |
|  | Therapist time | low | low | low | low |
| Weintraubet al 2022 | Dropout rate | low | low | low | low |
|  | Completion rate | low | low | low | low |
| Wickberg et al 2022 | Dropout rate | low | low | low | low |
|  | Completion rate | low | low | low | low |
|  | Satisfaction scale | low | low | low | low |
|  | Therapist time | low | low | low | low |

Table 2. Summary of risk of bias assessments of aggregated implementation outcomes extracted from randomized trials (RoB 2) included in the review of implementation of internet-delivered, therapist-guided therapy in Child and Adolescent Mental Health Services

| Study | Outcome assed for risk of bias | 3. Bias due to missing data | 4. Bias in measurement of outcome | 5. Bias in selection of reported results | Overall bias |
| --- | --- | --- | --- | --- | --- |
| Andren et al 2019 | Dropout rate | low | low | low | low |
|  | Completion rate | low | low | low | low |
|  | Therapist time | low | low | low | low |
| Andren et al 2024 | Dropout rate | low | some concerns | low | some concerns |
|  | Completion rate | low | low | low | low |
|  | Satisfaction scale | low | low | low | low |
|  | Therapist time | low | low | low | low |
| Aspvall et al 2021 | Dropout rate | low | low | low | low |
|  | Completion rate | low | low | low | low |
|  | Satisfaction scale | low | low | low | low |
|  | Therapist time | low | low | low | low |
| Beaumont et al 2021 | Dropout rate | low | low | low | low |
| Berg et al 2020 | Dropout rate | low | low | low | low |
|  | Completion rate | low | low | low | low |
|  | Therapist time | low | low | low | low |
| De Bruin et al 2018 | Dropout rate | low | low | low | low |
| Geirhos et al 2022 | Dropout rate | low | low | low | low |
|  | Completion rate | low | low | low | low |
|  | Satisfaction scale | low | low | low | low |
|  | Satisfaction rate | low | low | low | low |
| Gladstone et al 2020 | Dropout rate | low | low | low | low |
|  | Completion rate | low | low | some concerns | some concerns |
| Jolstedt et al 2018 | Dropout rate | low | low | low | low |
|  | Completion rate | low | low | low | low |
|  | Satisfaction rate | low | low | low | low |
|  | Therapist time | low | low | low | low |
| Khanna & Kendall 2010 | Dropout rate | low | low | low | low |
|  | Satisfaction scale | low | low | low | low |
| Nordh et al 2021 | Dropout rate | low | low | low | low |
|  | Completion rate | low | low | low | low |
|  | Satisfaction scale | low | low | low | low |
|  | Therapist time | low | low | low | low |
| Srivastava et al 2020 | Dropout rate | low | low | low | low |
|  | Completion rate | low | low | low | low |
|  | Therapist time | low | low | low | low |
| Stallard et al 2011 | Dropout rate | low | low | low | low |
| Stjerneklar et al 2019 | Dropout rate | low | low | low | low |
|  | Completion rate | low | low | low | low |
|  | Satisfaction rate | low | low | low | low |
| Topooco et al 2018 | Dropout rate | low | low | low | low |
|  | Completion rate | low | low | low | low |
|  | Therapist time | low | low | low | low |
| Topooco et al 2019 | Dropout rate | low | low | low | low |
|  | Completion rate | low | low | low | low |
|  | Therapist time | low | low | low | low |
| Van Voorhees et al 2009 | Dropout rate | low | low | some concerns | some concerns |
|  | Completion rate | low | low | low | low |
| Waite et al 2019 | Dropout rate | low | low | low | low |
|  | Satisfaction rate | critical | critical | critical | critical |

| Study | 1. Was there a clear statement of the aims of the research? | 2. Is a qualitative methodology appropriate? | 3. Was the research design appropriate to address the aims of the research? | 4. Was the recruitment strategy appropriate to the aims of the research? | 5. Was the data collected in a way that addressed the research issue? | 6. Has the relationship between researcher and participants been adequately considered? | 7. Have ethical issues been taken into consideration? | 8. Was the data analysis sufficiently rigorous? | 9. Is there a clear statement of findings? | 10. How valuable is the research? |
| --- | --- | --- | --- | --- | --- | --- | --- | --- | --- | --- |
| Farmer 2007 | Yes | Yes | Yes | Yes | Yes | Yes | Yes | Yes | Yes | Yes |
| Stjerneklar et al 2018 | Yes | Yes | Can't tell | Yes | Yes | No | Yes | No | Yes | Yes |
| Lilja et al 2021 | Yes | Yes | Yes | Yes | Yes | Yes | Yes | Yes | Yes | Yes |
| Georen et al 2022 | Yes | Yes | Yes | Yes | Yes | No | Yes | Yes | Yes | Yes |
| Weineland et al 2020 | Yes | Yes | Yes | Yes | Yes | No | Yes | Yes | Yes | Yes |
| Mazenc 2023 | Yes | Yes | Yes | Yes | Yes | Yes | Yes | Yes | Yes | Yes |
| Spence et al 2008 | Yes | Yes | Can't tell | Can't tell | No | No | No | No | Yes | Can't tell |
| Dingwall et al 2023 | Yes | Yes | Yes | Yes | Can't tell | No | Yes | No | Yes | Yes |
| Kahn et al 2021 | Yes | Yes | Yes | Yes | Yes | Can't tell | Yes | Yes | Yes | Yes |
| Kurki et al 2018 | Yes | Yes | Yes | Yes | Yes | No | Yes | Yes | Yes | Yes |
| Miller et al 2021 | Yes | Can't tell | Can't tell | Can't tell | Can't tell | No | Can't tell | Can't tell | Yes | Yes |
| Molleda et al 2017 | Yes | Yes | Yes | Yes | Yes | Yes | Yes | Yes | Yes | Yes |

Table 3. Summary of quality assessments of qualitative studies (CASP for qualitative studies) included in the review of implementation of internet-delivered, therapist-guided therapy in Child and Adolescent Mental Health Servic
