# Supplementary material for: Barriers to and Facilitators of Implementation of Internet-Delivered Therapist-Guided Therapy in Child and Adolescent Mental Health Services: Systematic Review and Bayesian Meta-Analysis
Source: J Med Internet Res. 2025 Dec 22;27:e83543. doi: 10.2196/83543 (PMC12721491; doi:10.2196/83543)
Supplement: Multimedia Appendix 8 [file jmir-v27-e83543-s008.docx]

Appendix 8 – Forest and Funnel plots


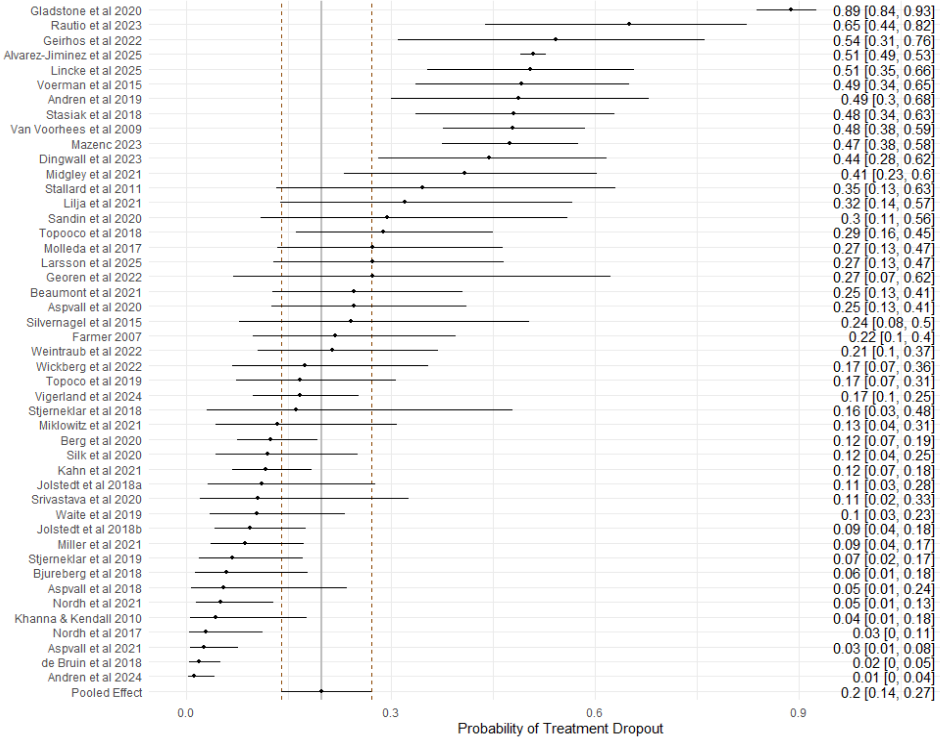


Figure 1. Forest plot of mean patient therapy dropout probability (Credible Interval, CI), from Bayesian random-effects meta-analysis model of reviewed studies on implementation of internet-delivered, therapist-supported therapy in child and mental health services across various diagnoses and research sites (2007-2025) ^1^

^1^ f(binomial) = Y|trials(n) ~ 1 + (1|Study)

Y ~ Normal(-0.524, 1.5)

Tau ~ logNormal(-2.5, 0.75)

^
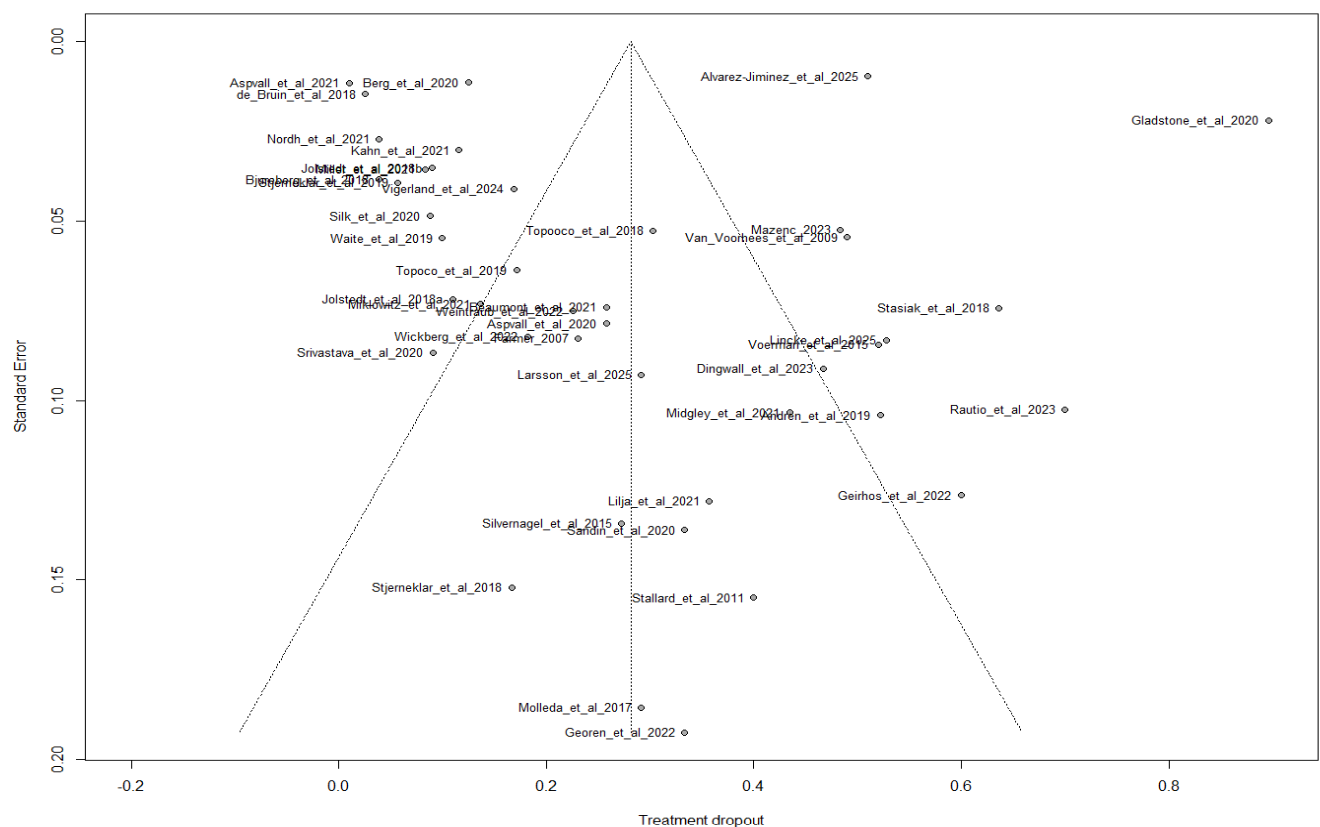
^

Figure 2. Funnel plot of mean treatment dropout rate of reviewed studies on implementation of internet-delivered, therapist-supported therapy in child and mental health services across various diagnoses and research sites (2007-2025)^1^

^1^Eggers regression test for funnel plot asymmetry: b = 0.13 (CI: 0.02, 0.23), p < 0.01


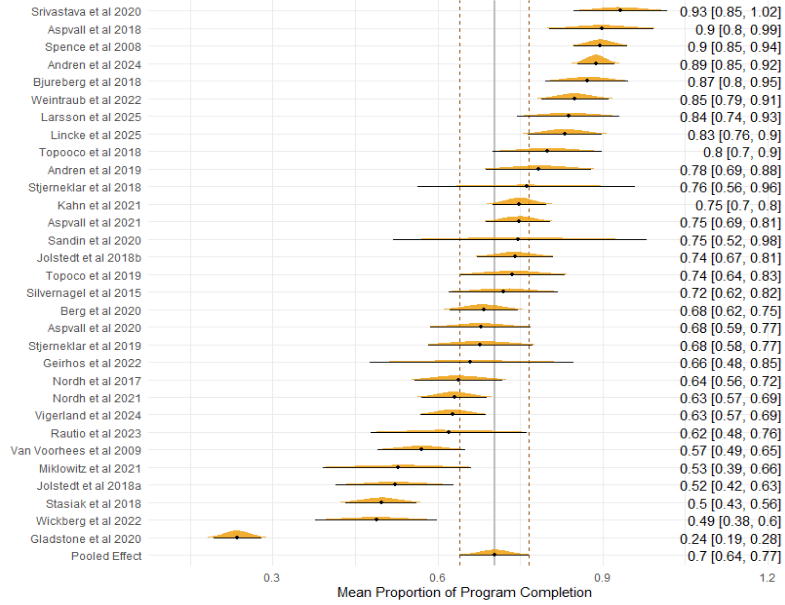
 Figure 3. Forest plot of mean proportion of program completed by patients (Credible Interval, CI), from Bayesian random-effects meta-analysis model of reviewed studies on implementation of internet-delivered, therapist-supported therapy in child and mental health services across various diagnoses and research sites (2007-2025), stratified by study design.^1^

^1^ f= Y|seY ~ 1 + (1|Study)

Y ~ Normal(0.6, 0.2)

Tau ~ half-Cauchy(0, 0.2)


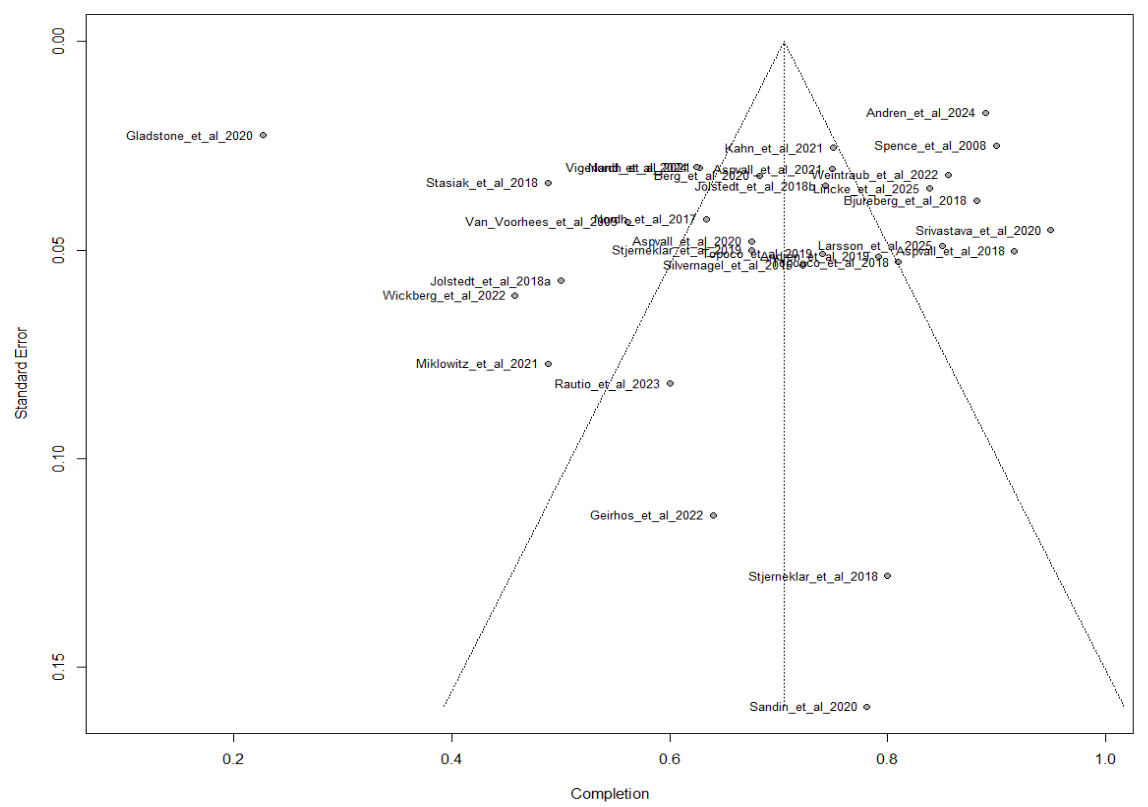
 Figure 4. Funnel plot of mean proportion of patients’ program completion of reviewed studies on implementation of internet-delivered, therapist-supported therapy in child and mental health services across various diagnoses and research sites (2007-2025)^1^

^1^Regression test for funnel plot asymmetry: b= 0.71 (CI: 0.59, 0.84), p > 0.05


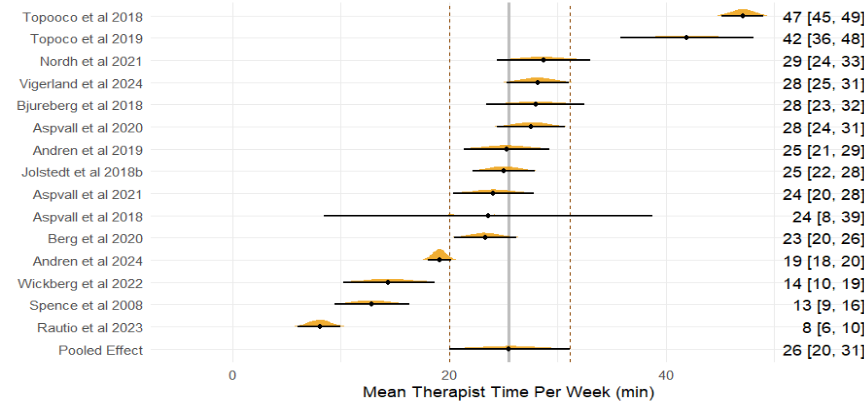
 Figure 5. Forest plot of mean therapist time per patient per week (Credible Interval, CI), from Bayesian random-effects meta-analysis model of reviewed studies on implementation of internet-delivered, therapist-supported therapy in child and mental health services across various diagnoses and research sites (2007-2025), stratified by study design.^1^

^1^ f= Y|seY ~ 1 + (1|Study)

Y ~ Normal (30, 10)

Tau ~ half-Cauchy(0, 10)


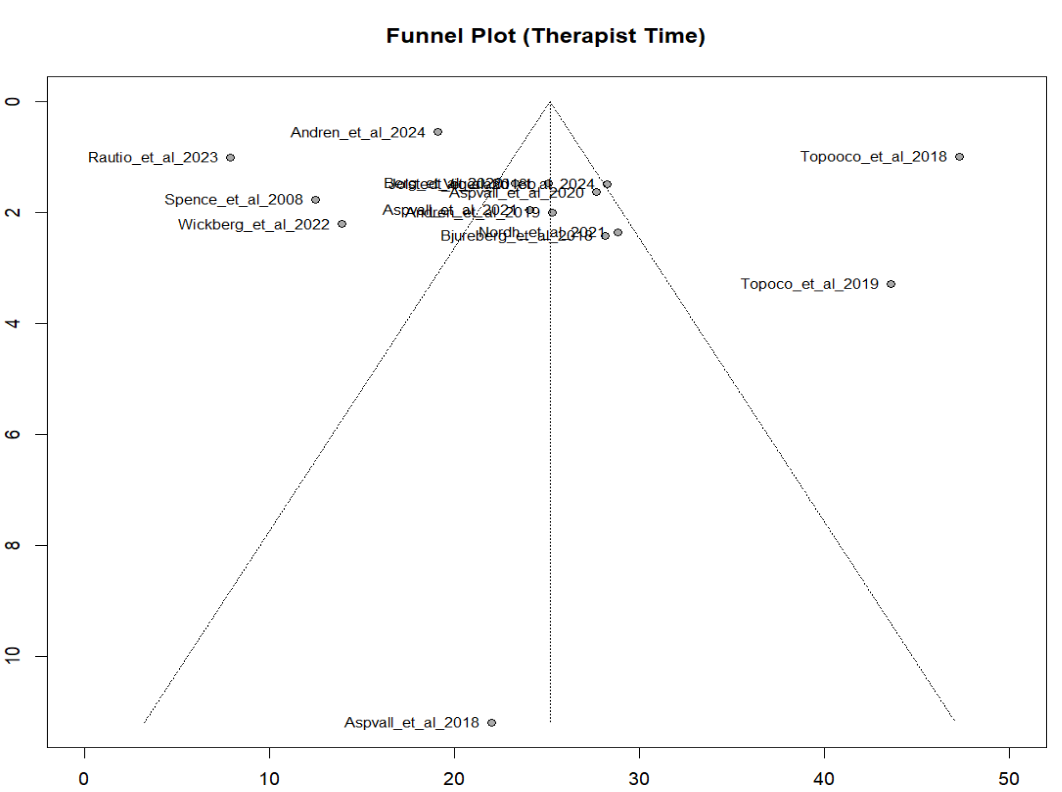
 Figure 6. Funnel plot of mean therapist time per patient per week of reviewed studies on implementation of internet-delivered, therapist-supported therapy in child and mental health services across various diagnoses and research sites (2007-2025)^1^

^1^Regression test for funnel plot asymmetry: b = 24.6 (CI: 16.3, 32.9), p > 0.05


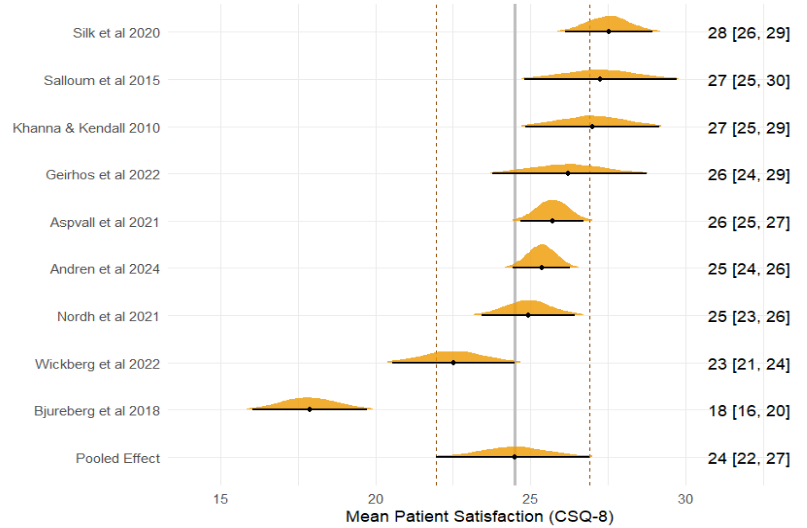
 Figure 7. Forest plot of mean patient satisfaction level from the client satisfaction questionnaire (CSQ-8, 8-31) (Credible Interval, CI), from Bayesian random-effects meta-analysis model of reviewed studies on implementation of internet-delivered, therapist-supported therapy in child and mental health services across various diagnoses and research sites (2007-2025), stratified by study design.^1^

^1^ f= Y|seY ~ 1 + (1|Study)

Y ~ Normal (20, 4)

Tau ~ half-Cauchy(0, 4)


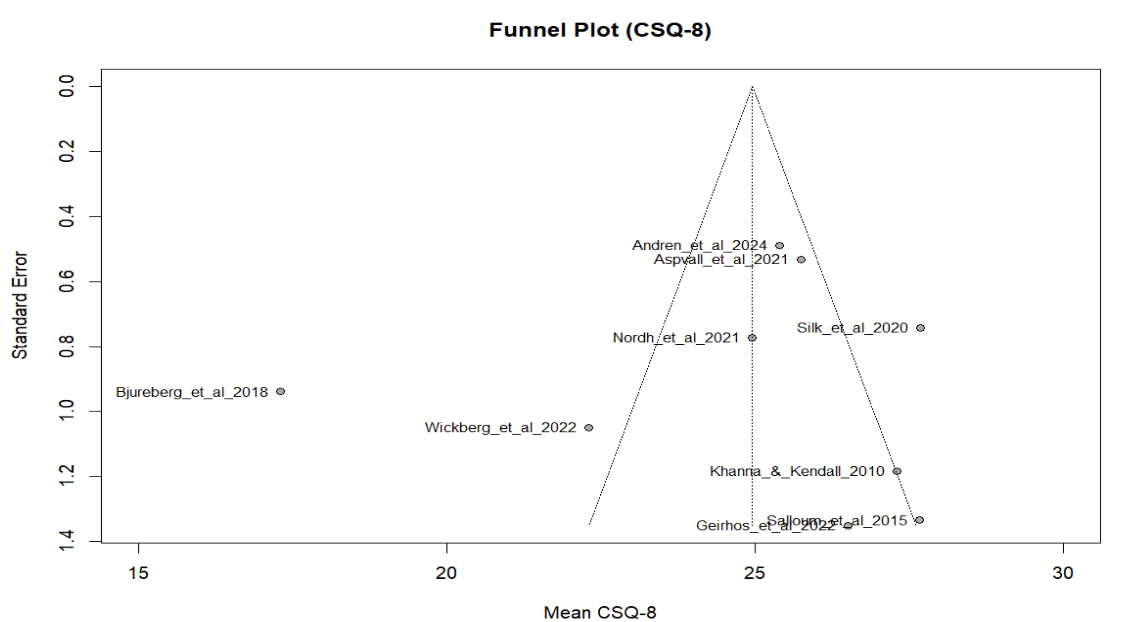
 Figure 8. Funnel plot of mean patient satisfaction level from the client satisfaction questionnaire (CSQ-8, 8-31) of reviewed studies on implementation of internet-delivered, therapist-supported therapy in child and mental health services across various diagnoses and research sites (2007-2025) ^1^

^1^Regression test for funnel plot asymmetry: b = 25.2 (CI: 17.0, 33.2), p > 0.05


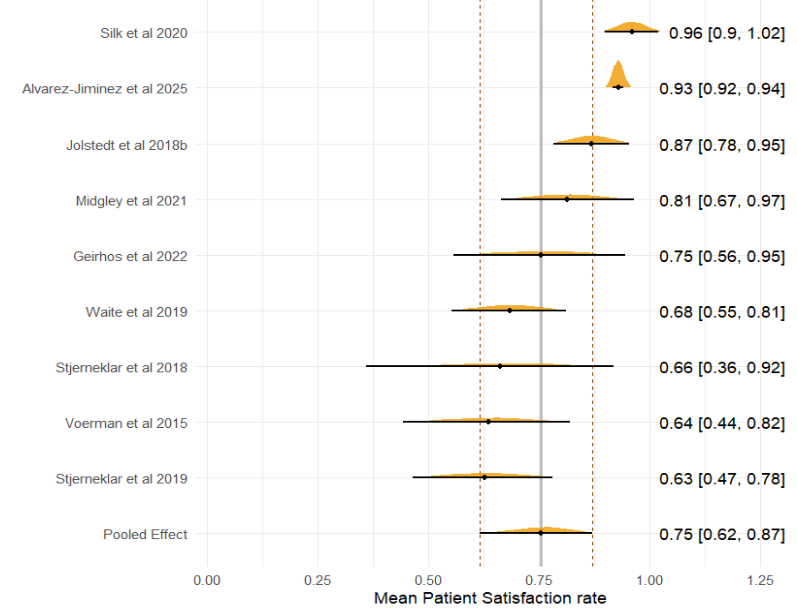
 Figure 9. Forest plot of mean patient satisfaction rating (Credible Interval, CI), from Bayesian random-effects meta-analysis model of reviewed studies on implementation of internet-delivered, therapist-supported therapy in child and mental health services across various diagnoses and research sites (2007-2025), stratified by study design.^1^

^1^ f= Y|seY ~ 1 + (1|Study)

Y ~ Normal (0.55, 0.2)

Tau ~ half-Cauchy(0, 0.2)


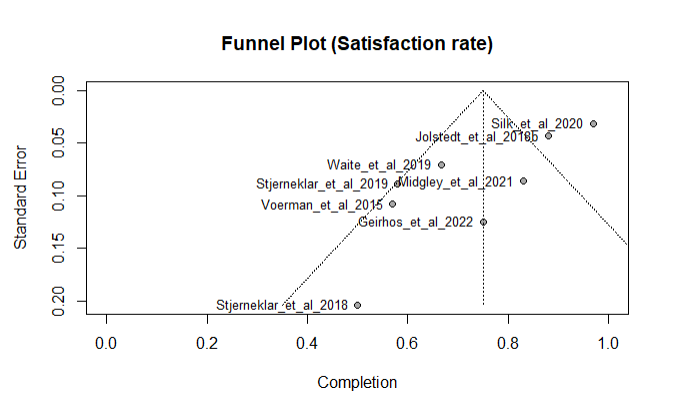
 Figure 10. Funnel plot of mean patient rated satisfaction of reviewed studies on implementation of internet-delivered, therapist-supported therapy in child and mental health services across various diagnoses and research sites (2007-2025) ^1^

^1^Regression test for funnel plot asymmetry: b = 0.98 (CI: 0.86, 1.08), p < 0.01
