# Supplementary material for: Barriers to and Facilitators of Implementation of Internet-Delivered Therapist-Guided Therapy in Child and Adolescent Mental Health Services: Systematic Review and Bayesian Meta-Analysis
Source: J Med Internet Res. 2025 Dec 22;27:e83543. doi: 10.2196/83543 (PMC12721491; doi:10.2196/83543)
Supplement: Multimedia Appendix 9 [file jmir-v27-e83543-s009.docx]

Appendix 9 – Sensitivity analyses

Table 1: Table of results: Pooled parameter estimates of implementation outcomes (mean and Credible Interval (CI) and study heterogeneity (Tau)) from Bayesian random-effects meta regression-analyses models with multiple imputation and weakly informative priors of reviewed studies in Europe, America and Asia from 2007 to 2025 on implementation of internet-delivered, therapist-guided therapy in child and adolescent mental health services, stratified by time as a continuous variable from 0-1 over the range in the included studies (N=46)^[[1]](#footnote-2)^

| Model and parameter | Posterior mean | 95% CI |
| --- | --- | --- |
| Treatment Dropout (n=46 studies, 0% missing)^2,3^ |  |  |
| Overall estimate | 0.18 | 0.06; 0.43 |
| Time | 0.02 | -0.12; 0.39 |
| Between-study heterogeneity estimate for Time=0 | 0.41 | 0.37; 0.56 |
| Between-study heterogeneity estimate for Time=1 | 0.41 | 0.31; 0.58 |
| Adjusted Proportion of Modules Completed (w/ multiple imputation, n= 46 studies) |  |  |
| Overall estimate (proportion) | 0.69 | 0.49; 0.87 |
| Time | -0.01 | -0.26; 0.25 |
| Between-study heterogeneity estimate | 0.20 | 0.15; 0,26 |

Table 2. Table of results: Pooled parameter estimates of implementation outcomes (mean and Credible Interval (CI) and study heterogeneity (Tau)) from Bayesian random-effects meta regression-analyses models with weakly informative priors of reviewed studies in Europe, America and Asia from 2007 to 2025 on implementation of internet-delivered, therapist-guided therapy in child and adolescent mental health services stratified by design (Randomized Controlled Trial (RCT)=1 and Non-RCT studies =0) (N=46)^[[2]](#footnote-3)^

| Model and parameter | Posterior mean | 95% CI |
| --- | --- | --- |
| Treatment Dropout (n=46 studies, 0% missing)^2,3^ |  |  |
| Overall estimate | 0.24 | 0.15; 0.35 |
| RCT | -0.10 | -0.17; 0.04 |
| Between-study heterogeneity estimate for non-RCT | 0.46 | 0.37; 0.59 |
| Between-study heterogeneity estimate for RCT | 0.34 | 0.27; 0.45 |
| Adjusted Proportion of Modules Completed (w/ multiple imputation, n= 46 studies) |  |  |
| Overall estimate (proportion) | 0.68 | 0.58; 0.77 |
| RCT | 0.01 | -0.01; 0.16 |
| Between-study heterogeneity estimate | 0.19 | 0.15; 0.25 |

Table 3. Table of results: Pooled parameter estimates of implementation outcomes (mean and Credible Interval (CI) and study heterogeneity (Tau)) from Bayesian random-effects meta regression-analyses models with weakly informative priors of reviewed studies in Europe, America and Asia from 2007 to 2025 on implementation of internet-delivered, therapist-guided therapy in child and adolescent mental health services stratified by definition of dropout (1= strict definition (not completing 100%), 0= other definitions) (N=46)^[[3]](#footnote-4)^

| Model and parameter | Posterior mean | 95% CI |
| --- | --- | --- |
| Treatment Dropout (n=46 studies, 0% missing)^2,3^ |  |  |
| Overall estimate | 0.15 | 0.08; 0.23 |
| Strict definition of dropout (100% completion) | 0.13 | 0.00; 0.32 |
| Between-study heterogeneity estimate for Strict=0 | 0.36 | 0.27; 0.47 |
| Between-study heterogeneity estimate for Strict=1 | 0.50 | 0.40; 0.63 |

1. *R version 4.1.1, CmdStan, brms package, 4 chains, 4000 iterations (2000 warmup and 2000 post-warmup draws). Multiple imputation models using Predictive Mean Matching from the Mice package with 20 samples*

   *^2^ 4 studies excluded from the analysis due to study design (focus group study), not missing at random. ^3^ Transformed from log odds to probability scale.* [↑](#footnote-ref-2)
2. *R version 4.1.1, CmdStan, brms package, 4 chains, 4000 iterations (2000 warmup and 2000 post-warmup draws). Multiple imputation models using Predictive Mean Matching from the Mice package with 20 samples*

   *^2^ 4 studies excluded from the analysis due to study design (focus group study), not missing at random. ^3^ Transformed from log odds to probability scale* [↑](#footnote-ref-3)
3. *R version 4.1.1, CmdStan, brms package, 4 chains, 4000 iterations (2000 warmup and 2000 post-warmup draws). Multiple imputation models using Predictive Mean Matching from the Mice package with 20 samples*

   *^2^ 4 studies excluded from the analysis due to study design (focus group study), not missing at random. ^3^ Transformed from log odds to probability scale* [↑](#footnote-ref-4)
